# Ketoxime peptide ligations: oxidative couplings of alkoxyamines to *N*-aryl peptides†
†Electronic supplementary information (ESI) available: Experimental details; characterization data for peptides and ketoxime products; LCMS chromatograms and NMR spectra. See DOI: 10.1039/c9sc04028e


**DOI:** 10.1039/c9sc04028e

**Published:** 2019-08-29

**Authors:** Quibria A. E. Guthrie, Hailey A. Young, Caroline Proulx

**Affiliations:** a Department of Chemistry , North Carolina State University , Raleigh , NC 27695-8204 , USA . Email: cproulx@ncsu.edu

## Abstract

Chemoselective ligation methods that preserve or introduce side chain diversity are critical for chemical synthesis of peptides and proteins. Starting from ketone substrates instead of aldehydes in oxime ligation reactions would allow substitution at the site of ligation; however, synthetic challenges to readily access ketone derivatives from common amino acid building blocks have precluded the widespread use of ketoxime peptide ligation reactions thus far. Moreover, ketones are typically much slower to react in condensation reactions compared to aldehydes. Here, one-pot catalyst-free oxidative couplings of α-substituted *N*-aryl peptides with alkoxyamines provide access to oxime linkages with diverse side chains. Electron-rich *N*-(*p*-Me_2_N-phenyl)-amino acids possessing substituents at the α-carbon were found to be uniquely capable of undergoing site-selective α-C–H oxidations *in situ* under an O_2_ atmosphere at neutral pH. Comparative studies with *N*-arylglycinyl peptides revealed that substitution at the α-carbon caused notable changes in reactivity, with greater sensitivity to solvent and buffer salt composition.

## Abstract 2

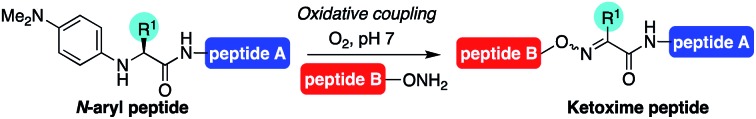
Ketoxime peptides are readily accessible from oxidative couplings between *N*-aryl peptides and alkoxyamines under catalyst-free conditions.

## Introduction

**Scheme 1:**
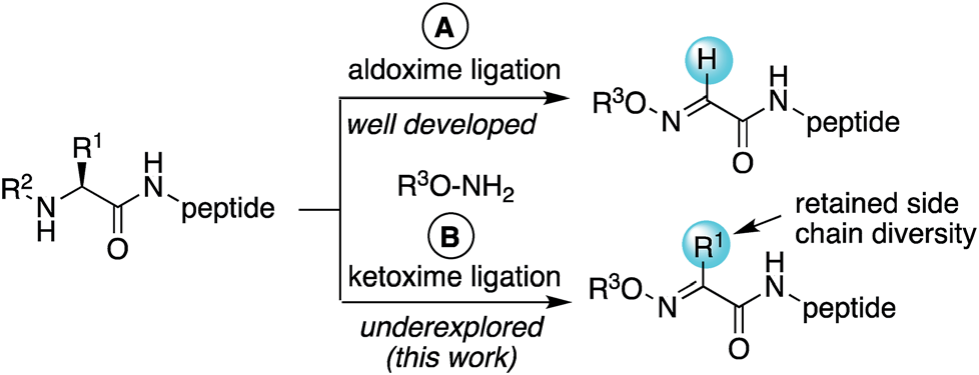
Aldoxime (a) *vs.* ketoxime (b) peptide ligations.

Oxime ligation reactions,^1^ traditionally obtained from aniline-catalyzed couplings of carbonyl compounds with alkoxyamines,^2^ have been widely used in chemoselective functionalization of biomolecules. Among other applications, reactions of ketones or aldehydes with α-nucleophiles can be used in conjunction with other bioconjugation reactions^3^ for cell surface imaging,^4^ to create protein–polymer conjugates,^5^ promote peptide macrocyclizations,^6^ and ligate two or more peptide fragments together.2a,^7^ Moreover, oxime bonds exhibit greater stability compared to hydrazone linkages,^8^ and have been found to be tolerated within β-turn secondary structures6*g* and stabilize α-helical conformations in macrocyclic peptides.6*f* Despite their widespread utility, most applications have been limited to aldoxime bond formation devoid of side chain functionality (Scheme 1a). Comparatively little is known about the impact of diverse ketoxime bonds (Scheme 1b, R^1^ ≠ H) on peptide structure and conformation, and methods to access them in high yields under mild conditions are currently lacking.

**Scheme 2:**
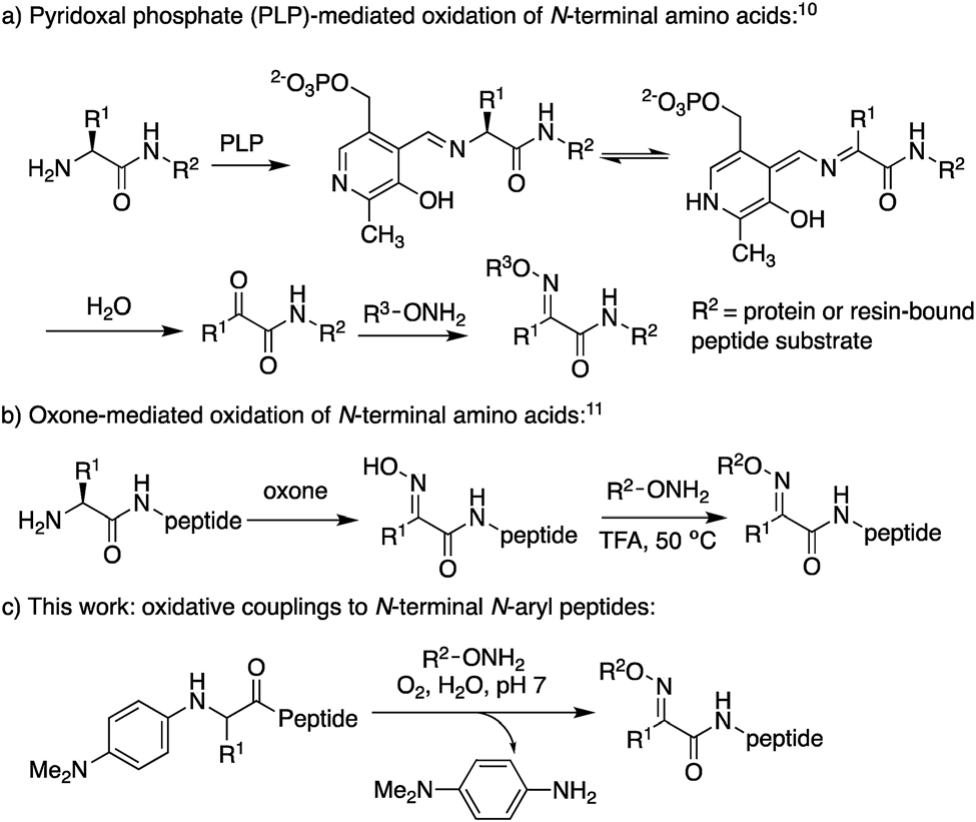
Strategies to access ketoxime peptides, where R^1^ ≠ H.

Retaining or expanding side chain functionality at the ligation site is desirable to efficiently mimic native peptide sequences, allow for rapid structure–activity relationship (SAR) studies of the linker itself,^9^ and to tune the structural properties of the oxime bond. To preserve side chain functionality in oximes, pyridoxal 5′-phosphate (PLP)-mediated oxidation of N-terminal residues in peptides/proteins can be used to generate the corresponding ketone substrates (Scheme 2a).^10^ However, decreased reactivity of ketones *vs.* aldehydes is often observed, and removal of excess PLP can be problematic when using peptide substrates.10*b* Alternatively, treatment of peptides with oxone can be used to site-selectively oxidize the N-terminus, albeit harsh conditions are required for the subsequent oxime exchange reaction (Scheme 2b).^11^

Here, we describe the direct access to ketoxime bonds in high yields *via* a one-pot, catalyst-free *in situ* oxidation and coupling of *N*-aryl peptides to alkoxyamine nucleophiles (Scheme 2c), using O_2_ as the only oxidant. Key to the success of this approach is the use of *N*-(*p*-dimethylaminophenyl) peptides as substrates, which readily oxidize to a transient ketimine in mild aerobic conditions. We further demonstrate that this oxidative coupling is uniquely dependent on solvent composition, with high yielding reactions conveniently occurring under aqueous conditions at neutral pH with a variety of *N*-aryl amino acids. Significantly, while site-selective α-C–H functionalizations of *N*-phenylglycine derivatives^12^ have continued to emerge since the first report in 2008,12a,b including metal-free aerobic couplings of indole and styrene derivatives,12*c* to the best of our knowledge there are no prior examples of oxidative couplings to amino acid derivatives other than glycine. Considering the utility of chemoselective ligation and the relevance of α-C–H oxidation in synthetic chemistry, this method should find widespread use in the broad field of amino acid and peptide functionalization.

## Results and discussion

### Synthesis of *N*-aryl peptides

**Scheme 3:**
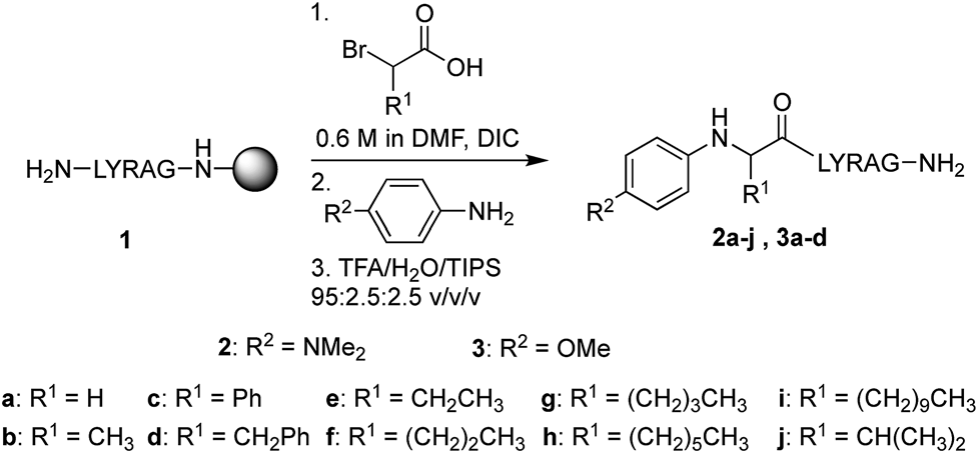
Synthesis of Cα-substituted *N*-aryl amino acid-terminated peptides.

In our initial quest to investigate reactivity of Cα-substituted *N*-aryl amino acids, guided by our previous studies on glycine derivatives,^13^ we pursued both *N*-(*p*-MeO-Ph)- and *N*-(*p*-Me_2_N-Ph) peptides. To probe the effect of side chain chemistry on reactivity, Cα substitution was varied to include a methyl, benzyl, and phenyl substituents, as well as aliphatic side chains of various lengths, giving peptides **2a–j** and **3a–d** (Scheme 3). These were accessed *via* activation and coupling of racemic α-substituted bromoacetic acid derivatives to resin-bound peptides, followed by S_N_2 displacement with the respective aniline derivative using submonomer peptoid synthesis procedures^14^ (Scheme 3). Most analogs were synthesized in good yields, albeit some required heating during the displacement step (ESI†).

### Effect of *N*-aryl ring electronics and side chain

**Fig. 1:**
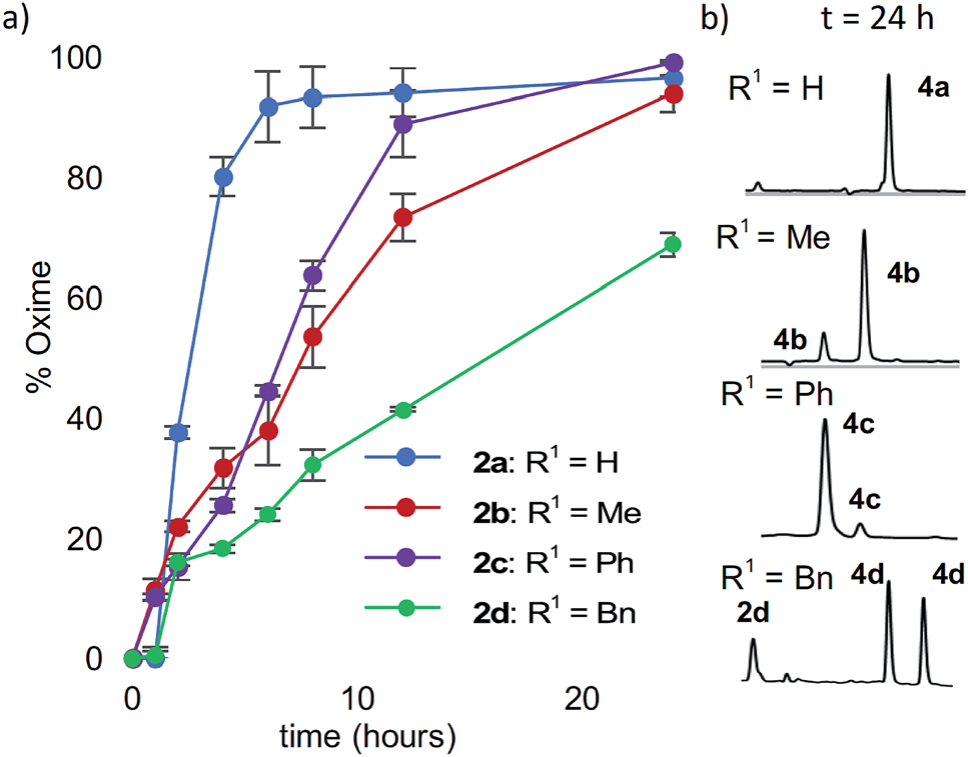
(a) % oxidation and coupling of **2a–d** (1 mM) to *O*-benzylhydroxylamine hydrochloride (5 mM) to give oximes **4a–d** over time under O_2_ atmosphere at pH 7. (b) The extent of the reaction was monitored by LCMS at 214 nm.

Starting with analogs **2b–d** and **3b–d**, which possess markedly different side chains (*i.e.* methyl, benzyl, and phenyl groups), we next assessed their ability to undergo oxidative couplings with *O*-benzylhydroxylamine under an O_2_ atmosphere. Reactivity of *N*-(*p*-Me_2_N-Ph)- and *N*-(*p*-MeO-Ph)-peptides was evaluated at pH 7.0 and pH 4.5, respectively, as these conditions were found to be optimal in oxime ligation reactions with *N*-phenylglycinyl peptide derivatives.^13^ Remarkably, peptides **2b–d** were capable of undergoing clean conversions to the desired ketoximes in varying *E*/*Z* ratios, with no sign of competitive ketone by-product formation (Table 1, entries 2–4). In stark contrast to the apparent facile oxidative coupling to analogs **2b–d**, where R^2^ = NMe_2_, no sign of oxime formation was detected for peptides **3b–d** (R^2^ = OMe) over 24 h (Table 1, entries 6–8), even at pH 4.5. Interestingly, addition of 1 mM potassium ferricyanide^13^ to the reaction mixture also proved unsuccessful at triggering oxidative couplings with these substrates. While we were initially surprised by the complete shutdown of reactivity with *N-p*-methoxyphenyl (PMP)-amino acids, it may explain the lack of literature precedence on oxidative and cross-dehydrogenative couplings (CDC) beyond glycine derivatives using similar substrates.^12^ In contrast, *N*-(*p*-Me_2_N-Ph)-substituted amino acids appear especially prone to undergo a chemoselective oxidation to the α-ketimino amide intermediate. To verify that trace amounts of metal were not required for this reaction to proceed, the reaction was run in the presence of 10 mM EDTA, using a new stir bar and vial (ESI†). This control reaction was found to give the oxime product in similar conversions, supporting the mild, metal-free nature of this bioconjugation reaction. To gain additional insight on reaction rates, oxime conversions were monitored over time and compared to the *N*-(*p*-Me_2_N-Ph)glycine-LYRAG substrate **2a** (Fig. 1). While the reactions rates decreased when substituents were introduced at the α-carbon, the corresponding functionalized oximes **4b–d** were obtained as the major products in varying *E*/*Z* ratios after 24 h in all cases. Analog **2c**, an *N*-aryl phenylglycine derivative, yielded the ketoxime product at a comparable rate relative to the *N*-aryl alanine derivative **2b**. Oxidation of *N*-(*p*-Me_2_N-Ph)phenylalanine-LYRAG **2d** proceeded more slowly, which may be due to increased steric hindrance. The increased reactivity of **2c***vs.***2d** is likely due to the relative stability of transient benzylic radicals and/or cationic intermediates, proposed to lead to the formation of reactive α-ketimino amide species. Of note, *N*-aryl peptides **2b** and **2c** favored the formation of the least polar (**4b**) and more polar (**4c**) isomers as the major products, respectively, while *N*-(*p*-Me_2_N-Ph)F-LYRAG **2d** afforded ∼1 : 1 *E*/*Z* mixtures of ketoxime **4d**. This could ultimately impact peptide secondary structures and provide a convenient handle to access extended and turn structures contingent on the ketoxime substituent.

**Table 1:** Oxime ligation conversions (%) determined by LCMS after 24 h

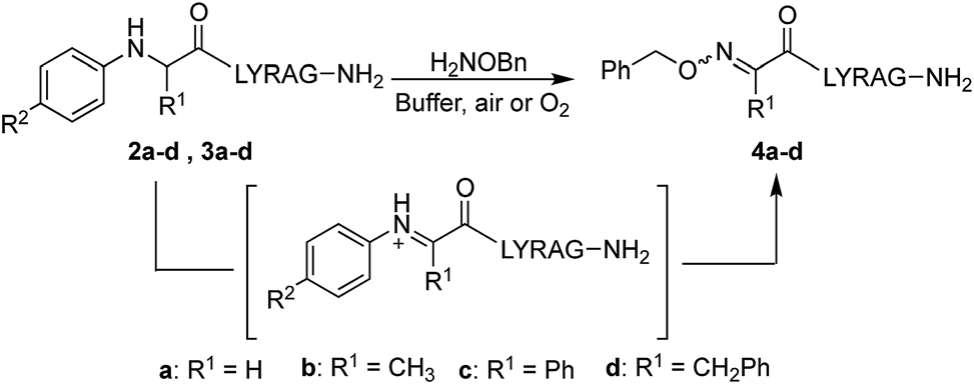		
Entry	Peptides	% Oxime at *t* = 24 h
1	**2a**: R^1^ = H, R^2^ = NMe_2_	>99^*a*^
2	**2b**: R^1^ = CH_3_, R^2^ = NMe_2_	94^*a*^
3	**2c**: R^1^ = Ph, R^2^ = NMe_2_	99^*a*^
4	**2d**: R^1^ = CH_2_Ph, R^2^ = NMe_2_	69^*a*^
5	**3a**: R^1^ = H, R^2^ = OMe	<1^*a*^ (72^*b*^)
6	**3b**: R^1^ = CH_3_, R^2^ = OMe	<1^*a*^ (<1^*b*^)
7	**3c**: R^1^ = Ph, R^2^ = OMe	<1^*a*^ (<1^*b*^)
8	**3d**: R^1^ = CH_2_Ph, R^2^ = OMe	<1^*a*^ (<1^*b*^)

^*a*^Reactions were performed in phosphate buffer pH 7 under an O_2_ atmosphere with 5 mM *O*-benzylhydroxylamine hydrochloride and 1 mM peptide concentrations.

^*b*^Reactions were performed in ammonium acetate buffer pH 4.5 under air with 1 mM *O*-benzylhydroxylamine hydrochloride and 1 mM peptide concentrations.^13^

**Scheme 4:**
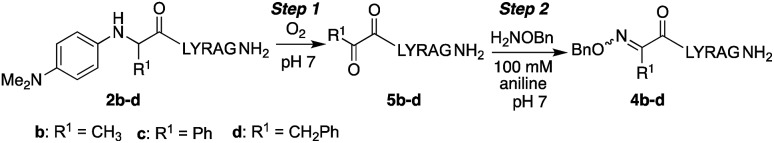
Comparative two-step oxidation/oxime ligation procedure.

In the absence of an alkoxyamine nucleophile, *N*-aryl peptides **2b** and **c** furnished the corresponding α-ketoamides **5b** and **c**; however, upon exposure to *O*-benzylhydroxylamine in a separate step (Scheme 4), ketoximes **4b** and **c** were obtained in much lower crude purity (ESI†), with or without addition of 100 mM aniline catalyst. Interestingly, *N*-aryl peptide **2d** was found to decompose into a mixture of unidentified byproducts in the absence of α-nucleophiles. In line with this, similar ketoxime peptides obtained using PLP-mediated transamination procedures (Scheme 2a, R^1^ = Bn) are only produced in low yields.10*b* Thus, by invaluably precluding the need to isolate a less reactive ketone intermediate, our mild one-pot oxidative coupling conditions significantly increases the scope of side chain diversity in ketoxime peptides. Peptide **2j**, which has a branched isopropyl side chain, was the only analog that failed to react cleanly under our conditions, providing a mixture of oxime (22%), α-ketoamide (37%) and starting material (35%) instead.

### Effect of buffer

**Fig. 2:**
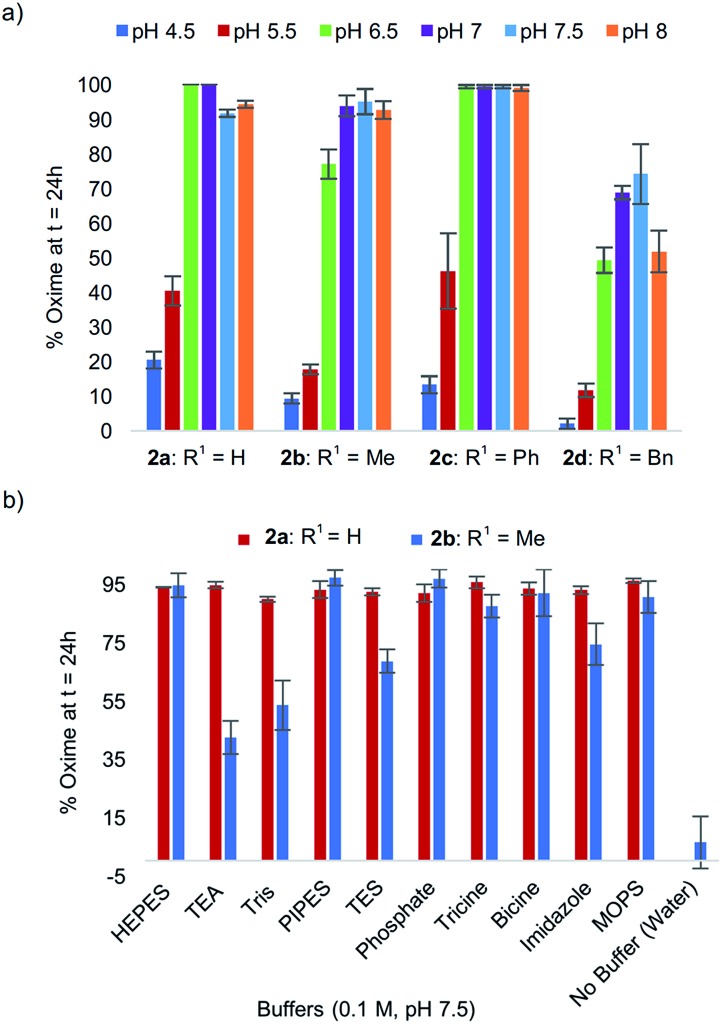
(a) % Oxime formation after 24 h for peptides **2a–d** at pH 4.5–8. (b) Comparative effect of buffer salt composition on oxime conversions at pH 7.5 for peptides **2a***vs.***2b**.

In exploring the effect of pH on reactivity, we observed that the optimal pH conveniently remained near physiological pH for all analogs **2a–d** (Fig. 2a), with peptide analog **2c** proving the least sensitive to pH in the 6.5–8 range. In the course of these studies, we noted that progression of the tandem oxidation/oxime ligation could be influenced by the buffer salt composition. Using peptide **2b** as a representative example, when the reaction was performed at pH 8.5 in a glycine-sodium hydroxide buffer instead of phosphate buffer, no oxime product was detected by LCMS (data not shown). Conversely, running the same reaction in Tris buffer led to 91% oxime formation after 24 h. A similar trend was also found with *N*-phenylglycinyl peptide **2a**.

To study this buffer salt composition effect further, ten different buffers were screened while maintaining the optimal pH of 7.5 constant, using both *N*-(*p*-Me_2_N-Ph)G-LYRAG **2a** and *N*-(*p*-Me_2_N-Ph)A-LYRAG **2b** (Fig. 2b). Interestingly, we found the *N*-aryl glycine derivative **2a** to be less sensitive to buffer salt composition compared to *N*-aryl alanine peptide **2b**. In the latter case, TEA and Tris-based buffers were shown to have a negative impact on reaction rates; however, oxime products were still obtained in 42–56% conversions after 24 h.‡
‡As noted earlier, >90% oxime formation was observed in Tris buffer at pH 8.5, indicating that Tris buffer may have a different optimal pH value compared to phosphate buffer. For both peptides **2a** and **2b**, the oxime ligation reaction was almost completely inhibited when the reaction was run without buffer in ultrapure water. Phosphate buffer concentrations between 50–100 mM were later found to be required for near quantitative oxidative couplings to **2b** after 24 h, with a significant drop in reactivity when the phosphate buffer concentration was ≤25 mM (ESI†).

Because experiments run at pH 4.5 were also performed in a different (ammonium acetate) buffer, the oxidation/ligation reaction was reinvestigated at pH 4.5, 7, and 8.5 using the Britton–Robinson so-called universal buffer for both **2a** and **2b**. This confirmed the previously observed trends, where decreased reactivity was observed at pH 4.5, whereas pH 7 and 8.5 both afforded oxime ligation product in >80% conversion after 24 h (ESI†).

### Alkyl chain length variation in N-aryl amino acids and effect of organic solvent

**Fig. 3:**
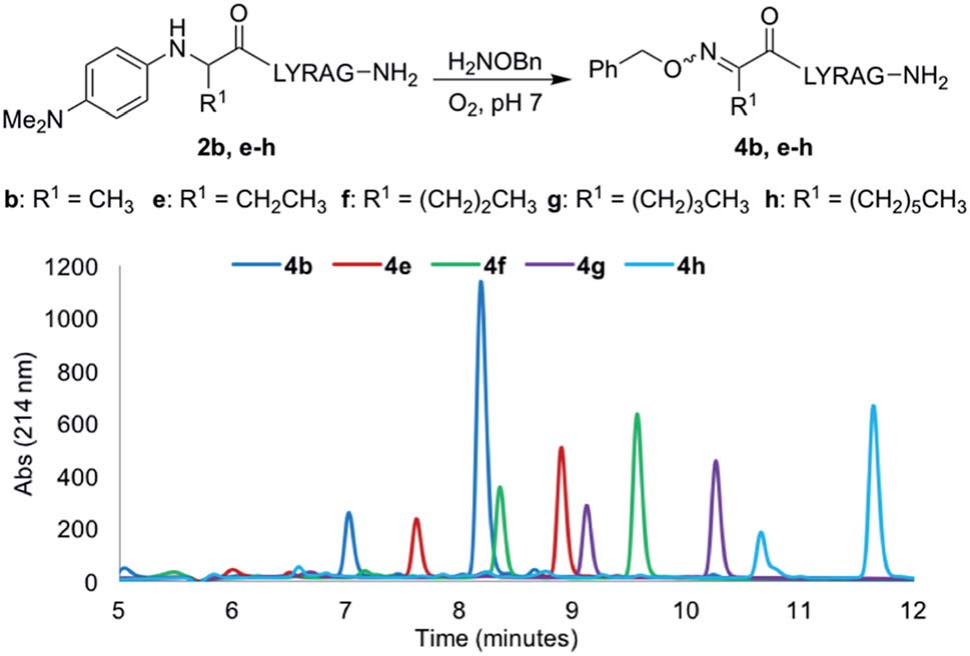
Comparative LCMS chromatograms for analogs **2b–h** using a 10–30% gradient of CH_3_CN (0.1% TFA) in H_2_O (0.1% TFA) over 12 minutes.

Increasing the alkyl chain length from a methyl to a decyl group would provide a convenient handle to modulate log P properties of oxime-linked peptides, without affecting oxime ligation yields. Thus, analogs **2e–h** were next evaluated under our optimal oxime ligation conditions (O_2_, phosphate buffer pH 7). Gratifyingly, all analogs provided the desired *E*/*Z* ketoximes as the major products with no detectable side products, with the expected increase in analytical HPLC retention times as a function of alkyl chain length (Fig. 3).§
§The analog **4i** with a decyl side chain did not go to completion and was excluded from Fig. 3. However, changing the side chain from a methyl (**2b**) to a hexyl (**2h**) and decyl group (**2i**) required addition of organic solvents to increase solubility and revealed another interesting trend, where the oxime ligation conversions after 24 h decreased with increasing amounts of organic solvent (Table 2, entries 4 and 5).

**Table 2:** Ketoxime ligation conversions (%) and effect of organic solvent composition on for analogs **2e–i**

Entry	Peptides	Oxidation conditions	% Oxime^*a*^ at *t* = 24 h
1	**2e**: R^1^ = CH_2_CH_3_	pH 7, O_2_	94
2	**2f**: R^1^ = (CH_2_)_2_CH_3_	pH 7, O_2_	85
3	**2g**: R^1^ = (CH_2_)_3_CH_3_	pH 7, O_2_	89
4	**2h**: R^1^ = (CH_2_)_5_CH_3_	25% EtOH in pH 7 buffer, O_2_	62
5	**2h**: R^1^ = (CH_2_)_5_CH_3_	50% EtOH in pH 7 buffer, O_2_	27
6	**2i**: R^1^ = (CH_2_)_9_CH_3_	50% EtOH in pH 7 buffer, O_2_	25

^*a*^The extent of the reaction was monitored by LCMS at 214 nm.

**Fig. 4:**
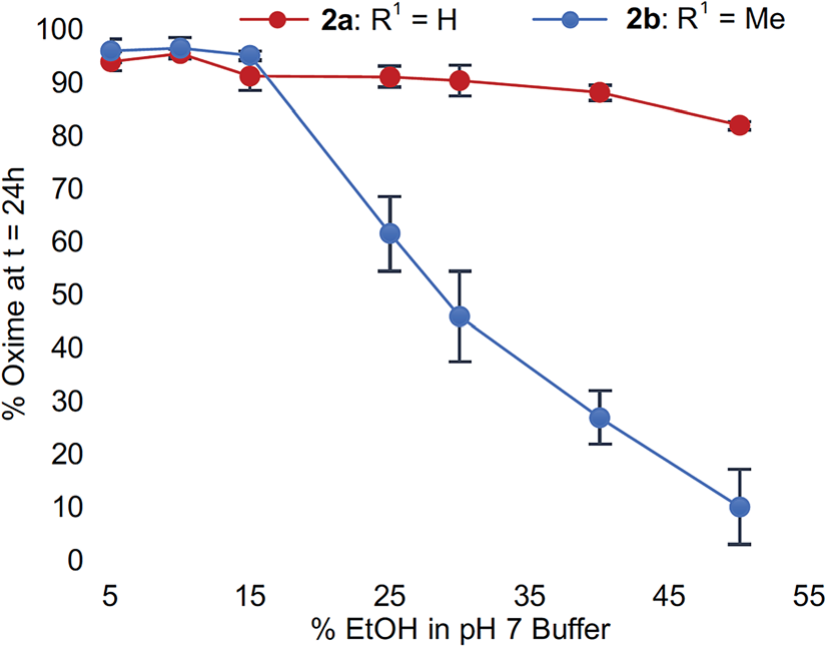
% Oxime conversions as a function of ethanol composition for **2a** and **2b**.

To confirm this trend, we compared the oxime conversions as a function of ethanol content for analogs **2a***vs.***2b** (Fig. 4). Once again, much like what we observed for the buffer salt studies, we found that Cα-substitution in *N*-aryl amino acids led to greater sensitivity to organic solvent composition compared to *N*-aryl glycinyl peptide **2a**, which remained virtually unaffected by addition of up to 50% ethanol. In comparison, peptide **2b** provided very little oxime product (<20%) under identical conditions (Fig. 4). Addition of acetonitrile instead of ethanol had similar effects; however, the conversions were slightly higher compared to ethanol (ESI†).

### Mechanistic insight using model compounds

**Scheme 5:**
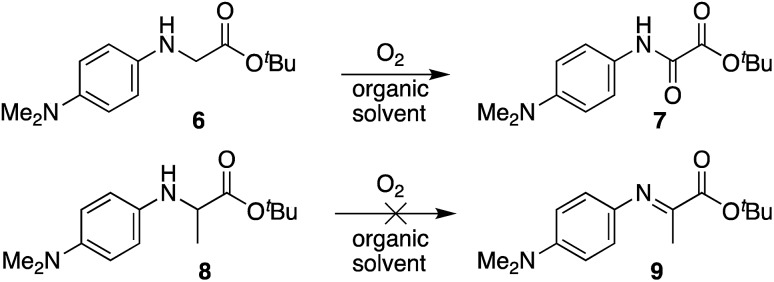
Oxidation of *N*-aryl glycine and *N*-aryl alanine *tert*-butyl esters in organic solvent.

In view of applying these findings beyond oxime ligation chemistry (*e.g.* C–H functionalization), we wanted to explore small molecule model compounds to study their oxidation potential in various organic solvents. As noted earlier, the dimethylamino substituent on the phenyl ring of the *N*-aryl amino acid is critical for oxime ligations to occur in high yields in aqueous buffer at neutral pH. We wondered if the ease of oxidation would readily translate to any organic solvents with *N*-(*p*-Me_2_N-Ph)glycinyl peptides, and if more pronounced solvent effects would be observed in Cα substituted analogs. As such, compounds **6** and **8** were synthesized and oxidation in the absence of alkoxyamines was monitored by NMR (Scheme 5). Specifically, *N*-aryl glycine and *N*-aryl alanine *tert*-butyl esters were dissolved in different solvents, followed by sparging of the solution with oxygen for 30 seconds before leaving the reaction mixture stirring under an O_2_ atmosphere for 24 h at room temperature. In all these cases, we screened a polar protic solvent (EtOH), polar aprotic solvents (THF, DCM), and a 5 : 1 MeCN : DCE solvent mixture.¶
¶This solvent mixture was previously shown to oxidize similar *N*-(*p*-Me-Ph)Gly-OEt substrates into oxalic acid derivatives in the absence of nucleophiles, albeit requiring addition of 0.2 mol% HCl or use of undistilled DCE. See ref. 12*c*. In agreement with our oxime ligation studies, *N*-(*p*-Me_2_N-Ph)Gly-O*t*Bu **6** was found to readily oxidize in the absence of catalysts in all solvents screened, and introduction of a methyl group at the α-carbon in substrate **8** completely inhibited oxidation. It should be noted that an oxalic acid derivative would not be accessible with substrate **8**, and that oxidation to the α-imino ester may require a proton source. However, attempts to add *O*-benzylhydroxylamine hydrochloride or acetic acid to the reaction mixture both proved unsuccessful at triggering oxidation, indicating that the ease of the tandem oxidation/oxime ligation reaction at or near neutral pH is likely driven by a unique buffer salt effect with these substrates (*vide supra*).

### Sequence effects and peptide–peptide ligation

**Fig. 5:**
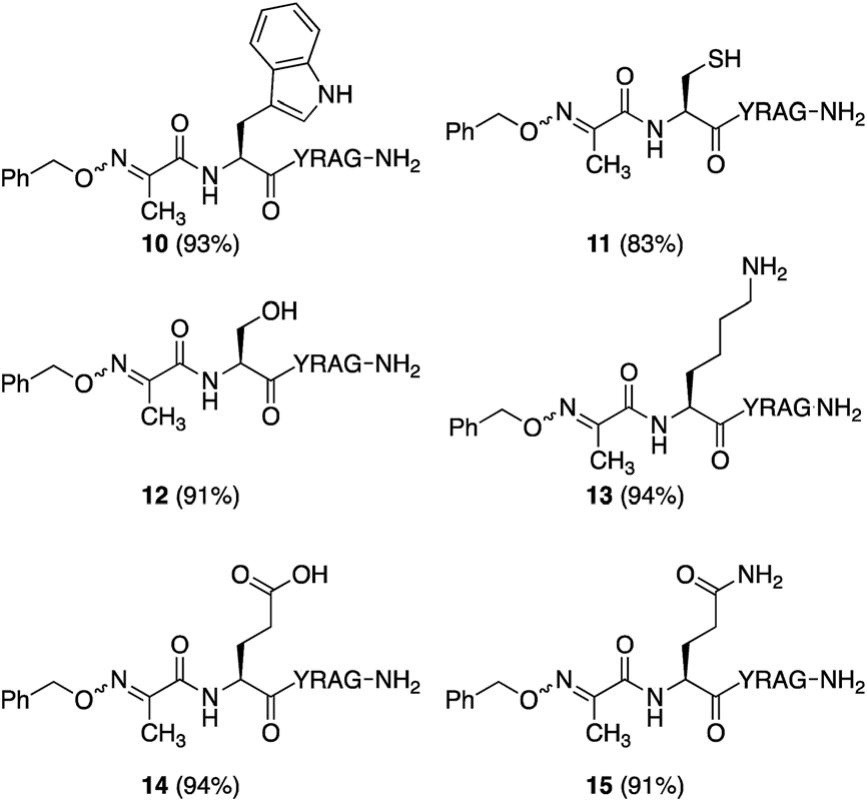
Oxime ligation conversions for peptides containing a wide variety of amino acid side chains.

**Fig. 6:**
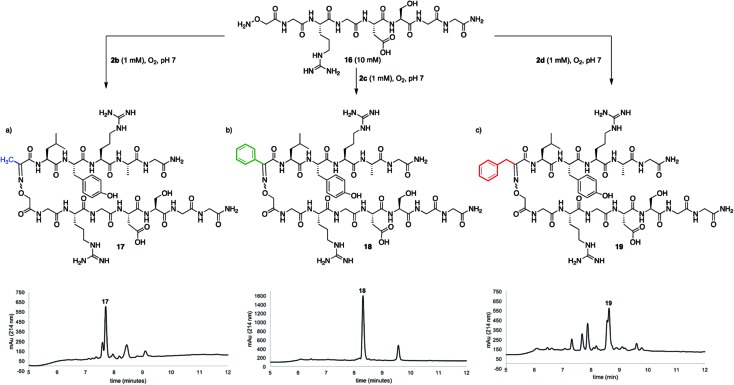
Peptide–peptide ligation providing oxime linkages functionalized with (a) a methyl (**17**), (b) a phenyl (**18**), and (c) a benzyl (**19**) substituent.

While many factors appear important to trigger oxidative couplings with Cα-substituted, *N*-(*p*-Me_2_N-Ph)-amino acids, the reaction proceeds remarkably well in phosphate buffer at neutral pH under an O_2_ atmosphere. These exceptionally mild conditions, together with the *in situ* oxidation/ligation reaction procedure, render a wide array of amino acids compatible with this method, including Trp, Cys, Ser, Lys, Glu, and Gln (Fig. 5). As such, this should find wide applicability in peptide–peptide oxime ligation reactions, providing unique opportunities to retain side chain diversity at the site of ligation. To illustrate this, *N*-aryl amino acids **2b–d** (1 mM) were coupled to aminooxyacetyl-GRGDSGG **16** (10 mM), yielding the corresponding substituted oxime-linked peptides as the major products (Fig. 6). Similarly to what we observed in coupling reactions with *O*-benzylhydroxylamine, reactivity with *N*-aryl phenylalanine analog **2d** proceeded the slowest and required 24 h to go to completion, while complete disappearance of both *N*-arylalanine **2b** and *N*-arylphenylglycine **2c** derivatives was observed after 12 h, with α-ketimino amide hydrolysis to form the α-ketoamide as a minor byproduct. Moreover, ketoximes **18** was strikingly clean and featured again one major isomer by RP-HPLC; **17** was separable from its minor isomer, and **19** was isolated as an *E*/*Z* mixture. Head-to-head peptide ligations offer opportunities to mimic parallel β-sheets, prevalent secondary structure motifs in proteins.^15^ The impact of the ketoxime linkage and substituent on parallel β-sheet stability will be reported in due course.

## Conclusion

In summary, we report that oxidative couplings to Cα-substituted *N*-aryl peptides can occur under mild aqueous conditions to provide ketoxime bonds in the presence of aminooxy groups. This reaction provides the first examples of (**1**) a one-pot, catalyst-free synthesis of ketoxime peptides in high conversions and (**2**) site-selective α-C–H oxidation of amino acids beyond glycine derivatives. We further uncovered that such reactions are highly dependent on solvent and buffer salt composition, yet provide optimal coupling yields in ideal bioconjugation conditions using phosphate buffers at pH 6.5–8. These optimal conditions translate to a wide variety of side chain chemistry (*e.g.* alkyl, aryl, benzyl), allowing oxime bonds to better mimic amino acids that possess both aromatic and aliphatic side chains. This should expand their utility in peptide and protein synthesis, allowing rapid SAR studies of oxime linkages to modulate peptide properties such as cell permeability. Ketoxime substituents should further provide unique handles to tune peptide secondary structure contingent on *E*/*Z* ratios, and offer opportunities to mimic parallel β-sheets.

## Conflicts of interest

There are no conflicts to declare.

## Supplementary Material

Supplementary information
