# Supplementary material for: Ketoxime peptide ligations: oxidative couplings of alkoxyamines to N-aryl peptides
Source: Chem Sci. 2019 Aug 29;10(41):9506–12. doi: 10.1039/c9sc04028e (PMC7017874; doi:10.1039/c9sc04028e)
Supplement: Supplementary file 1 [file SC-010-C9SC04028E-s001.pdf]

Electronic Supplementary Material (ESI) for Chemical Science.
This journal is © The Royal Society of Chemistry 2019

Guthrie, Q. A. E.; Young, H. A.; Proulx, C.

SUPPORTING INFORMATION

Ketoxime peptide ligations: oxidative couplings of alkoxyamines to *N*-aryl peptides

Quibria A. E. Guthrie, Hailey A. Young, and Caroline Proulx*

Department of Chemistry, North Carolina State University, Raleigh, NC 27695-8204, USA.

e-mail: cproulx@ncsu.edu

Table of Contents of the Supporting Information

Title, authors and address S1

Table of Content S2

1. **General Methods**

   General S3
   Reagents S3-S4

2. **Experimental Procedures and Characterization Data**

   Fmoc-based SPPS: Fmoc deprotection and HBTU couplings S4
   Incorporation of *N*-terminal *N*-aryl amino acid residues S4
   Synthesis of aminooxyacetyl-GRGDSGG S5
   Cleavage test of resin-bound peptides S5
   Full cleavage S5
   Analysis and purification of peptides **2a-j, 3a-d** and aminooxyacetyl-GRGDSGG **16** S5
   Table S1. Characterization data for *N*-aryl amino acid-terminated peptides and aminooxyacetyl-GRGDSGG **16** S6
   Oxime ligation reactions procedures S6-S12
   Table S2. Comparison of oxidative coupling conversions in different buffers at pH 8.5. S9
   Table S3. Control studies to compare buffer composition while keeping pH constant. S9
   Table S4. Comparative reactivity of **2a-b** with varying potassium phosphate buffer concentration at pH 7. S10
   Table S5. Comparative reactivity for peptide **2b** in varying % MeCN *vs* EtOH in phosphate buffer pH 7. S11
   Table S6. Characterization data for *N*-aryl A-XYRAG peptides S12
   Solution Phase Synthesis Procedures S13
   Table S7. *N*-(*p*-NMe_2-Ph)glycine *tert*-butyl ester oxidation in organic solvents S14

3. **LCMS chromatograms**

   N-aryl amino acid-terminated peptides and aminooxyacetyl-GRGDSGG S15-S24
   Oxime ligation reactions S25-S44

4. **NMR spectra** S44-S57

5. **References** S58

1. General methods

General: Polystyrene Rink Amide resin (0.78 mmol/g) was purchased from Protein Technology, Inc™, and the manufacturer's reported loading of the resin was used in the calculation of the yields. Solid phase peptide synthesis (SPPS) was performed using an automated Biotage Syro Wave™ peptide synthesizer in 10 mL parallel reactors with PTFE frits. Incorporation of Boc-aminooxy acetic acid and *N*-aryl amino acids were performed manually in disposable filter columns with 20 μM PE frit filters and caps purchased from Applied Separations (cat # 2413 and 2416 for 3 mL and 6 mL filter columns, respectively) with gentle agitation on a Thermo Fisher vortex mixer equipped with a microplate tray. Solution draining and washing of the resin was done by connecting the filter columns to a water aspirator vacuum *via* a waste trap. For heated reactions, a Fisher Scientific Ultrasonic bath 9.5L (Model # 15337425) was used to heat and agitate the resin. Analytical LCMS analyses were performed using an Agilent Technologies 1260 Infinity II series LCMS Single Quad instrument with ESI ion-source and positive mode ionization, equipped with a 5 μM, 150 x 4.6 mm C18 Vydac column purchased from Mac-Mod Analytical, Inc (cat # 218TP5415). A flow rate of 0.5 mL/min and 5-95% gradient of CH_3CN [0.1% trifluoroacetic acid (TFA)] in water (0.1% TFA) over 12 minutes (total run time = 22 minutes) were used for all LCMS analyses. Peptides were purified on a preparative HPLC (Agilent 218 purification system) using a preparative column (10-20 μM, 250 mm x 22 mm, C18 Vydac column, cat # 218TP101522) at a flow rate of 10 mL/min with gradients of CH_3CN [0.1% trifluoroacetic acid (TFA)] in water (0.1% TFA) over 30 minutes (total run time = 60 minutes).

All solution phase reactions were performed in oven-dried glassware sealed with microwave caps or rubber septa and were stirred with Teflon-coated magnetic stir bars. Tetrahydrofuran (THF) was dried by passage over a column of activated alumina (JC Meyers Solvent System). Thin layer chromatography (TLC) was performed using Silicycle silica gel 60 F-254 precoated plates (0.25 mm) and were visualized by exposure to ultraviolet light (UV) and/or submersion in aqueous ninhydrin solution. Samples were purified using a Biotage® Isolera One, employing polypropylene cartridges preloaded with silica gel (25 micron) and were eluted with UV detection (254, 280 nm). Nuclear magnetic resonance (NMR) spectra (^1H, ^{13}C) were recorded on a 600 MHz Bruker spectrometer at 24 ºC for compounds **6-8** and a 700 MHz Bruker spectrometer for ketoximes **4b-c** and **17-19**. Chemical shifts are expressed in parts per million (ppm, δ scale) and are referenced to residual protium in the NMR solvent ($CHCl_3$, δ 7.26). Data are represented as follows: chemical shift, multiplicity (s = singlet, d = doublet, t = triplet, q = quartet, m = multiplet, br = broad), coupling constant in Hertz, and integration. Chemical shifts for ^{13}C NMR spectra are recorded in parts per million (ppm, δ scale) and are referenced to the central peak of deuterochloroform (δ 77.16). Ketoximes **4b-d** and **17-19** were prepared as 1-3 mM solutions in D_2O or CD_3OD. ^1H NMR for the ketoximes **4b-d** and **17-19** were run with water suppression using Bruker's noesygppr1d experiment method. All spectra were obtained with complete proton decoupling. Infrared (IR) spectra were collected on a Thermo Scientific Nicolet iS5 FTIR instrument using attenuated total reflectance (ATR) mode and signals are reported in reciprocal centimeters (cm^{-1}). Only selected IR frequencies are reported. Melting points were obtained on a Mettler Toledo MP50 One Click Melting Point System.

Reagents: Amino acids, *N,N*-diisopropylethylamine (DIEA), triisopropylsilane (TIPS), bromoacetic acid, and diisopropylcarbodiimide (DIC) were purchased from Chem Impex Int'l, Inc. Reagents such as piperidine, *O*-benzylhydroxylamine hydrochloride, 1,2-ethanedithiol, *N*-hydroxysuccinimide, *p*-anisidine, *N,N*-dimethyl-*p*-phenylenediamine, 2-bromopropionic acid, 2-bromohexanoic acid, 2-bromovaleric acid, 2-bromododecanoic acid and 2-bromooctanoic acid were purchased from Sigma Aldrich. Sodium hydroxide, ammonium acetate, glacial acetic acid,

potassium phosphate monobasic and dibasic, trifluoroacetic acid (TFA), disodium ethylenediaminetetraacetate (EDTA) and solvents were purchased from Fisher Chemical. *N,N,N',N'*-Tetramethyl-*O*-(1H-benzotriazol-1-yl)uronium hexafluorophosphate (HBTU) was purchased from Oakwood Chemicals. 2-bromo-3-phenylpropionic acid was purchased from Matrix Scientific. *Tert*-butyl bromoacetate, 2-bromobutyric acid, 2-bromo-3-methylbutyric acid, DL-alpha-bromophenylacetic acid, and phosphoric acid (85% in water) were purchased from Acros Organics. *Tert*-butyl 2-bromopropanoate was purchased from TCI America. All chemicals were used as received without further purification. Tris (1.0 M, catalog # AAJ60636K2), TEA (0.2 M, catalog # AAJ61319AK), HEPES (1.0 M, catalog # AAJ60712AK), TES (0.2 M, catalog # AAJ61350AE), PIPES (1.0 M, catalog # AAJ62494AK), Phosphate (0.5 M, catalog # AAJ63349AK), MOPS (1.0 M, catalog # AAJ61843AK), Tricine (0.5 M, catalog # AAJ62672AE), Bicine (0.5 M, catalog # AAJ60494AE), and imidazole (0.5 M, catalog # AAJ63496AE) buffers and *o*-boric acid were all purchased from Alfa Aesar.

2. Experimental Procedures and Characterization Data

Fmoc-based SPPS: Fmoc deprotection and HBTU couplings

Peptide syntheses were performed on Polystyrene Rink Amide resin (0.78 mmol/g) using standard manual solid phase peptide synthesis protocols (SPPS) on an automated shaker or using a Biotage Syro Wave™ peptide synthesizer. Couplings of amino acids (3 equiv) were performed in DMF using HBTU (3 equiv) as coupling reagent and DIEA (6 equiv) as base. Fmoc deprotections were performed by treating the resin with 20% piperidine in DMF (v/v) for 5 minutes, followed by treatment with a fresh solution of 20% piperidine in DMF (v/v) for 15 minutes. Resin was washed after each coupling and deprotection reaction with DMF (3 x 1 mL). Prior to cleavage from the resin or storage, resin was washed with CH_2Cl_2 (3 x 1 mL).

Incorporation *N*-terminal *N*-aryl amino acid residues

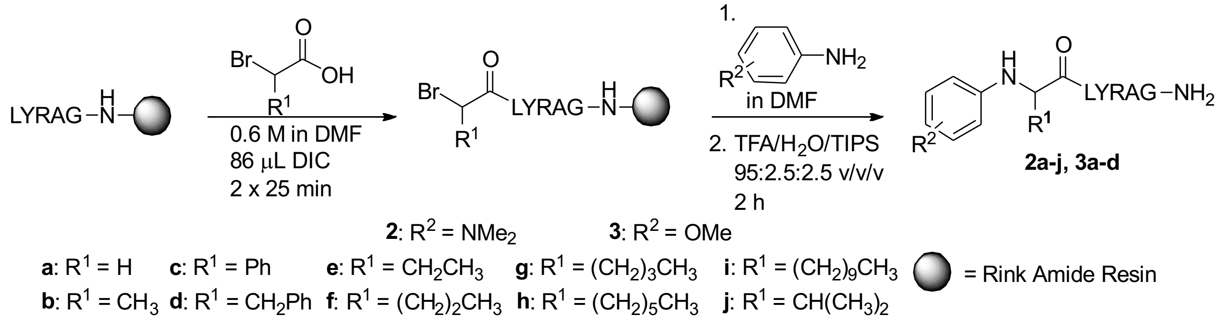

Peptides **2a** and **3a** (R^1 = H) were synthesized based on previous methods.[1] Peptides **2b-j** and **3b-d** were synthesized using slightly modified procedures. Specifically, resin-bound Fmoc-LYRAG (**1**, 100 mg, 0.078 mmol) was transferred to a 3 mL disposable filter column, treated with 1 mL of a 20% piperidine in DMF solution (v/v) and gently agitated for 5 minutes, followed by another 15 minutes incubation with fresh reagent (1 mL). The resin was washed with DMF (5 x 1 mL) and treated sequentially with a 0.6 M solution of the corresponding racemic α-substituted bromoacetic acid derivatives in DMF (1 mL, 0.6 mmol) and 86 μL of *N,N*-diisopropylcarbodiimide (DIC, 0.56 mmol) for 25 minutes. The bromoacetylation procedure was repeated a second time with fresh reagent and the resin was washed with DMF (5 x 1 mL). The resin was then treated with a 2 M solution of 4-(dimethylamino)aniline or 4-methoxyaniline in DMF for 3 hours at room temperature. For peptides **2d** and **3d** (R^1 = CH_2Ph), an additional 3 hours with fresh reagents at

room temperature was required. For peptide **2j** (R = $CH(CH_3)_2$), the displacement required heating with sonication to 60°C for 24 h.

Synthesis of aminooxyacetyl-GRGDSGG
Peptide aminooxyacetyl-GRGDSGG (**16**) was synthesized based on the previous methods.[1-2] (Boc-aminooxy)acetic acid (60 mg, 0.31 mmol, 4 equiv), DIC (48 µL, 0.31 mmol, 4 equiv) and *N*-hydroxysuccinimide (36 mg, 0.31 mmol, 4 equiv) were dissolved in DMF (1 mL) and pre-mixed for 5 minutes in a 4 mL vial. The solution was then transferred to the pre-swelled GRGDSGG resin (100 mg, 0.078 mmol) and allowed to agitate for 1 h at room temperature.

Note: As previously reported, care must be taken to avoid addition of base during activation and coupling of (Boc-aminooxy)acetic acid to prevent overacylation.[3] Cleavage from the resin should be done using TFA/EDT/H_2O/TIPS (94:2.5:2.5:1, v/v/v/v). Once cleaved from solid support, aminooxyacetyl-terminated peptides should be manipulated in an acetone-free laboratory to avoid side reactions, and pooling and freezing of HPLC fractions must be done immediately.[4-6]

Test cleavage of resin-bound peptides
A small aliquot of resin (1-5 mg) was washed with CH_2Cl_2 to remove traces of DMF, drained, and treated with a freshly made solution of TFA/H_2O/TIPS (95:2.5:2.5, v/v/v, 0.2 mL) for 2 h at room temperature in a 1 mL disposable PP fritted cartridge. For the aminooxyacetyl-terminated peptide, a freshly made solution of TFA/EDT/H_2O/TIPS (94:2.5:2.5:1, v/v/v/v, 0.2 mL) was used for the cleavage instead. The cleavage solution was collected by filtering the resin through the PP fritted cartridge. The filtrate was evaporated to dryness and the crude peptide was precipitated twice with cold ether (1 mL) followed by decanting. The pellet (crude peptide sample) was dissolved in 1:1 MeCN/H_2O v/v (1 mg/mL) and subjected to LCMS analysis.

Full cleavage of resin-bound peptides
The resin-bound peptide was washed with CH_2Cl_2 to remove traces of DMF, drained, transferred into a 20 mL scintillation glass vial, and treated a freshly made solution of TFA/H_2O/TIPS (95:2.5:2.5, v/v/v, 4 mL). Aminooxy-terminated peptides were treated with a freshly made solution of TFA/EDT/H_2O/TIPS (94:2.5:2.5:1, v/v/v/v, 4 mL). The vial was capped and agitated for 2 hours at room temperature on an orbital shaker. The cleavage mixture was filtered through a disposable, PP fritted cartridge into a 50 mL falcon tube containing cold ether (~30 mL). Following centrifugation using a pulse method for 20 seconds (x 3), the ether was decanted and the peptide pellet was suspended in 1:1 MeCN/H_2O v/v. The sample was frozen and lyophilized.

Analysis and purification of peptides 2a-j, 3a-d, and aminooxyacetyl-GRGDSGG
Crude peptides **2a-j, 3a-d,** and aminooxyacetyl-GRGDSGG **9** were analyzed and characterized by LCMS as described in the General Methods. Following gradient optimization, the peptides were purified by preparative HPLC as described in the General Methods. Peptide **2a-b** were purified using a gradient of 10-70% CH_3CN (0.1% TFA) in water (0.1% TFA). Peptides **2e-g, j** and **3a-b** were purified using a gradient of 20-80% CH_3CN (0.1% TFA) in water (0.1% TFA). Peptides **2c-d** were purified using a gradient of 25-80% CH_3CN (0.1% TFA) in water (0.1% TFA). Peptides **2h** and **3c** were purified using a gradient of 30-80% CH_3CN (0.1% TFA) in water (0.1% TFA). Peptides **2i** was purified using a gradient of 40-80% CH_3CN (0.1% TFA) in water (0.1% TFA). Aminooxyacetyl-GRGDSGG was purified using a gradient of 1-10% CH_3CN (0.1% TFA) in water (0.1% TFA). HPLC fractions containing the desired peptide product were pooled, frozen

immediately and lyophilized. Characterization data for all peptides can be found in Table S1 below. LCMS chromatograms can be found starting on p.S13.

Table S1. Characterization data for *N*-aryl amino acid-terminated peptides and aminooxyacetyl-GRGDSGG.

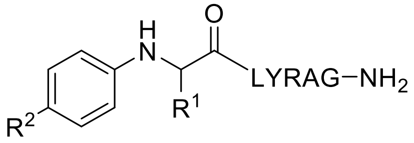

2a-j , 3a-d

Peptide	crude purity (%)[a]	purity (%)[b]	yield (%)[c]	*m/z* [M + H]$^+$ (cald)	*m/z* [M + H]$^+$ (obsd)	Retention time (min)
**2a:** R^1= H, R^2 = NMe_2	74	>99	20	754.4359	754.4350	7.74
**2b:** R^1= Me, R^2 = NMe_2	89	>99	52	768.4515	768.4497	7.76
**2c:** R^1= Ph, R^2 = NMe_2	95	96	42	415.7372[f]	415.7356[f]	8.65/8.84
**2d:** R^1= CH_2Ph, R^2 = NMe_2	96	>99	29	422.7451[f]	422.7434[f]	8.99
**2e:** R^1= CH_2CH_3, R^2 = NMe_2	56	>99	37	782.4599	782.7	8.09
**2f:** R^1= $(CH_2)_2CH_3$, R^2 = NMe_2	74	>99	30	796.4828	796.4804	8.582
**2g:** R^1= $(CH_2)_3CH_3$, R^2 = NMe_2	74	>99	31	405.7529[f]	405.7514[f]	9.038
**2h:** R^1= $(CH_2)_5CH_3$, R^2 = NMe_2	79	>99	20	419.7685[f]	419.7668[f]	9.962
**2i:** R^1= $(CH_2)_9CH_3$, R^2 = NMe_2	72	>99	22	447.7998[f]	447.7987[f]	11.744
**2j:** R^1= $CH(CH_3)_2$, R^2 = NMe_2	65	>99	35	796.4828	796.4804	8.54
**3a:** R^1= H, R^2 = OMe	65	99	41	763.3862[e]	763.3798[e]	8.78
**3b:** R^1= Me, R^2 = OMe	54	>99	32	755.4199	755.4188	8.49/8.78
**3c:** R^1= Ph, R^2 = OMe	92	>99	48	409.2214[f]	409.2211[f]	9.92/10.12
**3d:** R^1= CH_2Ph, R^2 = OMe	92	99	28	416.2292[f]	416.2289[f]	10.06/10.21
Aminooxyacetyl-GRGDSGG	84	>99	26	677.2961	677.2955	4.11[d]

[a]Unless otherwise noted, analytical LCMS analyses were performed on a 5 μM, 150 x 4.6 mm C18 Vydac column from Mac-Mod Analytical, Inc (cat# 218TP5415) with a flow rate of 0.5 mL/min using a 12 min 5-95% linear gradient of MeCN (0.1% TFA) in water (0.1% TFA). The purity at 214 nm wavelength is reported in all cases. [b]RP-HPLC purity at 214 nm after purification. [c]Yields after purification by RP-HPLC are based on resin loading. [d]LCMS analysis was performed using a 12 min 1-10% linear gradient of MeCN (0.1% TFA) in water (0.1% TFA). [e] [M + Na]$^+$ detected. [f] [M + 2H]$^+$ detected.

Oxime ligation reactions procedures

General: All of the ligation studies were performed based on optimized reaction conditions previously reported.[1] Fresh stock solutions were prepared in all cases and used immediately in the ligation reactions. The reagents were combined in a 4 mL glass vial with a small stir bar. Vials were either left uncapped exposed to air, or the solution was sparged with O_2 and left under a balloon filled with O_2. Small aliquots (100 μL) were periodically analyzed by LCMS to monitor the progress of the reaction. % Oxime conversions were calculated using areas obtained from peaks on LCMS traces at 214 nm, where % oxime conversion = [(area of oxime **4a-i**)/(area of **2a-i** + **4a-i**)] x 100.

Purification of ketoximes

Following the ligation reactions, ketoxime peptides **4b-d** and **17-19** (2 mL, 1 mM ketoximes in 100 mM phosphate buffer) were diluted 2-fold with 1: 1 CH_3CN (0.1% TFA) and water (0.1% TFA) prior to purification by preparative HPLC using a gradient of 30-90% CH_3CN (0.1% TFA) in water (0.1% TFA). The run was stopped once the product eluted. HPLC fractions containing the desired product were pooled, frozen immediately, and lyophilized.

Representative protocols for the oxime ligation reactions

A. Oxime ligation conditions for *N*-aryl peptides at pH 7.

The following procedure was used to generate the data presented in Table 1, entries 1-8 and Figure 1 of the manuscript.

The ligation conditions used for these analogs were based on previously optimized procedures for *N*-(*p*-Me_2N-Ph)glycine-LYRAG **2a**.[1] Briefly, a 2 mM stock solution of peptide **2b-i** or **3b-d** and a 10 mM stock solution of *O*-benzylhydroxylamine hydrochloride in 0.1 M potassium phosphate buffer (pH 6.5, 7, 7.5, 8) or 0.1 M ammonium acetate buffer (pH 4.5 and 5.5) were prepared separately. In a 4 mL vial, 1mL of the peptide stock solution (2 mM) was mixed with 1mL of the *O*-benzylhydroxylamine hydrochloride stock solution (10 mM) to give a final concentration of peptide and *O*-benzylhydroxylamine hydrochloride of 1 mM and 5 mM, respectively. The solution was then sparged with oxygen for 20 seconds before sealing the reaction mixture under an oxygen balloon. Samples were tested at different time points by taking aliquots and analyzing them immediately by LCMS without prior quenching. Appearance of *E* and *Z* ketoxime products was observed as two distinct peaks:

Ketoxime **4b**: HPLC (5-95% MeCN, 12 min gradient) retention time = 10.25, 10.85 min; LCMS (ESI) calcd for $C_{36}H_{53}N_{10}O_8$ $[M + H]^+$ 753.4, found m/z 753.6.

Ketoxime **4c**: HPLC (5-95% MeCN, 12 min gradient) retention time = 10.89 min; LCMS (ESI) calcd for $C_{41}H_{55}N_{10}O_8$ $[M + H]^+$ 815.4, found m/z 815.7.

Ketoxime **4d**: HPLC (5-95% MeCN, 12 min gradient) retention time = 11.25, 11.78 min; LCMS (ESI) calcd for $C_{42}H_{57}N_{10}O_8$ $[M + H]^+$ 829.4, found m/z 829.7.

Ketoxime **4e**: HPLC (5-95% MeCN, 12 min gradient) retention time = 10.45, 11.10 min; LCMS (ESI) calcd for $C_{37}H_{55}N_9O_9$ $[M + H]^+$ 767.4, found m/z 767.7.

Ketoxime **4f**: HPLC (5-95% MeCN, 12 min gradient) retention time = 10.86, 11.49 min; LCMS (ESI) calcd for $C_{38}H_{57}N_{10}O_8$ $[M + H]^+$ 781.4, found m/z 781.7.

Ketoxime **4g**: HPLC (5-95% MeCN, 12 min gradient) retention time = 11.26, 11.89 min; LCMS (ESI) calcd for $C_{39}H_{59}N_{10}O_8$ $[M + H]^+$ 795.4, found m/z 795.7.

Ketoxime **4h**: HPLC (5-95% MeCN, 12 min gradient) retention time = 12.13, 12.70 min; LCMS (ESI) calcd for $C_{41}H_{63}N_{10}O_8$ $[M + H]^+$ 823.5, found m/z 823.7.

Ketoxime **4i**: HPLC (5-95% MeCN, 12 min gradient) retention time = 13.87, 14.40 min; LCMS (ESI) calcd for $C_{45}H_{71}N_{10}O_8$ $[M + H]^+$ 879.5, found m/z 879.8.

Ketoxime **4j**: HPLC (5-95% MeCN, 12 min gradient) retention time = 10.81, 11.46 min; LCMS (ESI) calcd for $C_{38}H_{57}N_{10}O_8$ $[M + H]^+$ 781.4, found m/z 781.7.

Note: It is essential that these reactions are not only performed under an oxygen balloon, but that the solution is also sparged with oxygen for ~30 seconds prior to sealing the reaction under an O_2 atmosphere. This appears to be especially critical when the concentration of the alkoxyamine $\geq$ 5 mM.[1]

B. Oxime ligation conditions for *N*-(*p*-MeO-Ph)-amino acid-terminated peptide analogs

The following procedures were used to generate the data presented in Table 1, entries 5-8 of the manuscript.

At pH 4.5:

The ligation conditions used for these analogs were based on previously optimized procedures for *N*-(*p*-MeOPh)glycine-LYRAG **3a**.[1] Briefly, 2 mM stock solutions of peptide (**3b-d**) and *O*-benzylhydroxylamine hydrochloride were prepared separately using 0.1 M ammonium acetate buffer pH 4.5. In a 4 mL vial, 1 mL of the peptide stock solution (2 mM) was mixed with 1 mL of the *O*-benzylhydroxylamine hydrochloride stock solution (2 mM) to give a final concentration of 1 mM for both reagents. The samples were left stirring open to air for up to 24h. Samples were tested at different time points by taking aliquots. Ketoximes **4b-d** were *not detected*.

Using 1 mM potassium ferricyanide:

The ligation conditions used for these analogs were based on previously optimized procedures for *N*-(*p*-MeOPh)glycine-LYRAG **3a**.[1] A 4 mM stock solution of peptide **3b-d**, 20 mM stock solution of *O*-benzylhydroxylamine hydrochloride, and 4 mM stock solution of potassium ferricyanide were prepared separately in 0.1 M potassium phosphate buffer (pH 7). In a 4 mL vial, 0.5 mL of the peptide stock solution (4 mM) was mixed with 0.5 mL of the *O*-benzylhydroxylamine hydrochloride stock solution (20 mM). The resulting solution was diluted further with 0.5 mL of 0.1 M potassium phosphate buffer (pH 7), followed by addition of 0.5 mL of the $K_3[Fe(CN)_6]$ stock solution (4 mM), to give final concentrations of peptide, *O*-benzylhydroxylamine hydrochloride, and $K_3[Fe(CN)_6]$ of 1 mM, 5 mM, and 1 mM, respectively. Samples were tested at different time points by taking aliquots and analyzing them by LCMS, following dilution and quenching. The reactions were quenched with tris(2-carboxyethyl)phosphine hydrochloride (TCEP) prior to LCMS analysis. Specifically, a 50 µL aliquot of the reaction mixture was treated with 50 µL of a 2 mM TCEP stock solution and the sample was vortexed for 20-30 seconds.

C. Control oxime ligation study using peptide 2b and EDTA.

A 4 mM stock solution of peptide **2b**, a 40 mM stock solution of EDTA, and a 20 mM stock solution of *O*-benzylhydroxylamine hydrochloride in 0.1 M potassium phosphate buffer (pH 7) were prepared separately in new vials. In a new 4 mL vial with a new stir bar, 0.5 mL of the peptide stock solution (4 mM) was mixed with 0.5 mL of the EDTA stock solution (40 mM) and 0.5 mL of the *O*-benzylhydroxylamine hydrochloride stock solution (20 mM). The resulting solution was diluted further with 0.5 mL of the corresponding 0.1 M buffer to give a final concentration of peptide, EDTA, and *O*-benzylhydroxylamine hydrochloride of 1 mM, 10 mM, and 5 mM, respectively. The solution was then sparged with oxygen for 10 seconds before removing the exit needle and sealing the reaction mixture under an oxygen balloon. Samples were tested at different time points by taking aliquots and analyzing them immediately by LCMS without prior quenching. Appearance of ketoxime **4b** was observed. See p. S33 for LCMS chromatogram.

D. Two step oxidation/ligation procedure

The following procedure was used to generate the data presented in Scheme 4 of the manuscript.

In a 4 mL vial, 1 mL of a 2 mM stock solution of peptide **2b-d** in 0.1 M potassium phosphate buffer (pH 7) was prepared. The solution was then sparged with oxygen for 20 seconds

before sealing the reaction mixture under an oxygen balloon. After oxidation to the α-ketoamides (**5b-d**) was achieved (~24 h), 1 mL of the *O*-benzylhydroxylamine hydrochloride stock solution (10 mM) was mixed with the peptide to give a final concentration of peptide and *O*-benzylhydroxylamine hydrochloride of 1 mM and 5 mM, respectively. Samples were tested at different time points by taking aliquots and analyzing them immediately by LCMS without prior quenching.

E. Buffer salt effect with peptide analogs 2a-b.

The studies on the effect of buffer salt described in Figure 2b of the manuscript were conducted after noticing a significant decrease in reactivity upon changing from a phosphate buffer to a glycine-NaOH buffer at pH 8.5 (Table S2).

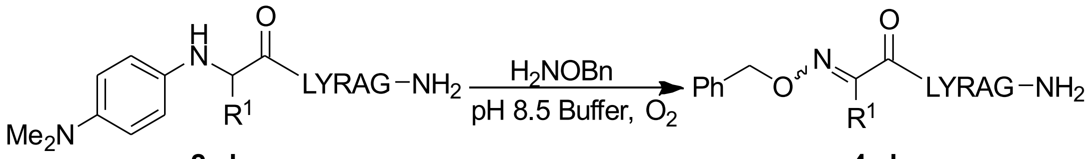

2a-b **4a-b**

a: R^1 = H **b**: R^1 = CH_3

Table S2. Comparison of oxidative coupling conversions in different buffers at pH 8.5.

Peptide	buffer	% oxime at t = 24h
**2a**	Glycine NaOH	8
**2a**	Tris	75
**2a**	Britton-Robinson	92
**2b**	Glycine NaOH	0
**2b**	Tris	88
**2b**	Britton-Robinson	80

Studies described in Figure 2b of the manuscript:

Returning to a of pH 7.5, further studies on buffer salt composition were performed using the same procedure as described in A, using ten different buffers (Tris, TEA, HEPES, TES, PIPES, phosphate, MOPS, Tricine, Bicine, imidazole) with a final buffer concentration of 0.1 M. Premade buffers were purchased from Alfa Aesar through Fisher scientific at concentrations between 0.2-1.0 M and diluted to 0.1 M using ultrapure water from a Synergy UV water purification system (EMD Millipore). The Britton-Robinson "universal buffer" was made using phosphoric acid (85% in water), glacial acetic acid, and o-boric acid and varying amounts of sodium hydroxide following literature procedure to make buffers at pH 4.6, 7 and 8.5.[7]

Control studies were also done at pH 4.5 and pH 7 using the Britton-Robinson "universal buffer" to confirm reactivity despite changing the buffer composition (Table S3).

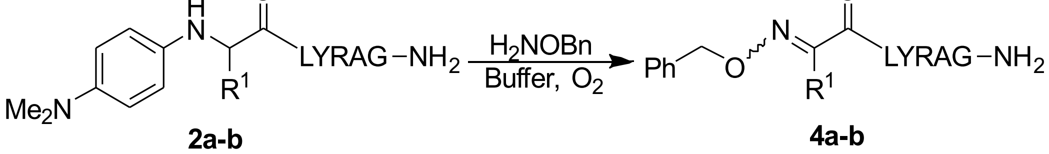

2a-b **4a-b**

a: R^1 = H **b**: R^1 = CH_3

Table S3. Control studies to compare buffer composition while keeping pH constant.

Peptide	buffer	% oxime at t = 24h
**2a**	ammonium acetate, pH 4.5	20
**2a**	Britton-Robinson, pH 4.6	23
**2a**	potassium phosphate, pH 7	96
**2a**	Britton-Robinson, pH 7	96
**2b**	ammonium acetate, pH 4.5	9
**2b**	Britton-Robinson, pH 4.6	1
**2b**	potassium phosphate, pH 7	98
**2b**	Britton-Robinson, pH 7	91

F. Effect of phosphate buffer salt concentration with peptide analogs 2a-b.

To study the effect of buffer salt concentration using potassium phosphate buffer, a stock solution of 100 mM phosphate buffer pH 7 was diluted with ultrapure water to afford final concentration of 50 mM, 25 mM, 10 mM, 5 mM and 1 mM potassium phosphate in water. These solutions were used to monitor oxidative coupling reactions (Table S4).

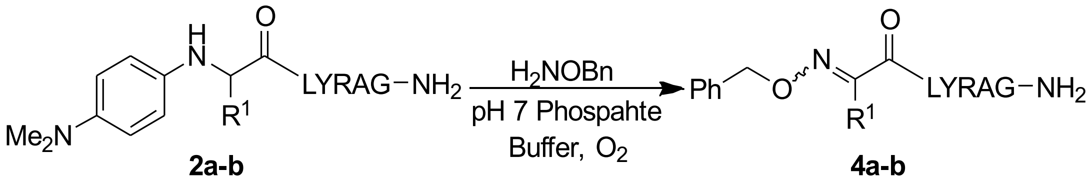

2a-b **4a-b**

a: R^1 = H **b**: R^1 = CH_3

Table S4. Comparative reactivity of **2a-b** with varying potassium phosphate buffer concentration at pH 7.

Peptide	buffer concentration	% oxime at t = 24h
**2a**	1 mM	8
**2a**	5 mM	19
**2a**	10 mM	89
**2a**	25 mM	99
**2a**	50 mM	99
**2b**	1 mM	0
**2b**	5 mM	0
**2b**	10 mM	3
**2b**	25 mM	83
**2b**	50 mM	99

G. Oxime ligation studies with 2a-b, h-i in buffer/EtOH solvent mixtures.

The following procedure was used to generate data presented in Table 2 and Figure 4 of the manuscript.

The studies were performed using the same procedure as described in A, but preparing the 2 mM stock solution of peptide **2a-b, h-j** using a mixture of ethanol and 0.1 M potassium phosphate buffer (pH 7) instead. For 5% EtOH solvent composition, 100 µL EtOH and 900 µL phosphate buffer were combined to make the peptide stock. For 10% EtOH solvent composition, 200 µL EtOH and 800 µL phosphate buffer were combined to prepare the peptide stock solution. For 15% EtOH solvent composition, 300 µL EtOH and 700 µL

phosphate buffer were combined to prepare the peptide stock solution. For 25% EtOH solvent composition, 500 μL EtOH and 500μL phosphate buffer were combined to prepare the peptide stock solution. For 30% EtOH solvent composition, 600 μL EtOH and 400 μL phosphate buffer were combined to prepare the peptide stock solution. For 40% EtOH solvent composition, 800 μL EtOH and 200 μL phosphate buffer were combined to prepare the peptide stock solution. For 50% EtOH solvent composition, 1 mL EtOH was used to prepare the peptide stock solution. Studies were also done with peptide **2b** using MeCN instead of EtOH (Table S5).

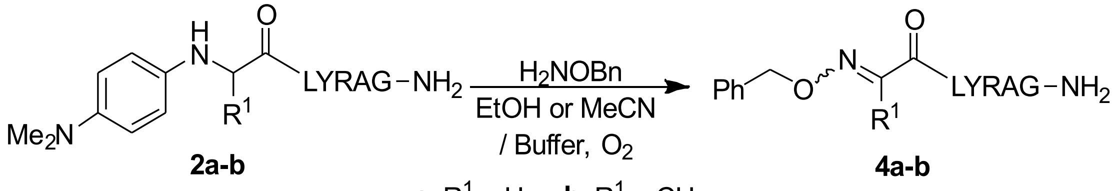

2a-b **4a-b**

a: R^1 = H **b**: R^1 = CH_3

Table S5. Comparative reactivity for peptide **2b** in varying % MeCN *vs* EtOH in phosphate buffer pH 7.

% EtOH	% Oxime t = 24h	% MeCN	% Oxime t = 24h
5	99	5	97
10	96	10	93
15	96	15	87
25	52	25	55
30	34	30	42
40	20	40	39
50	0	50	27

H. Sequence Effects

Table S6. Characterization data for *N*-(*p*-NMe_2-Ph)Ala-**X**-YRAG peptides

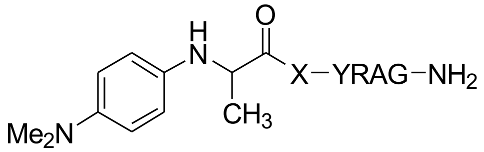

Peptide	crude purity (%)[a]	purity (%)[b]	yield (%)[c]	*m/z* $[M + H]^+$ (cald)	*m/z* $[M + H]^+$ (obsd)	Retention time (min)
X = Trp	88	98	57	841.4468	841.4441	8.08
X = Cys	79	85	43	758.3766	758.3743	7.17
X = Ser	80	>99	55	742.3995	742.3971	6.84
X = Lys	91	>99	65	783.4624	783.4602	6.80
X = Glu	83	>99	59	784.4100	784.4066	6.96
X = Gln	94	96	54	783.4260	783.4236	6.84

[a]Unless otherwise noted, analytical LCMS analyses were performed on a 5 μM, 150 x 4.6 mm C18 Vydac column from Mac-Mod Analytical, Inc (cat# 218TP5415) with a flow rate of 0.5 mL/min using a 12 min 5-95% linear gradient of MeCN (0.1% TFA) in water (0.1% TFA). The purity at 214 nm wavelength is reported in all cases. [b]RP-HPLC purity at 214 nm after purification. [c]Yields after purification by RP-HPLC are based on resin loading. [d]LCMS analysis was performed using a 12 min 1-10% linear gradient of MeCN (0.1% TFA) in water (0.1% TFA).

The studies were performed using the same procedure as described in A.

Ketoxime **10**: HPLC (5-95% MeCN, 12 min gradient) retention time = 10.26, 10.70 min; LCMS (ESI) calcd for $C_{41}H_{52}N_{11}O_8$ $[M + H]^+$ 826.4, found m/z 826.6.

Ketoxime **11**: HPLC (5-95% MeCN, 12 min gradient) retention time = 10.42, 10.70 min; LCMS (ESI) calcd for $C_{33}H_{47}N_{10}O_8S$ $[M + H]^+$ 742.3, found m/z 742.6.

Ketoxime **12**: HPLC (5-95% MeCN, 12 min gradient) retention time = 8.77, 9.36 min; LCMS (ESI) calcd for $C_{33}H_{47}N_{10}O_9$ $[M + H]^+$ 727.3, found m/z 727.6.

Ketoxime **13** HPLC (5-95% MeCN, 12 min gradient) retention time = 8.30, 8.90 min; LCMS (ESI) calcd for $C_{36}H_{54}N_{11}O_8$ $[M + H]^+$ 768.4, found m/z 768.6.

Ketoxime **14**: HPLC (5-95% MeCN, 12 min gradient) retention time = 8.85, 9.46 min; LCMS (ESI) calcd for $C_{35}H_{49}N_{10}O_{10}$ $[M + H]^+$ 769.4, found m/z 769.6.

Ketoxime **15**: HPLC (5-95% MeCN, 12 min gradient) retention time = 8.59, 9.20 min; LCMS (ESI) calcd for $C_{35}H_{50}N_{11}O_9$ $[M + H]^+$ 768.4, found m/z 768.6.

I. Peptide-peptide ligation.

A 2 mM stock solution of peptide **2b-d** and a 20 mM stock solution of aminooxyacetyl-GRGDSGG (**16**) were prepared separately in 0.1 M potassium phosphate buffer (pH 7). In a 4 mL vial, 1 mL of peptide **2b-d** stock solution (2 mM) was mixed with 1 mL of the aminooxyacetyl-GRGDSGG **16** stock solution (20 mM) give final concentrations of 1 mM and 10 mM for peptides **2b-d** and **16**, respectively. The solution was then sparged vigorously with oxygen for 20 seconds, followed by reducing bubbling of O_2 to a minimum flow for 12-24 h. Samples were tested at different time points by taking aliquots and analyzing them by LCMS, following dilution. Samples were analyzed immediately without quenching. Appearance of oxime was observed:

Ketoxime **17**: HPLC (5-95% MeCN, 12 min gradient) retention time = 7.70 min; LCMS (ESI) calcd for $C_{53}H_{84}N_{19}O_{20}$ $[M + H]^+$ 1307.4, found m/z 1307.7.
Ketoxime **18**: HPLC (5-95% MeCN, 12 min gradient) retention time = 8.31 min; LCMS (ESI) calcd for $C_{58}H_{85}N_{19}O_{20}$$[M + H]^+$ 1369.4, found m/z 1369.9.
Ketoxime **19**: HPLC (5-95% MeCN, 12 min gradient) retention time = 8.63 min; LCMS (ESI) calcd for $C_{59}H_{87}N_{19}O_{20}$ $[M + H]^+$ 1382.5 found m/z 1382.9.

*These reactions were left under a flow of oxygen due to the excess of aminooxyacetyl-GRGDSGG **16**. Previous work showed that when using > 5 mM alkoxyamine concentrations, sparging of the solution with O_2 is critical.*[1]

Solution phase synthesis procedures

4-(*p*-dimethylamino-phenyl)glycine *tert*-butyl ester (6)

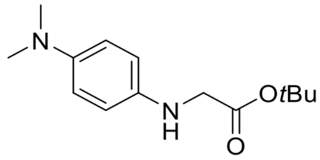

4-Dimethylamino-aniline (1.32 g, 9.69 mmol, 3.78 equiv) and sodium acetate (210.7 mg, 2.57 mmol, 1.00 equiv) were added to an oven-dried 5-mL microwave vial with a stir bar. The vial was capped and flushed with argon. Dry tetrahydrofuran (3.41 mL) and *tert*-butyl bromoacetate (380.0 µL, 2.57 mmol, 1.00 equiv) were added to the vial and the reaction mixture was stirred at room temperature under an argon atmosphere overnight. The crude was transferred to a round-bottom flask, evaporated to dryness, and purified by automated flash-column chromatography eluted with a 15% ethyl acetate-hexanes to 46 % ethyl acetate-hexanes gradient to afford the product as a red oil (462.7 mg, 72% yield). **^{1}H NMR** (600 MHz, $CDCl_3$) δ 6.75 (d, *J* = 6.74 Hz, 2H), δ 6.60 (d, *J* = 6.60 Hz, 2H) δ 3.91 (br s, 1H), δ 3.76 (s, 2H), δ 2.83 (s, 6H), δ 1.49 (s, 9H). **^{13}C NMR** (600 MHz, $CDCl_3$) δ 170.71, 144.45, 139.72, 115.70, 114.56, 81.59, 47.67, 42.13, 28.09. **IR** (neat) 3355, 2975, 2930, 1732, 1514, 1226, 1148. **HRMS** calculated for [$C_{14}H_{22}N_2O_2$]: *m/z* $[M+1]^+$ = 250.1676, observed *m/z* $[M]^+$ = 250.1671. **R_f** = 0.29 (30% ethyl acetate-hexanes).

4-(*p*-dimethylamino-phenyl)alanine *tert*-butyl ester (8)

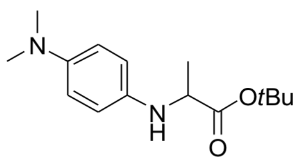

4-Dimethylamino-aniline (600.0 mg, 4.41 mmol, 2.00 equiv) and sodium acetate (180.7 mg, 2.20 mmol, 1.00 equiv) were added to a 10-mL round-bottom flask prior to the addition of ethanol (1.47 mL) and *tert*-butyl 2-bromopropanoate (294.9 µL, 2.20 mmol, 1.00 equiv). The round-bottom flask was capped with a septum and flushed with argon. The solution was allowed to stir under an atmosphere of argon at room temperature overnight. The crude solution was condensed to dryness before the product was isolated by flash-column chromatography eluted with a 10% ethyl acetate-hexanes to 40% ethyl acetate-hexanes gradient. This afforded a brown solid (386.9 mg, 84% yield). **^{1}H NMR** (600 MHz, $CDCl_3$) δ 6.71 (d, J = 6.71 Hz, 2H), δ 6.60 (d, J = 6.60 Hz, 2H) δ 3.94 (q, *J* = 3.93 Hz, 1H), δ 2.82 (s, 6H), δ 1.42 (s, 9H), δ 1.41 (d, *J* = 1.40 Hz, 3 H). **^{13}C NMR** (600 MHz, $CDCl_3$) δ 174.31, 144.58, 139.17, 115.62, 115.39, 81.15, 53.86, 42.15, 28.04, 19.12. **IR** (neat) 3385, 2973, 2907, 2787, 1704, 1518, 1159, 1050, 1035. **HRMS** calculated for [$C_{15}H_{24}N_2O_2$] *m/z* $[M+1]^+$ = 264.1832, observed *m/z* $[M]^+$ = 264.1835. **R_f** = 0.28 (20% ethyl acetate-hexanes). **MP** = 51.6 ºC – 53.8 ºC.

General procedures used to generate the data in Table S7:

tert-butyl 2-((4-(dimethylamino)phenyl)amino)-2-oxoacetate (7)

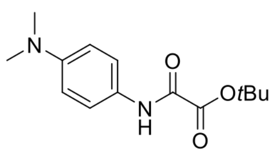

4-(*p*-dimethylamino-phenyl)glycine *tert*-butyl ester **7** (25.0 mg, 0.10 mmol, 1.00 equiv) was added to a 4-mL vial with a stir bar. Solvent (1.00 mL) was added to the vial and the solution was sparged with oxygen for 30 seconds. The solution was allowed to stir under an oxygen atmosphere for 24 hours, unless otherwise noted. The crude solution was condensed to dryness before the product was isolated by flash-column chromatography (eluted with 5% ethyl acetate-hexanes to 40% ethyl acetates-hexanes gradient) affording a yellow oil (11.1 mg, 45 % yield). **^{1}H NMR** (600 MHz, $CDCl_3$) δ 7.87 (s, 1H), δ 7.37 (d, J = 7.37 Hz, 2H), δ 6.68 (d, J = 6.68 Hz, 2H), δ 3.01 (s, 6H), δ 1.58 (s, 9H). **^{13}C NMR** (600 MHz, $CDCl_3$) δ 163.32, 151.27, 145.74, 137.11, 124.15, 112.10, 82.16, 40.44, 28.18. **IR** (neat) 3384, 2973, 2932, 2787, 1704, 1518, 1159, 1050, 1035. **HRMS** calculated for [$C_{14}H_{20}N_2O_3$] calculated *m/z* $[M + H]^+$ = 265.1547, observed *m/z* $[M + H]^+$ = 265.1544. **R_f** = 0.34 (20% ethyl-acetate hexanes).

The following is numerical data that pertains to Scheme 5 in the manuscript. The minor peaks that indicate further oxidation are unidentified at this time.

Table S7. *N*-(*p*-NMe_2-Ph)glycine *tert*-butyl ester oxidation in organic solvents

Entry	Solvent	% conversion to **7**[a]
1	MeCN: DCE (5:1)	50 (58[b])
2	EtOH	43 (51[b])
3	Dry THF	80 (85[b])
4	DCM	90 (91[b])

[a] The percentages were determined using NMR and are an average of three trials. [b] % oxidation including unidentified by-products

3. LCMS Chromatograms

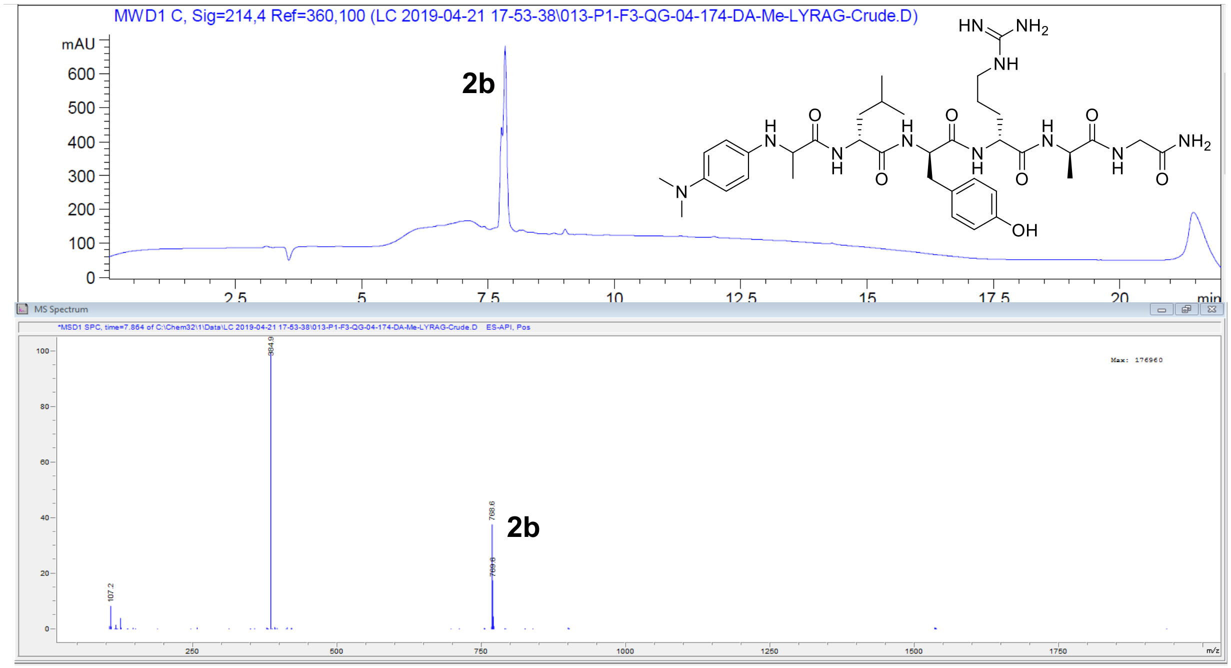

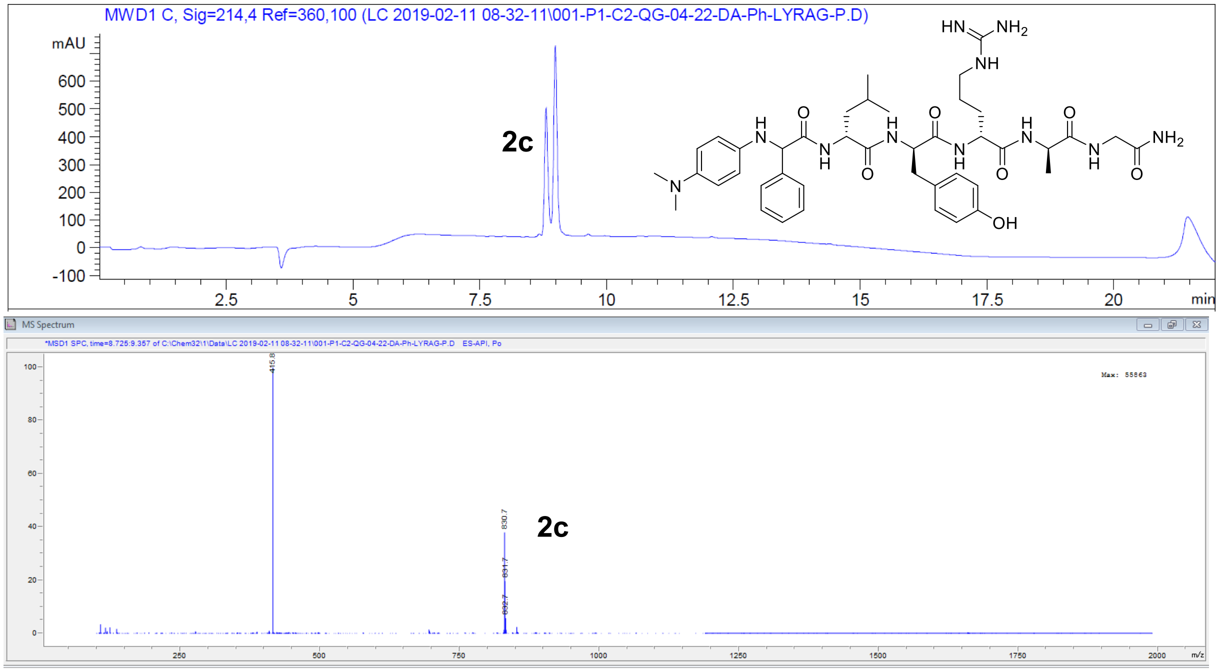

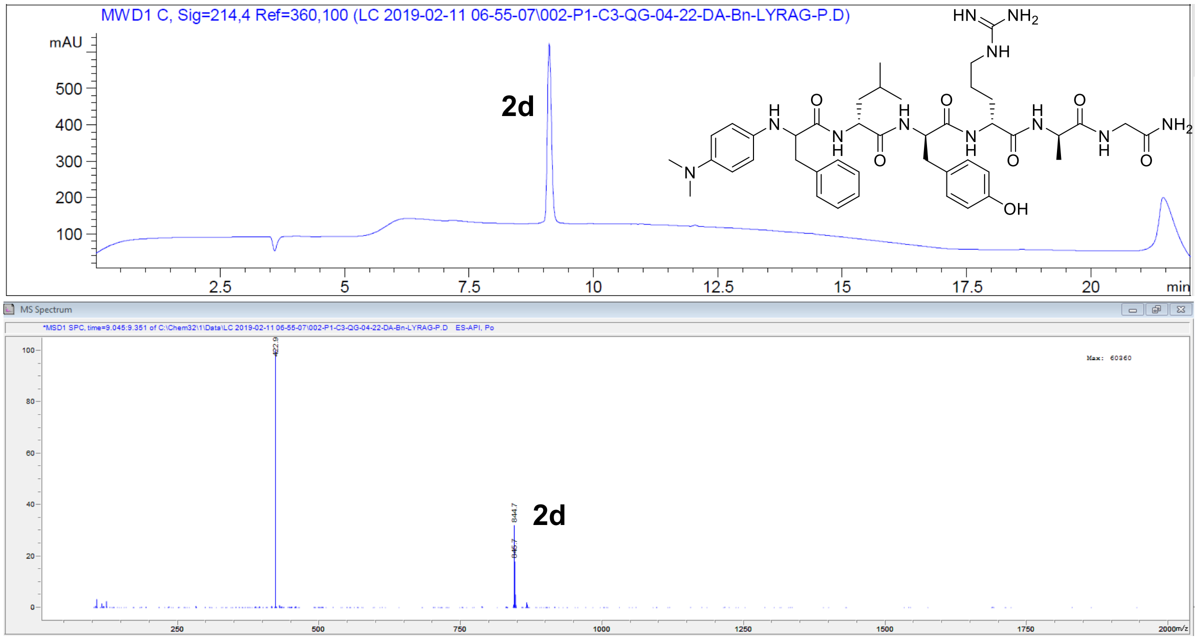
MWD1 C, Sig=214,4 Ref=360,100 (LC 2019-02-11 06-55-07\002-P1-C3-QG-04-22-DA-Bn-LYRAG-P.D)
mAU
2d
min
MS Spectrum
2d

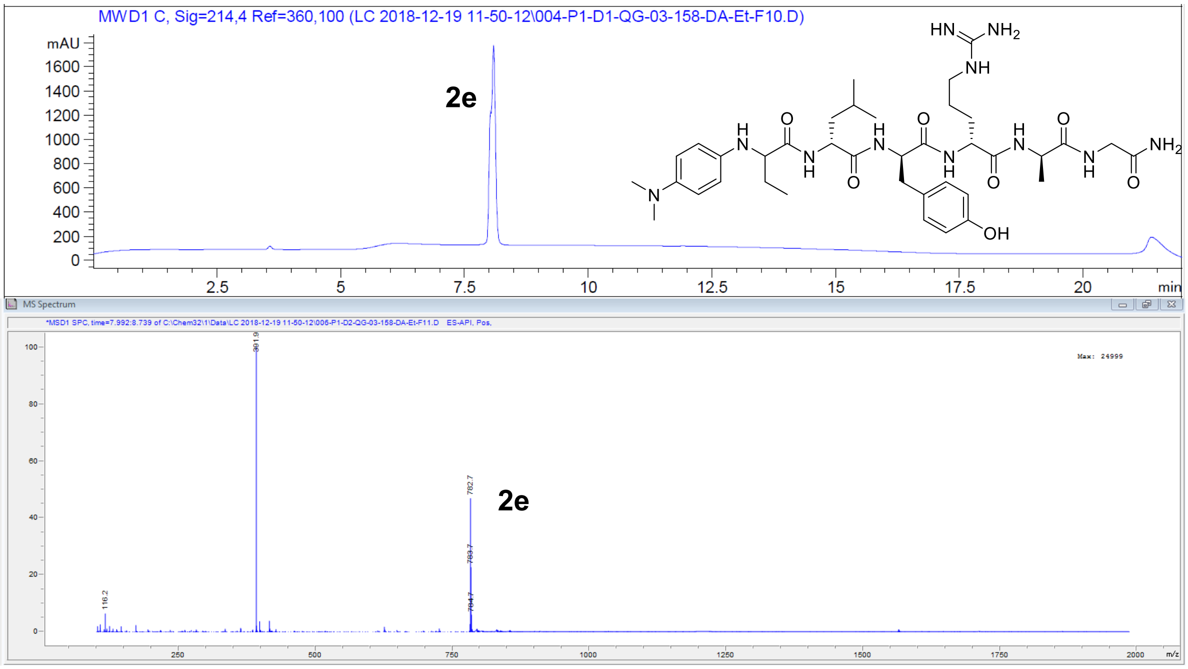
MWD1 C, Sig=214,4 Ref=360,100 (LC 2018-12-19 11-50-12\004-P1-D1-QG-03-158-DA-Et-F10.D)
mAU
2e
min
MS Spectrum
2e

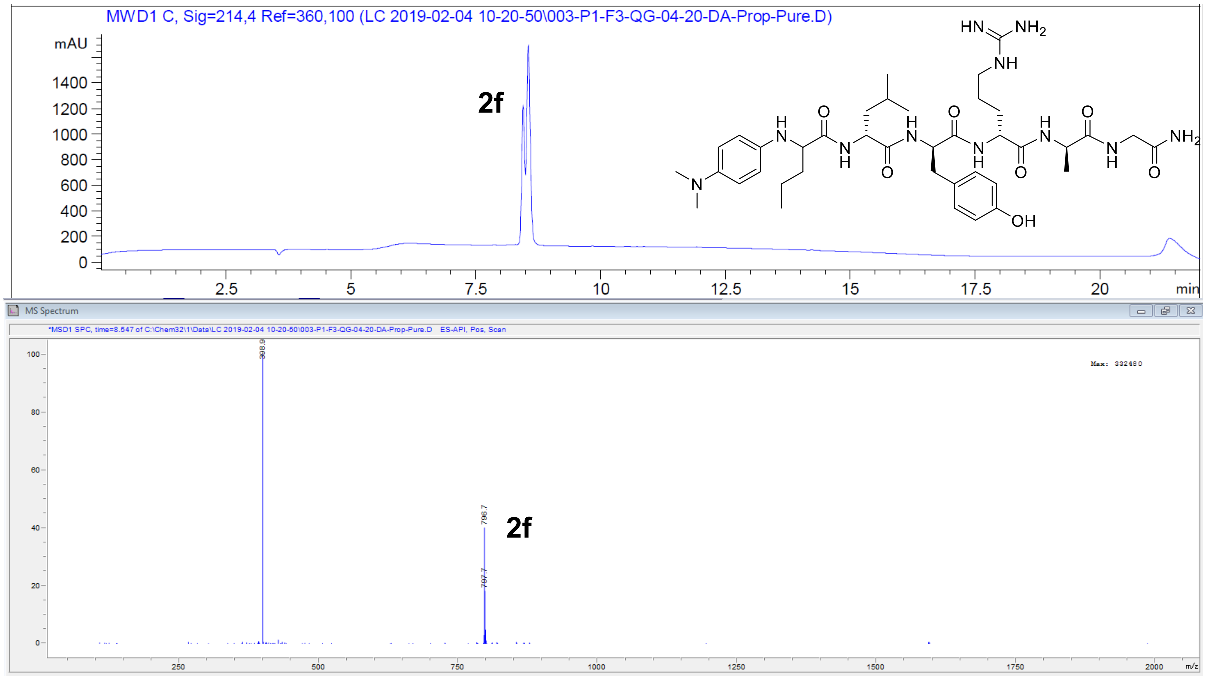
MWD1 C, Sig=214,4 Ref=360,100 (LC 2019-02-04 10-20-50\003-P1-F3-QG-04-20-DA-Prop-Pure.D)
mAU
2f
HN
NH2
NH
H
N
O
OH
NH2
min
MS Spectrum
398.9
796.7
797.7
2f
m/z

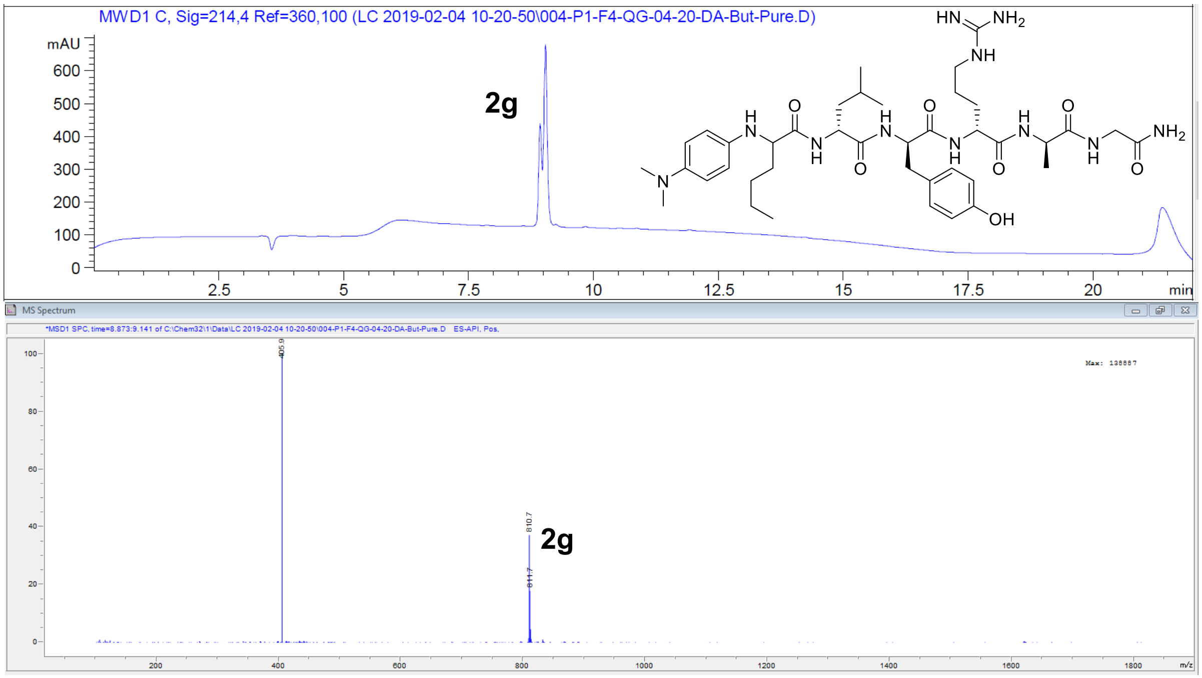
MWD1 C, Sig=214,4 Ref=360,100 (LC 2019-02-04 10-20-50\004-P1-F4-QG-04-20-DA-But-Pure.D)
mAU
2g
HN
NH2
NH
H
N
O
OH
NH2
min
MS Spectrum
405.9
810.7
811.7
2g
m/z

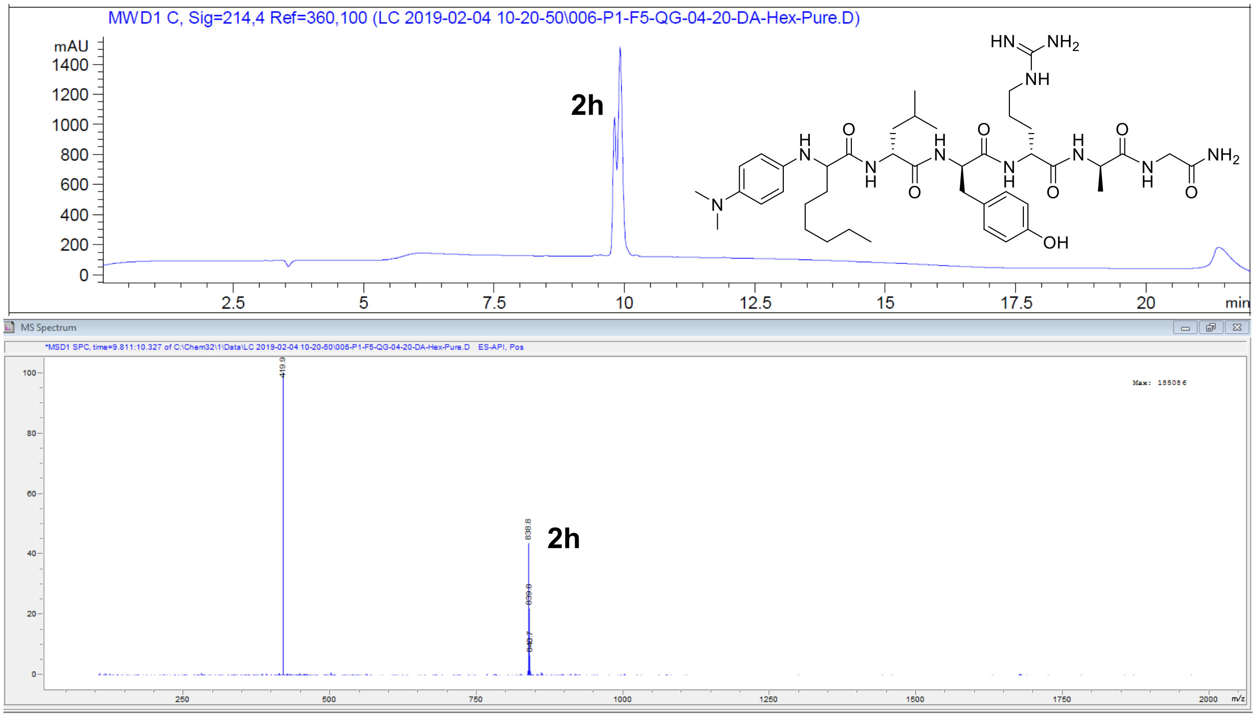
MWD1 C, Sig=214,4 Ref=360,100 (LC 2019-02-04 10-20-50\006-P1-F5-QG-04-20-DA-Hex-Pure.D)
mAU
1400
1200
1000
800
600
400
200
0
2.5
5
7.5
10
12.5
15
17.5
20
min
2h
HN
NH2
NH
H
N
O
OH
MS Spectrum
100
80
60
40
20
0
419.9
838.8
2h
250
500
750
1000
1250
1500
1750
2000
m/z

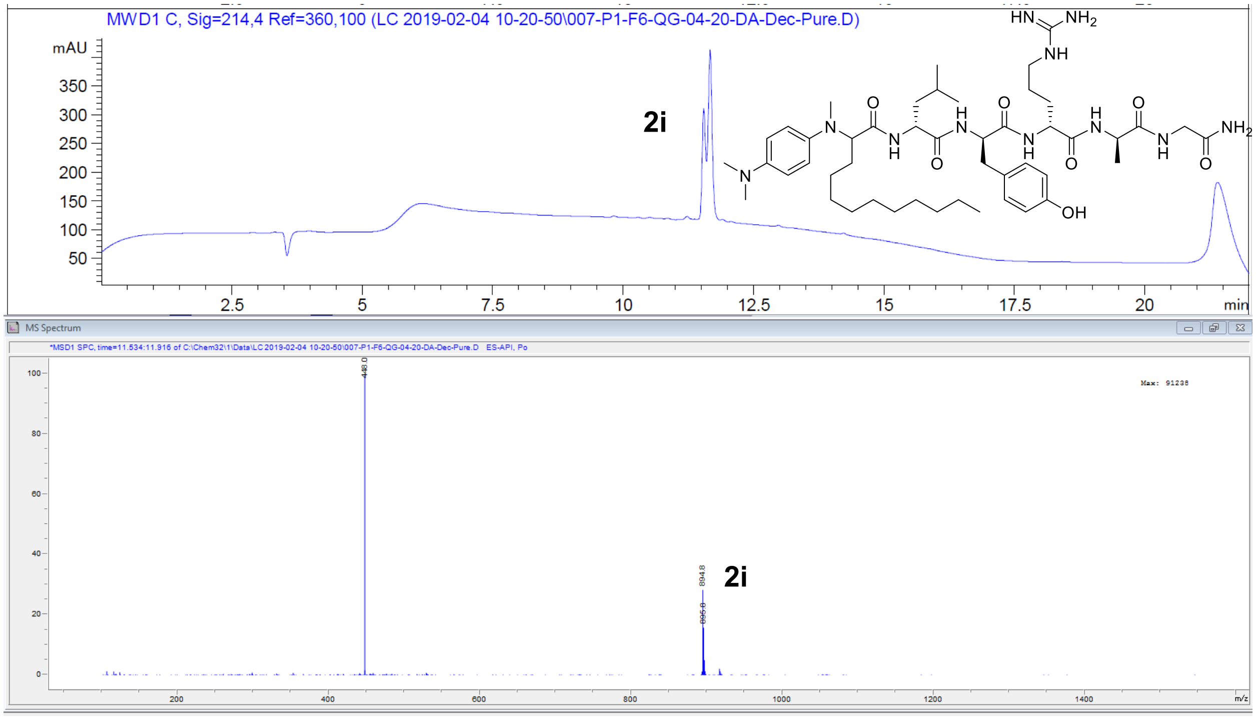
MWD1 C, Sig=214,4 Ref=360,100 (LC 2019-02-04 10-20-50\007-P1-F6-QG-04-20-DA-Dec-Pure.D)
mAU
350
300
250
200
150
100
50
2.5
5
7.5
10
12.5
15
17.5
20
min
2i
HN
NH2
NH
N
H
O
OH
MS Spectrum
100
80
60
40
20
0
448.0
894.8
2i
200
400
600
800
1000
1200
1400
m/z

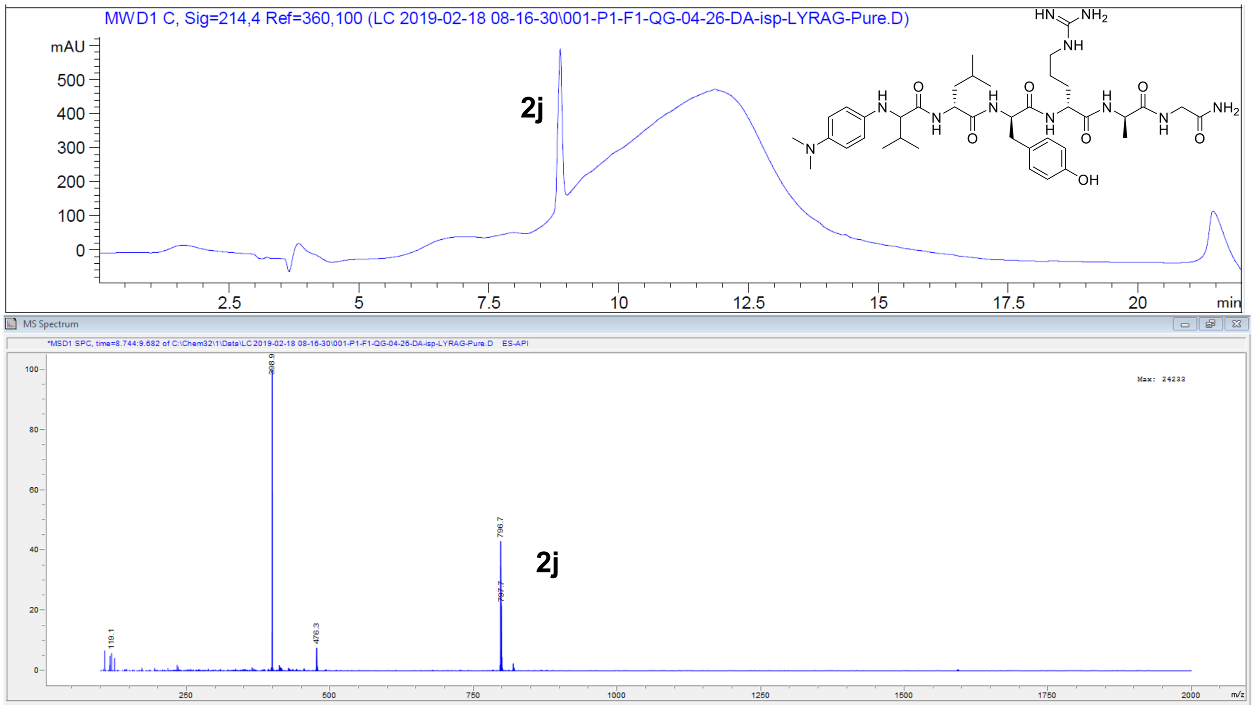
MWD1 C, Sig=214,4 Ref=360,100 (LC 2019-02-18 08-16-30\001-P1-F1-QG-04-26-DA-isp-LYRAG-Pure.D)
mAU
min
2j
MS Spectrum
796.7
476.3
119.1
2j
m/z

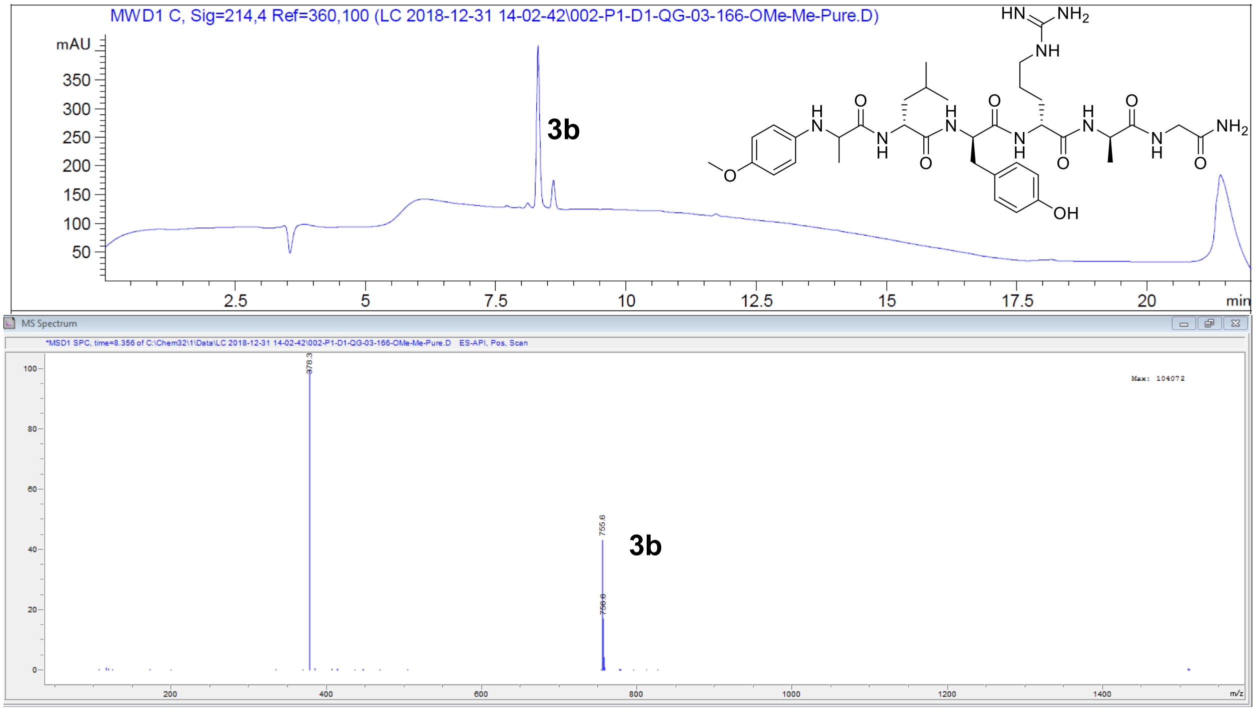
MWD1 C, Sig=214,4 Ref=360,100 (LC 2018-12-31 14-02-42\002-P1-D1-QG-03-166-OMe-Me-Pure.D)
mAU
min
3b
MS Spectrum
378.3
755.6
3b
m/z

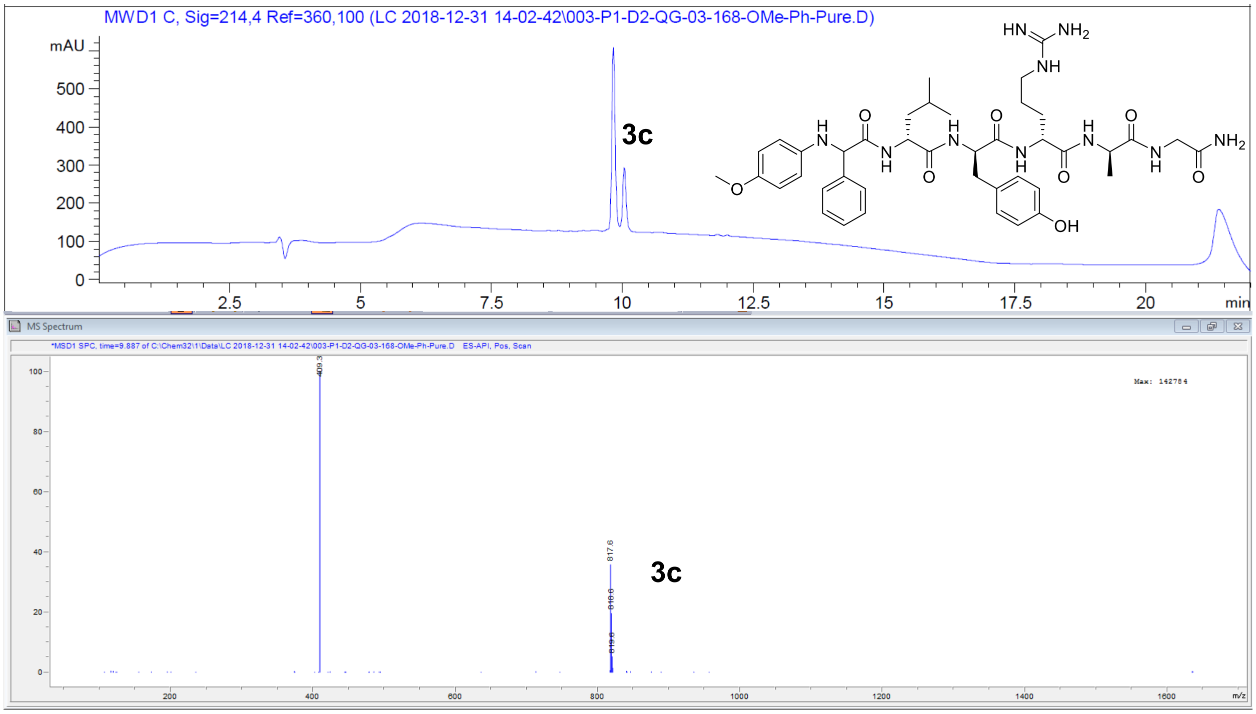
MWD1 C, Sig=214,4 Ref=360,100 (LC 2018-12-31 14-02-42\003-P1-D2-QG-03-168-OMe-Ph-Pure.D)
mAU
min
3c
MS Spectrum
3c

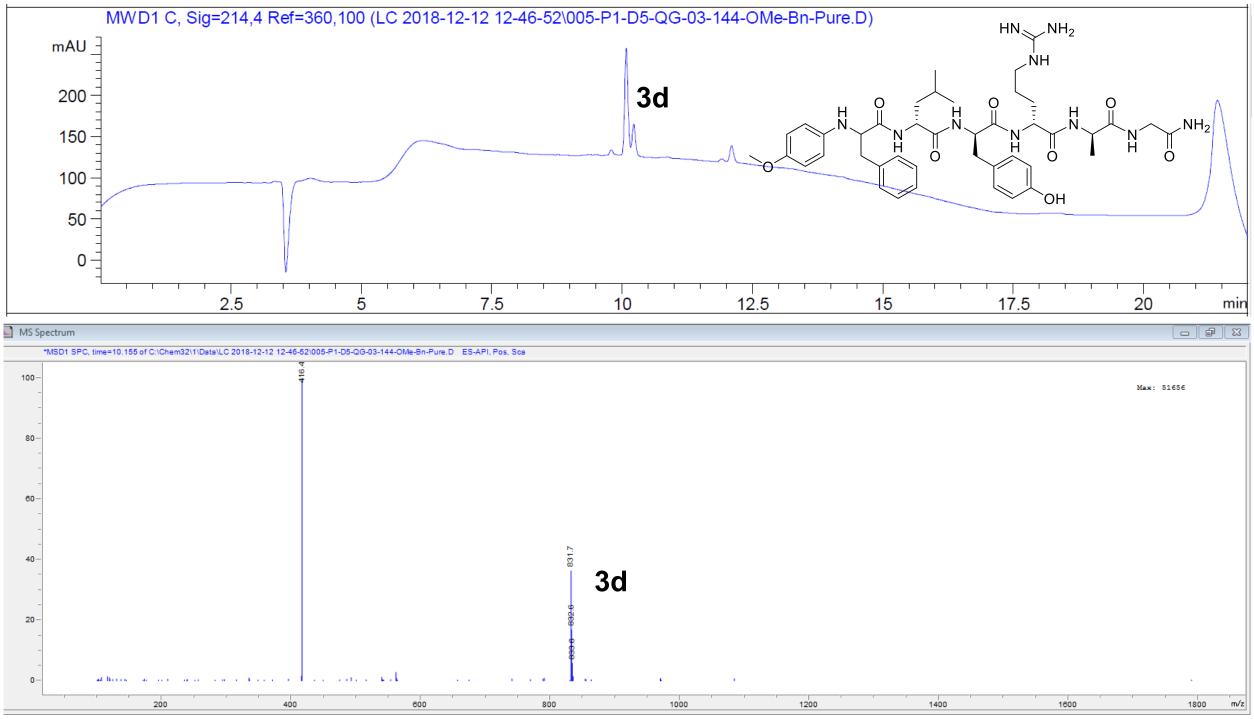
MWD1 C, Sig=214,4 Ref=360,100 (LC 2018-12-12 12-46-52\005-P1-D5-QG-03-144-OMe-Bn-Pure.D)
mAU
min
3d
MS Spectrum
3d

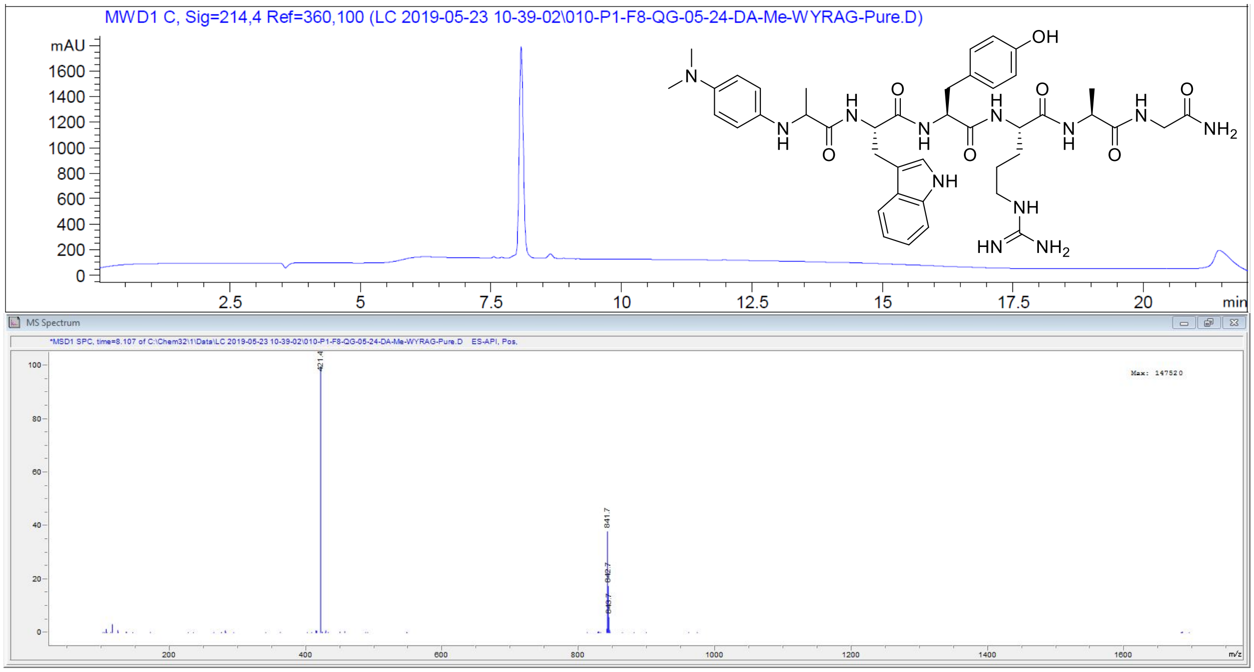
MWD1 C, Sig=214,4 Ref=360,100 (LC 2019-05-23 10-39-02\010-P1-F8-QG-05-24-DA-Me-WYRAG-Pure.D)
mAU
min
MS Spectrum
421.4
841.7
m/z

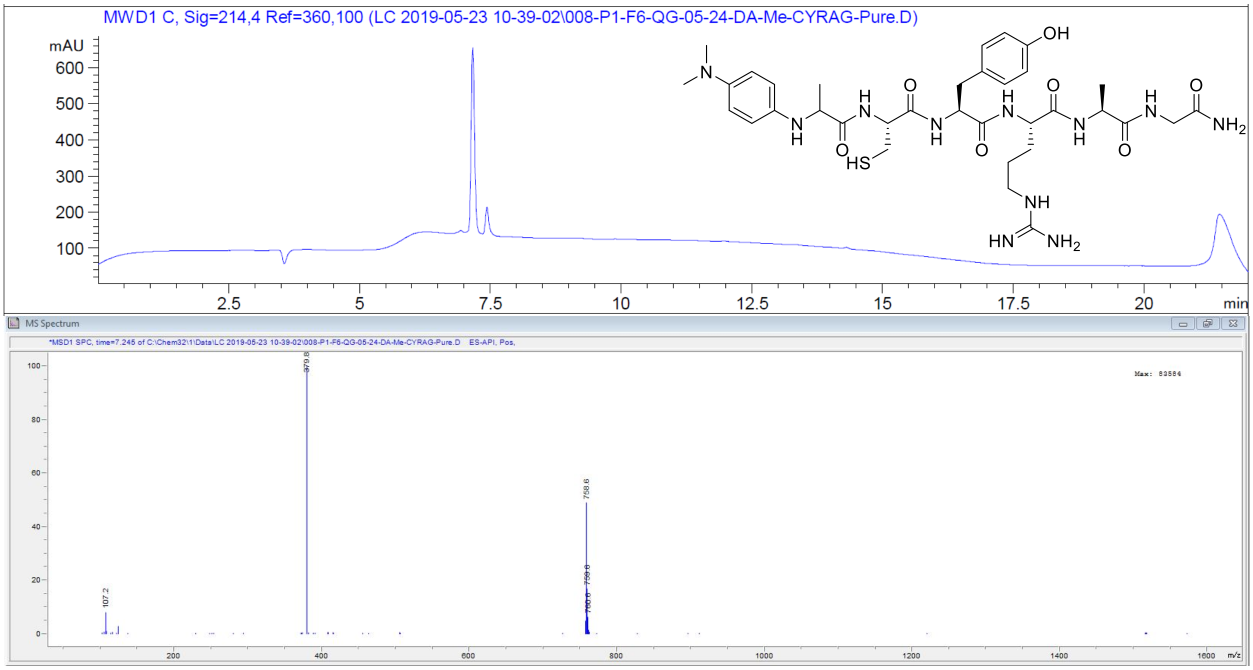
MWD1 C, Sig=214,4 Ref=360,100 (LC 2019-05-23 10-39-02\008-P1-F6-QG-05-24-DA-Me-CYRAG-Pure.D)
mAU
min
MS Spectrum
107.2
379.8
758.6
m/z

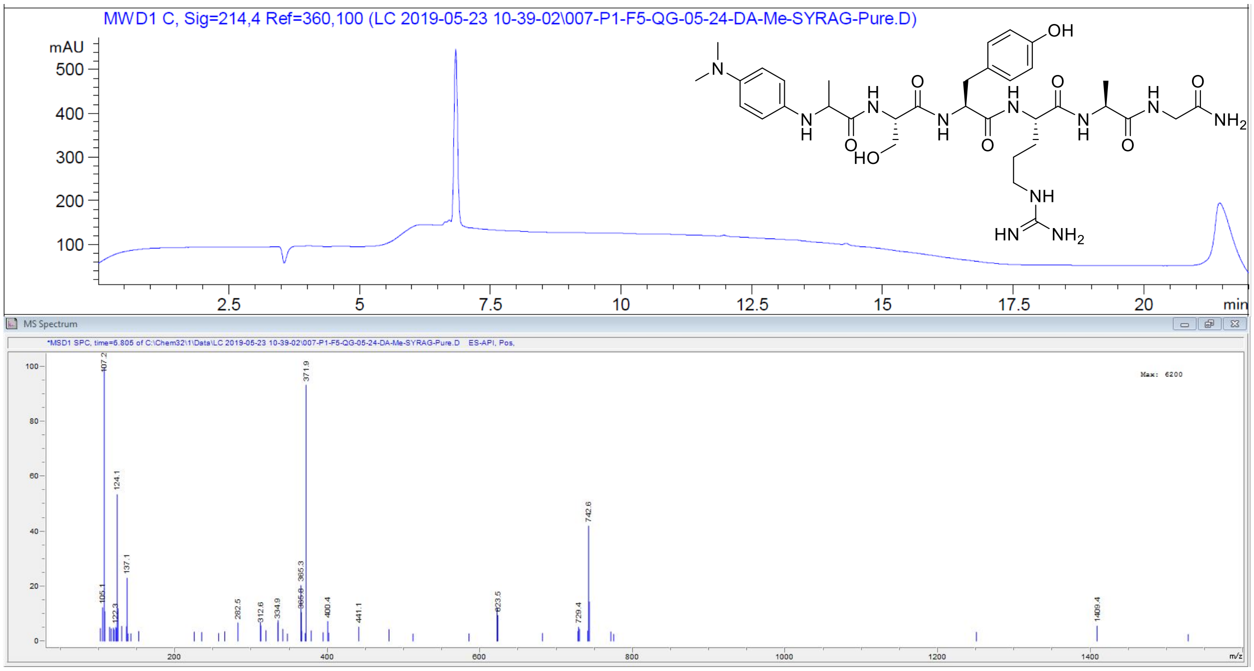
MWD1 C, Sig=214,4 Ref=360,100 (LC 2019-05-23 10-39-02\007-P1-F5-QG-05-24-DA-Me-SYRAG-Pure.D)
mAU
min
MS Spectrum
Max: 6200
107.2
124.1
137.1
105.1
122.3
282.5
312.6
334.9
365.3
371.9
400.4
441.1
623.5
729.4
742.6
1409.4
m/z
OH
N
H
O
HO
NH
HN
NH2

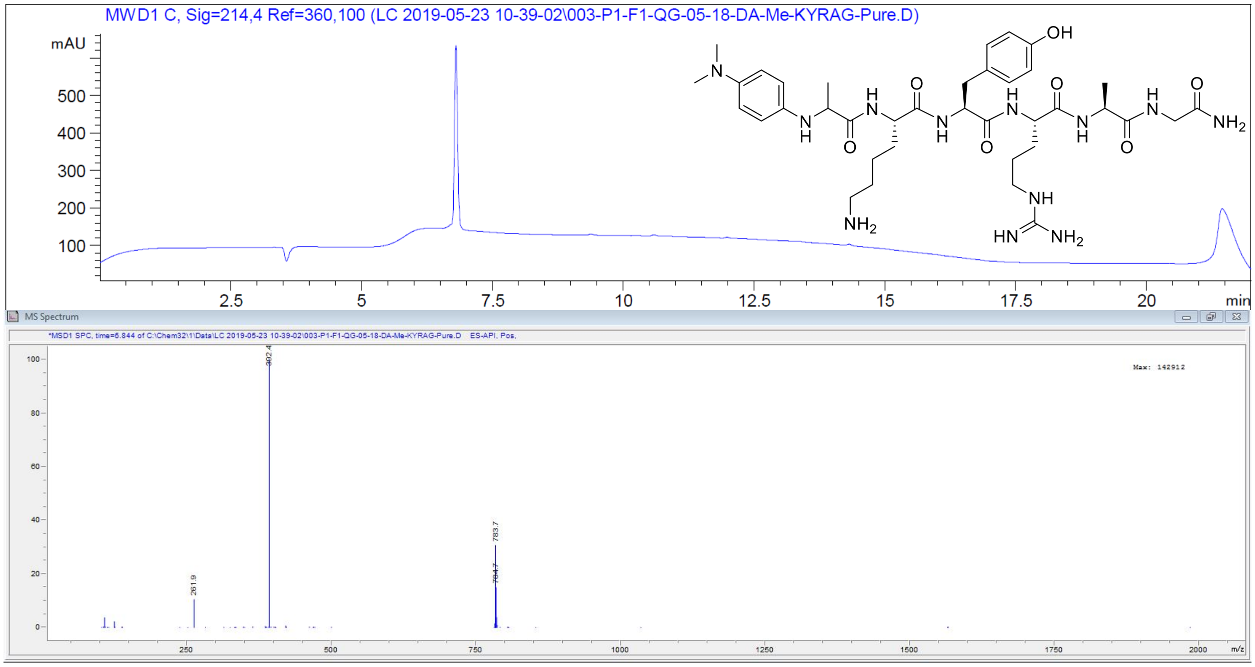
MWD1 C, Sig=214,4 Ref=360,100 (LC 2019-05-23 10-39-02\003-P1-F1-QG-05-18-DA-Me-KYRAG-Pure.D)
mAU
min
MS Spectrum
Max: 162912
261.9
392.4
783.7
784.7
m/z
OH
N
H
O
NH2
NH
HN
NH2

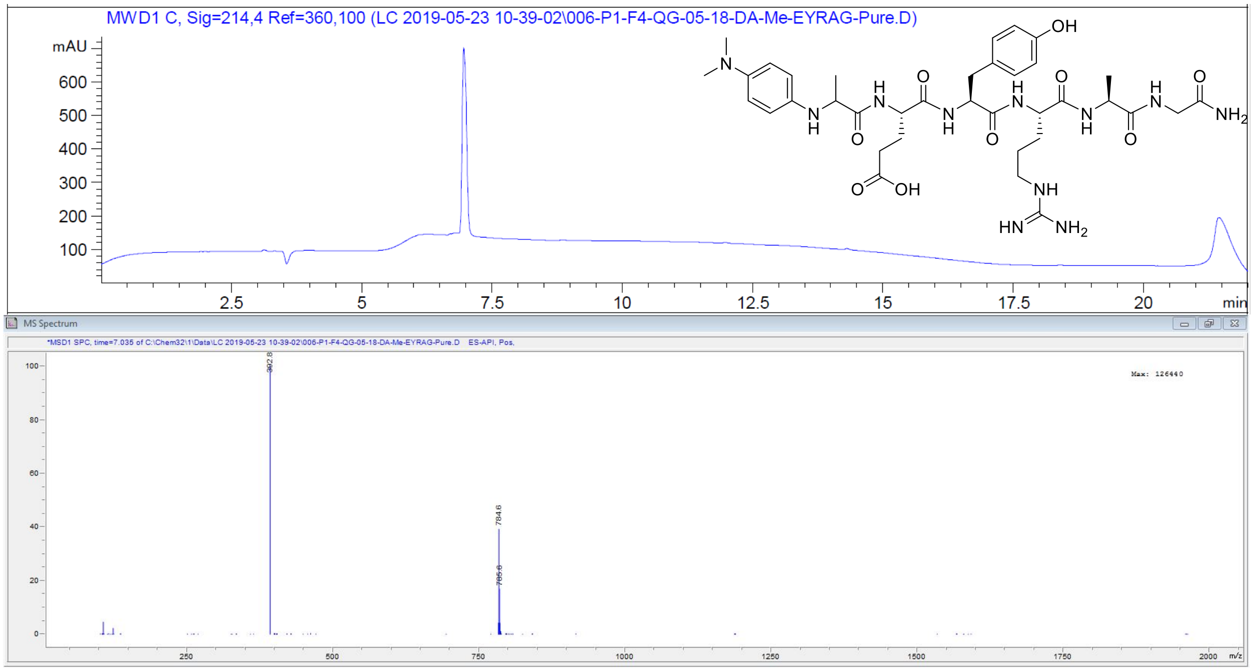
MWD1 C, Sig=214,4 Ref=360,100 (LC 2019-05-23 10-39-02\006-P1-F4-QG-05-18-DA-Me-EYRAG-Pure.D)
mAU
600
500
400
300
200
100
2.5
5
7.5
10
12.5
15
17.5
20
min
MS Spectrum
100
80
60
40
20
0
250
500
750
1000
1250
1500
1750
2000
m/z

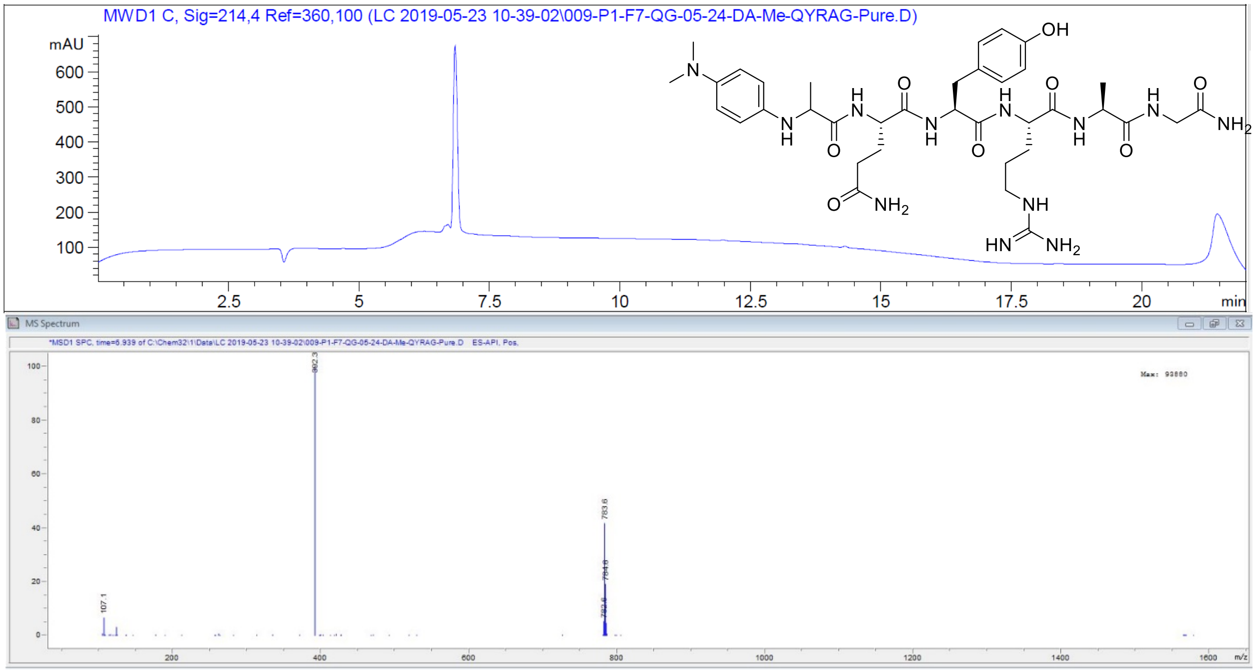
MWD1 C, Sig=214,4 Ref=360,100 (LC 2019-05-23 10-39-02\009-P1-F7-QG-05-24-DA-Me-QYRAG-Pure.D)
mAU
600
500
400
300
200
100
2.5
5
7.5
10
12.5
15
17.5
20
min
MS Spectrum
100
80
60
40
20
0
200
400
600
800
1000
1200
1400
1600
m/z

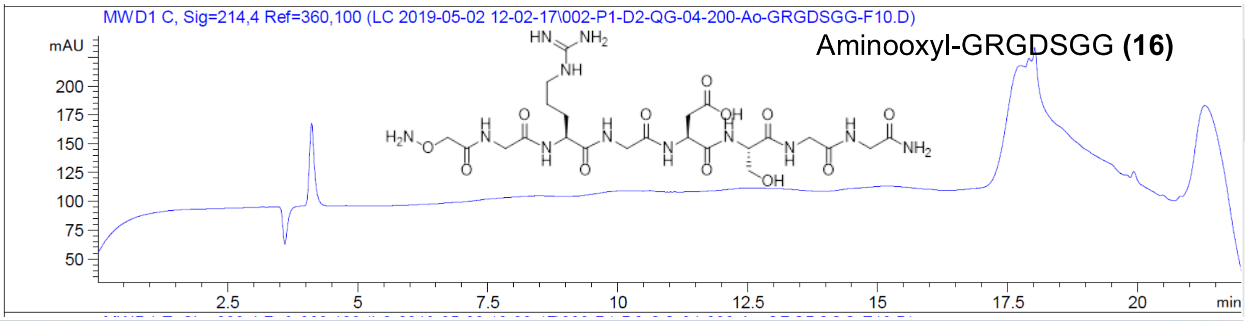
MWD1 C, Sig=214,4 Ref=360,100 (LC 2019-05-02 12-02-17\002-P1-D2-QG-04-200-Ao-GRGDSGG-F10.D)
Aminooxyl-GRGDSGG **(16)**
mAU
200
175
150
125
100
75
50
2.5
5
7.5
10
12.5
15
17.5
20
min

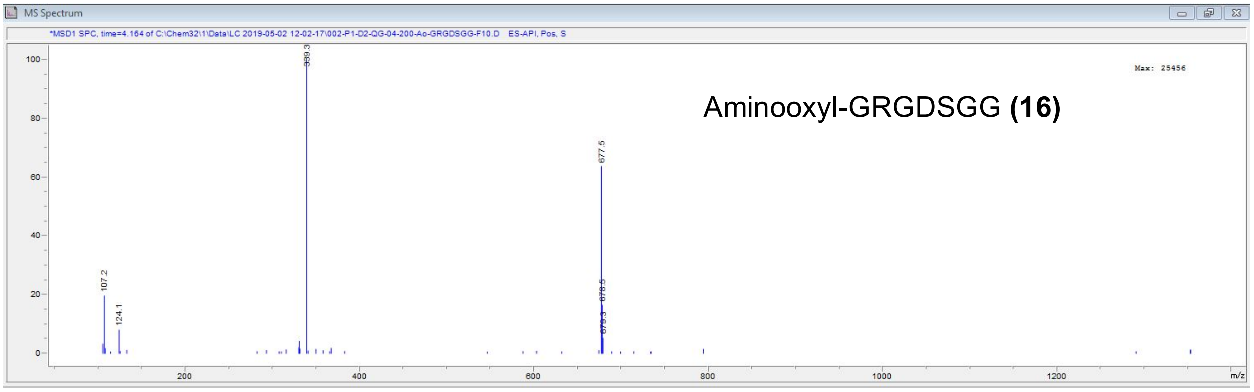
MS Spectrum
*MSD1 SPC, time=4.164 of C:\Chem32\1\Data\LC 2019-05-02 12-02-17\002-P1-D2-QG-04-200-Ao-GRGDSGG-F10.D ES-API, Pos, S
Max: 25456
Aminooxyl-GRGDSGG **(16)**
339.3
677.5
107.2
124.1
200
400
600
800
1000
1200
m/z

Oxime ligation reactions:

Ligation study of **2b** and *O*-benzylhydroxylamine hydrochloride (1:5 ratio) at **pH 7** under O_2 atmosphere:

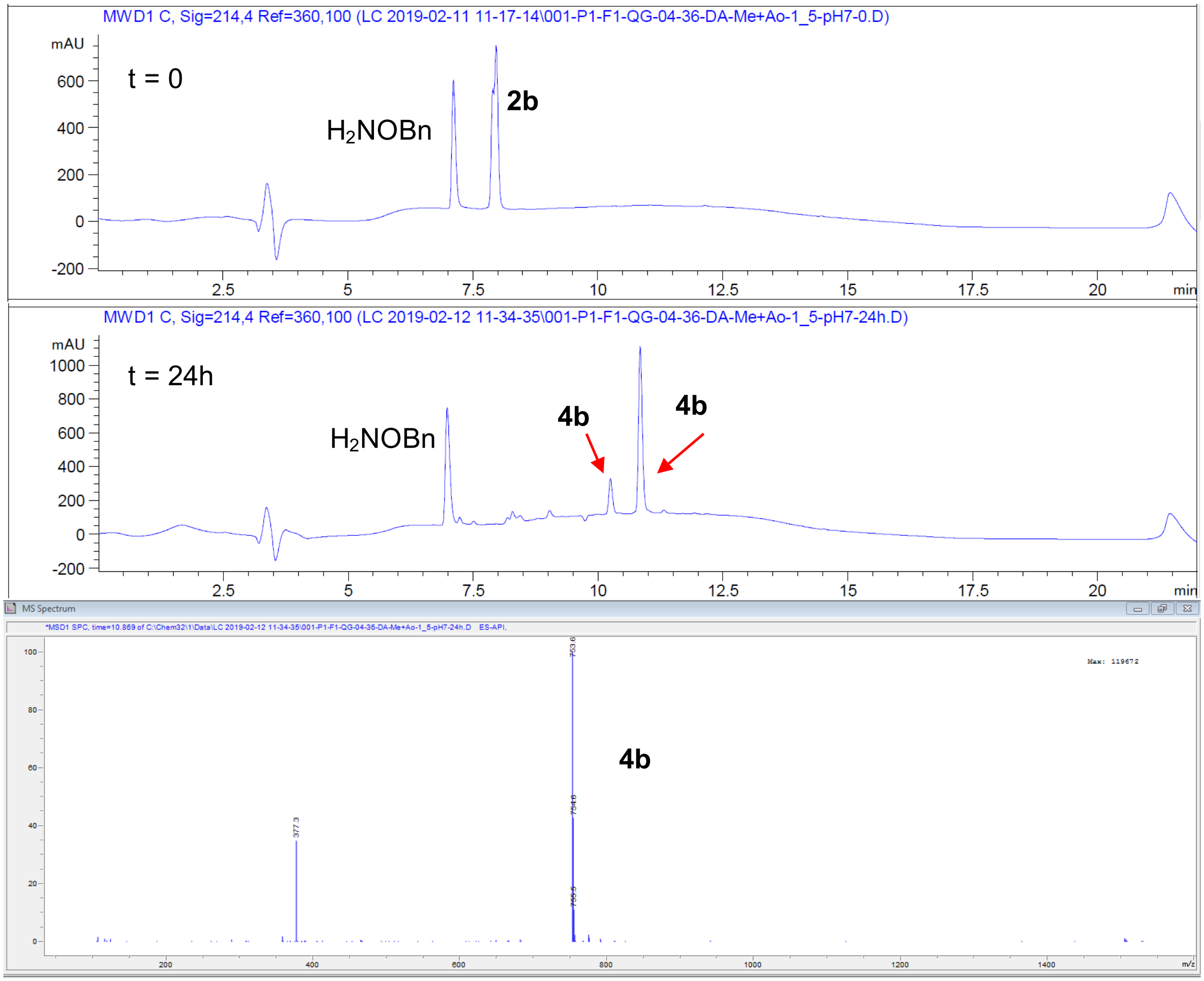

Ligation study of **2c** and *O*-benzylhydroxylamine hydrochloride (1:5 ratio) at **pH 7** under O_2 atmosphere:

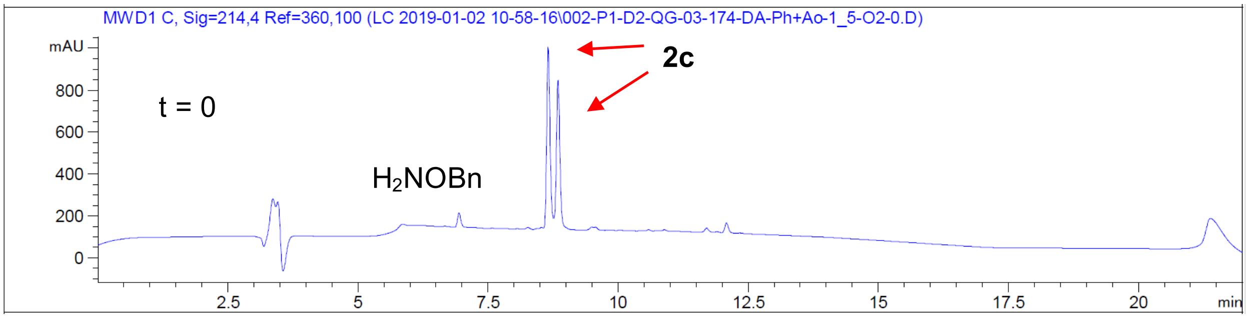

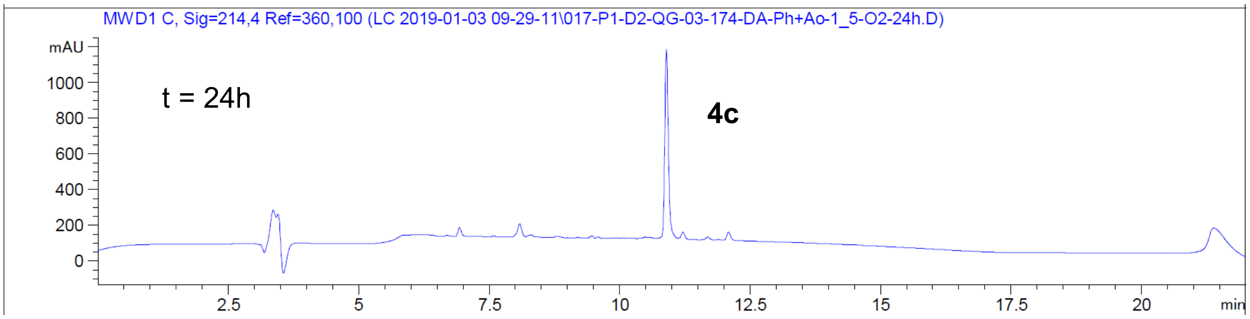

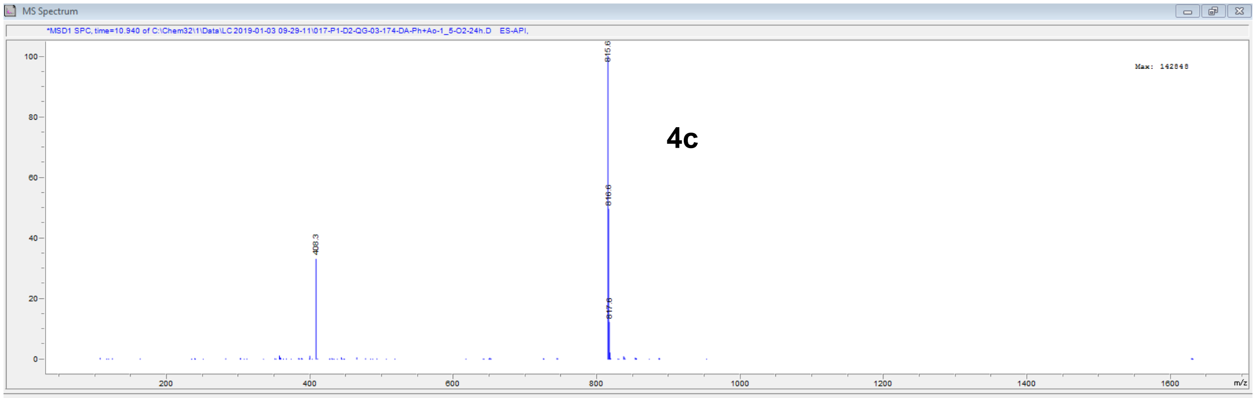

Ligation study of **2d** and *O*-benzylhydroxylamine hydrochloride (1:5 ratio) at **pH 7** under O_2 atmosphere:

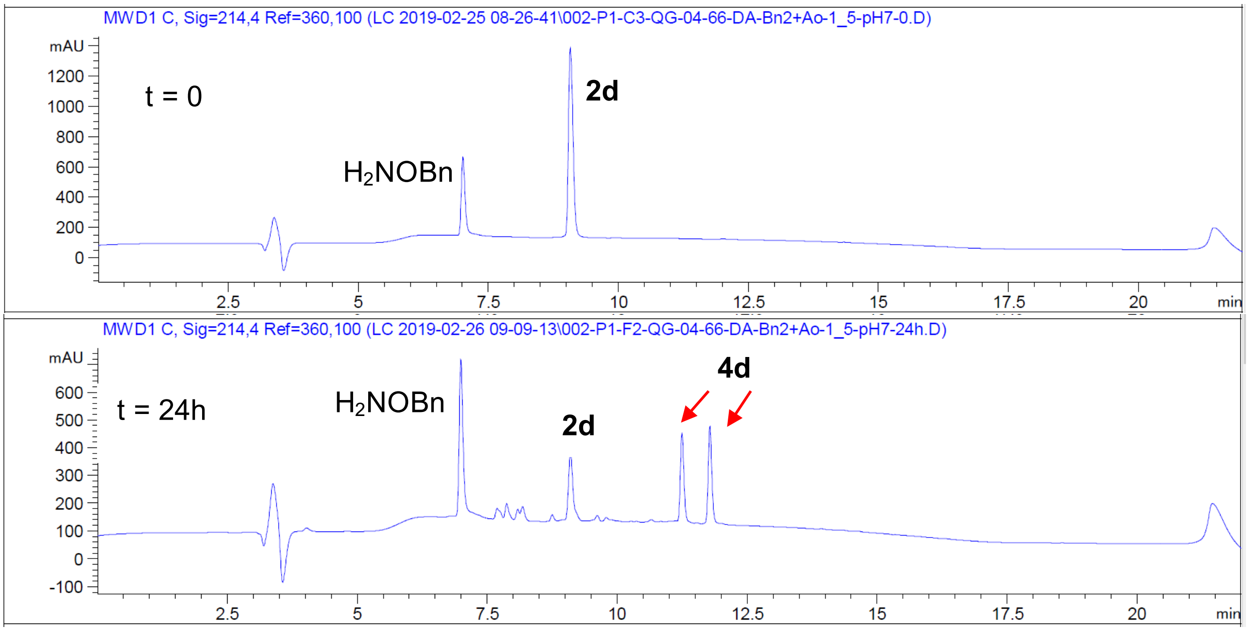

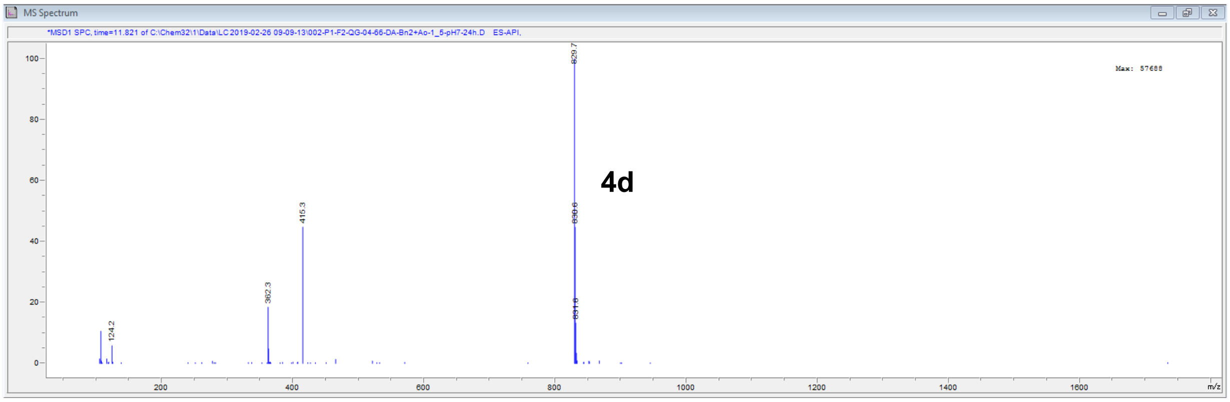

Ligation study of **2e** and *O*-benzylhydroxylamine hydrochloride (1:5 ratio) at **pH 7** under O_2 atmosphere:

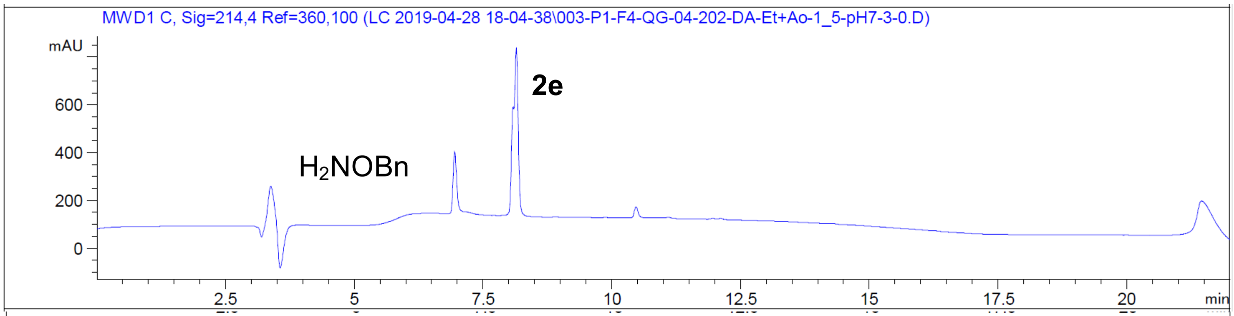

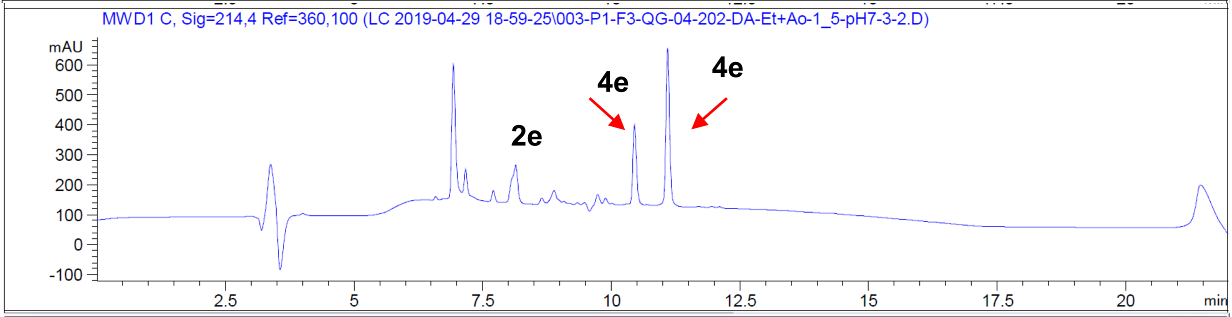

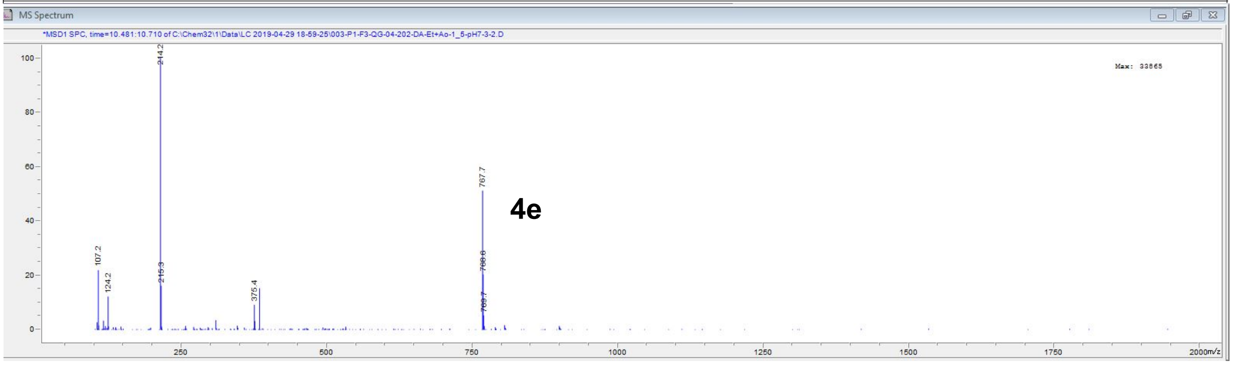

Ligation study of **2f** and *O*-benzylhydroxylamine hydrochloride (1:5 ratio) at **pH 7** under O_2 atmosphere:

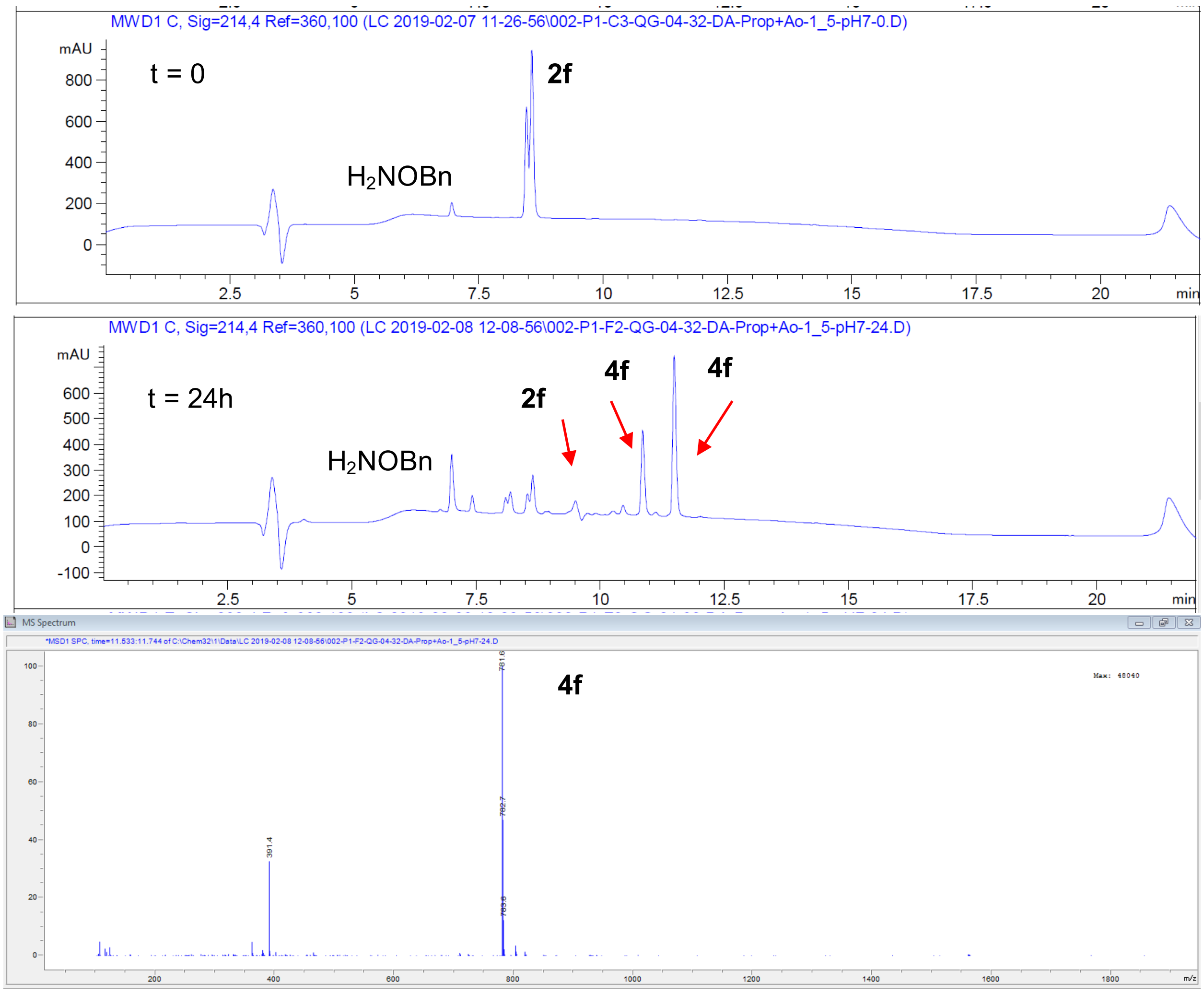

Ligation study of **2g** and *O*-benzylhydroxylamine hydrochloride (1:5 ratio) at **pH 7** under O_2 atmosphere:

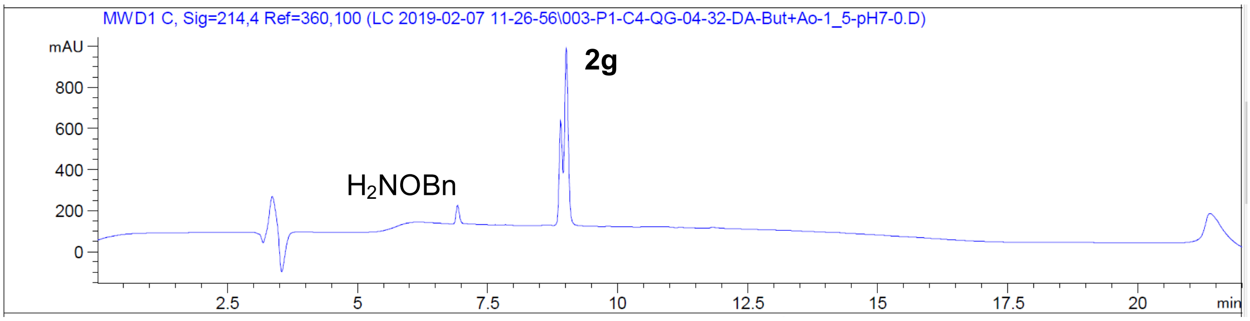

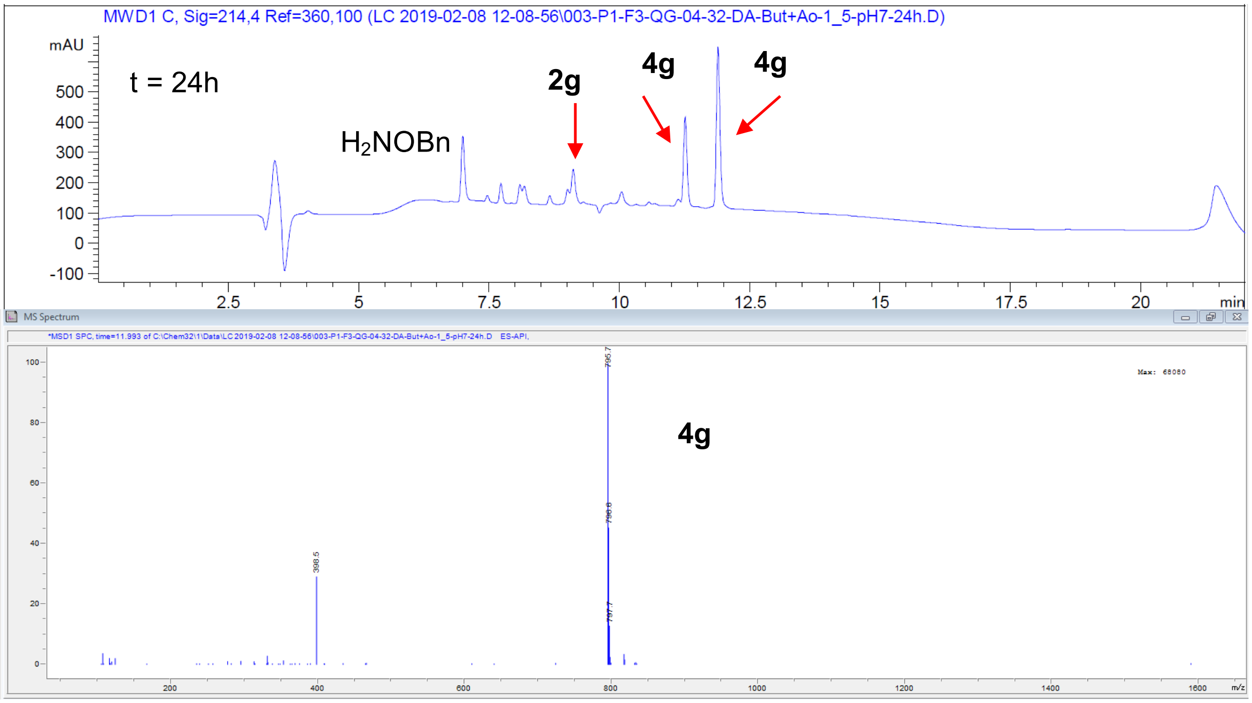

Ligation study of **2h** and *O*-benzylhydroxylamine hydrochloride (1:5 ratio) at **pH 7** with 25% EtOH under O_2 atmosphere:

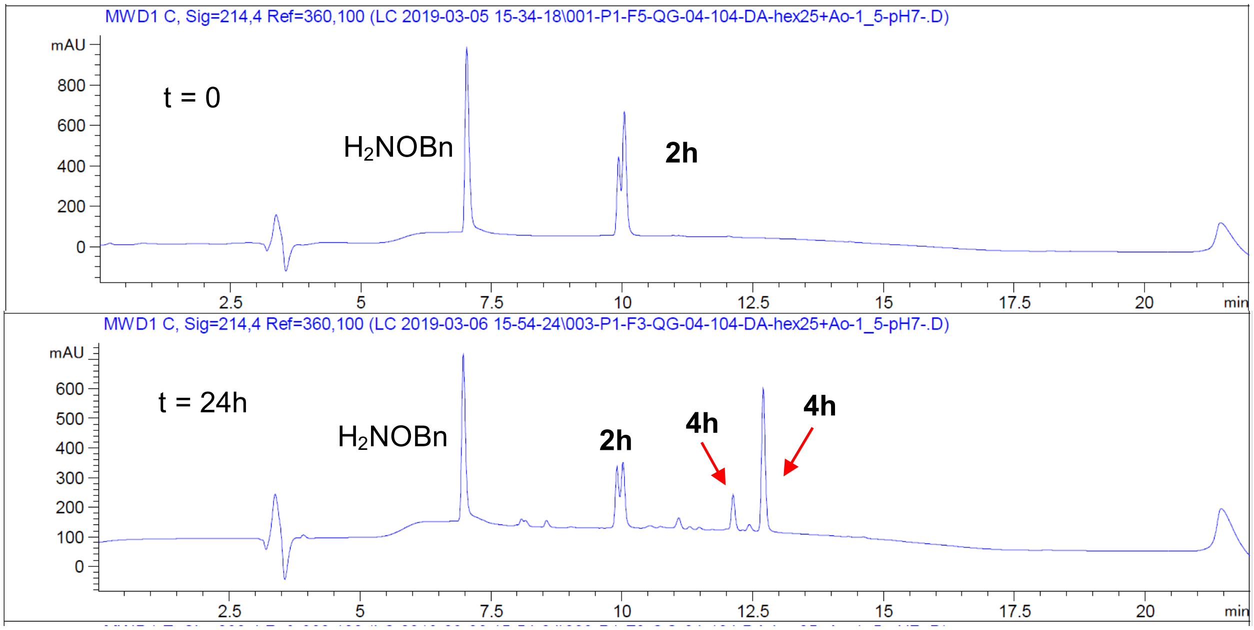

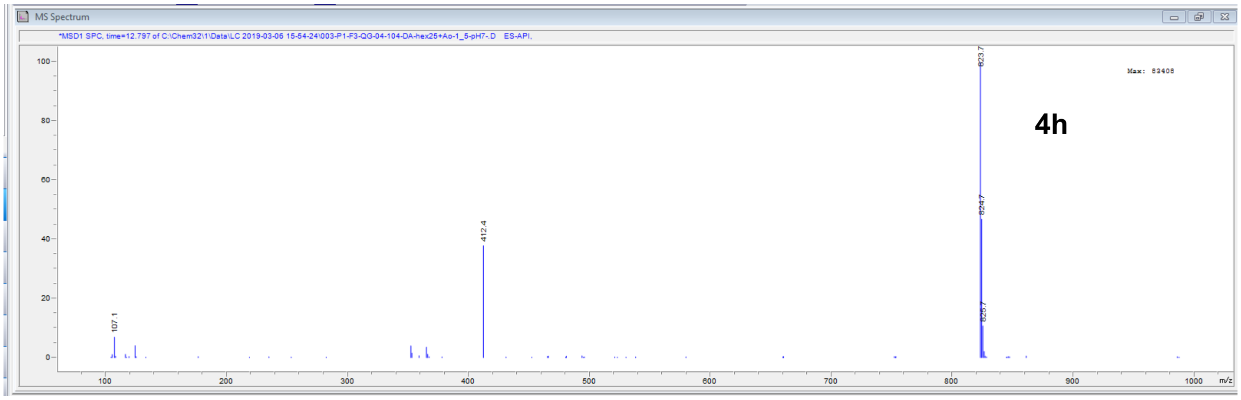

Ligation study of **2i** and *O*-benzylhydroxylamine hydrochloride (1:5 ratio) at **pH 7** with 50% EtOH under O_2 atmosphere:

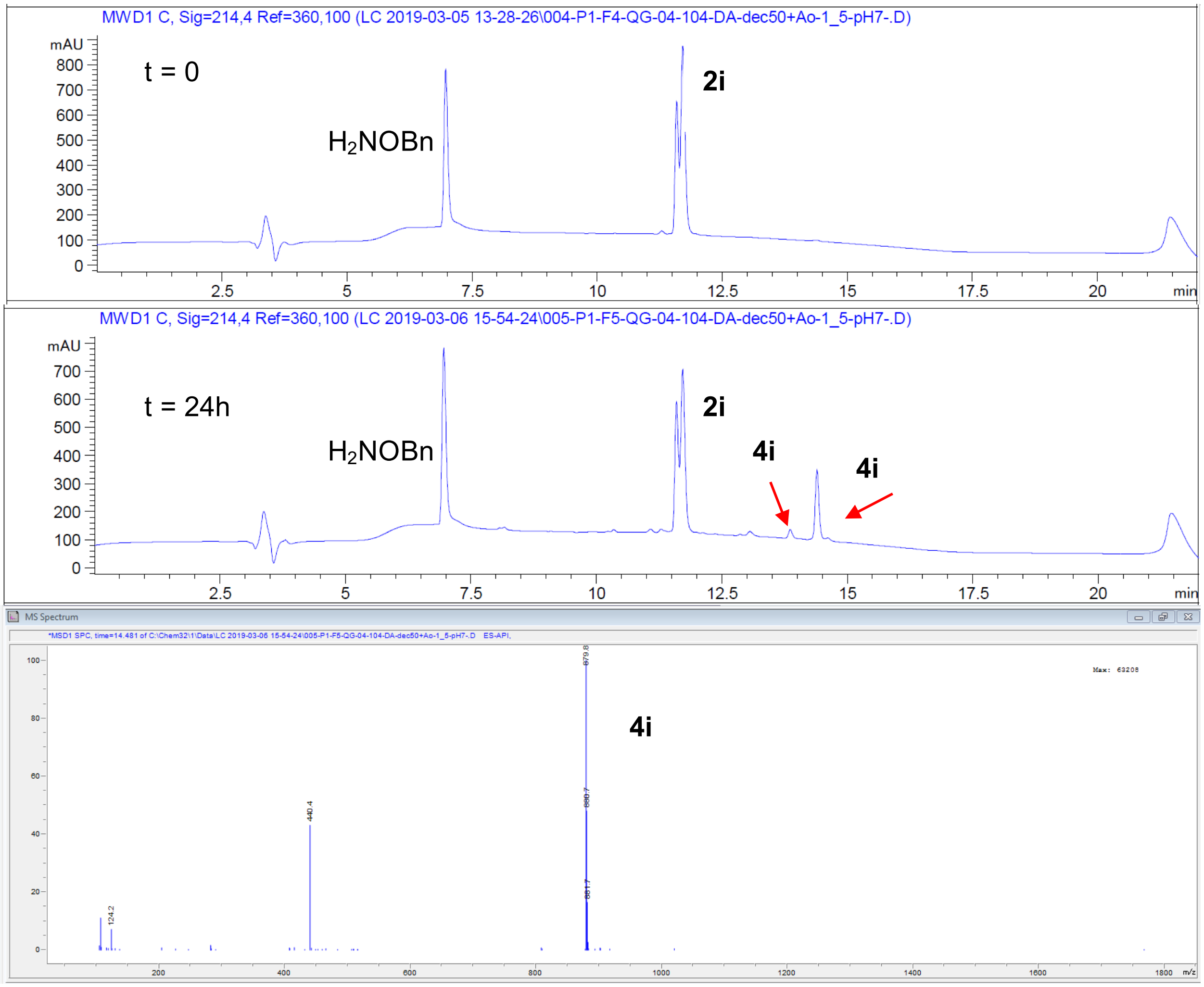

Ligation study of **2j** and *O*-benzylhydroxylamine hydrochloride (1:5 ratio) at **pH 7** under O_2 atmosphere:

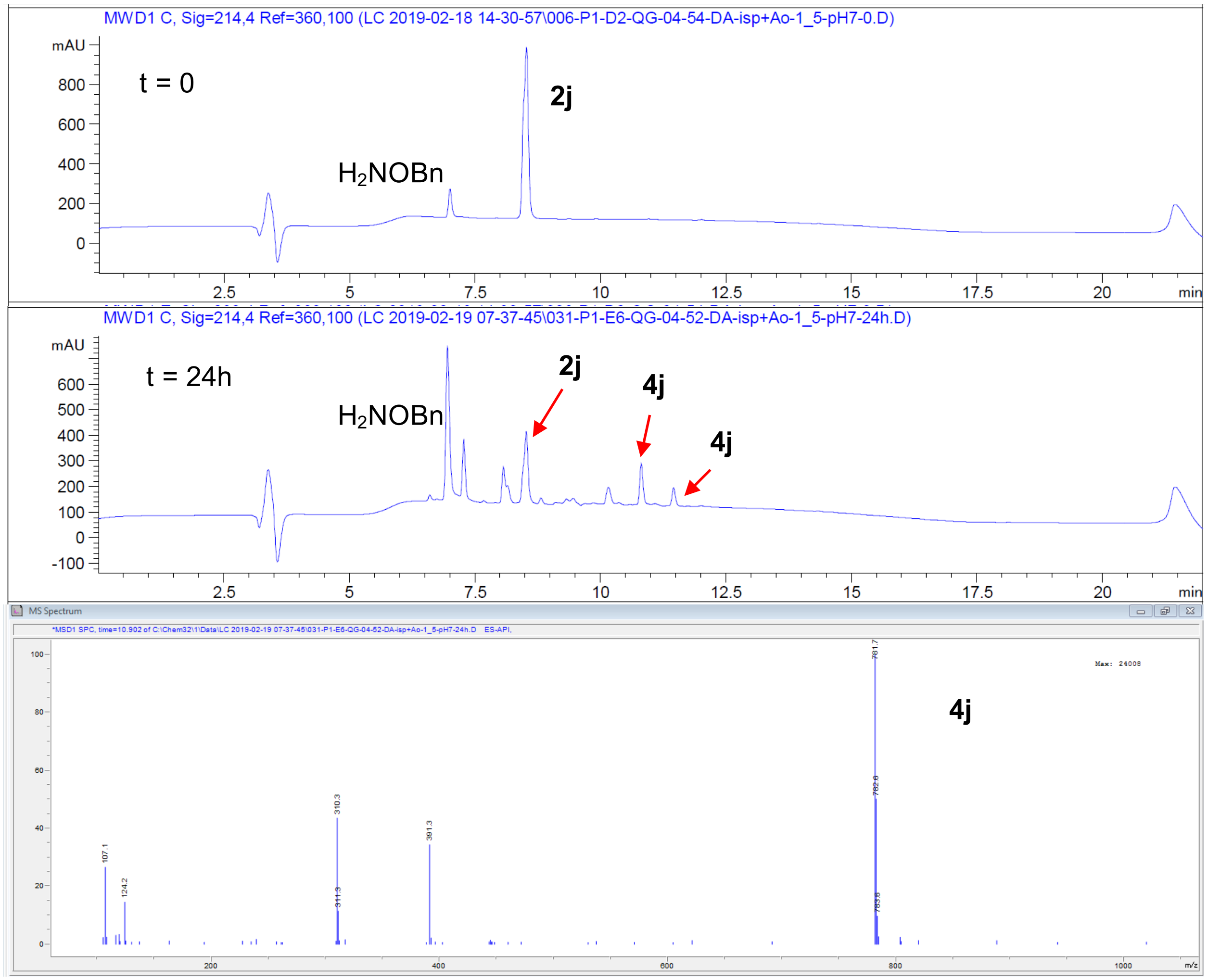

Ligation study of **3b** and *O*-benzylhydroxylamine hydrochloride (1:1 ratio) at **pH 4.5** under air, showing no reactivity:

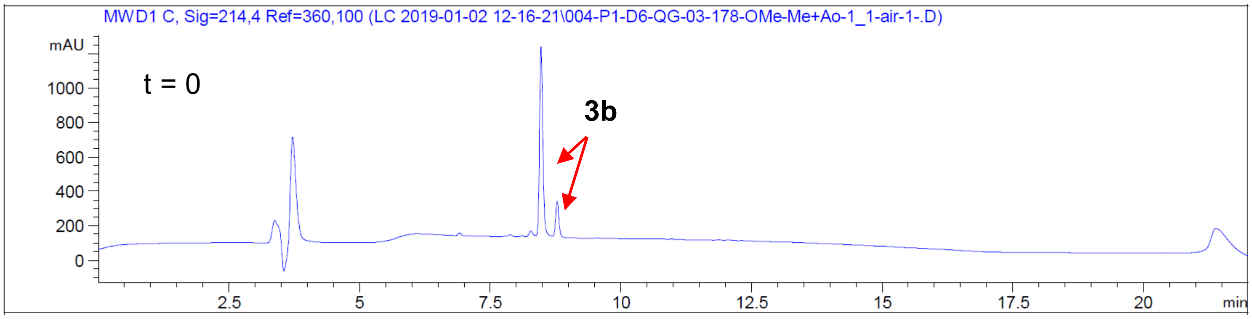

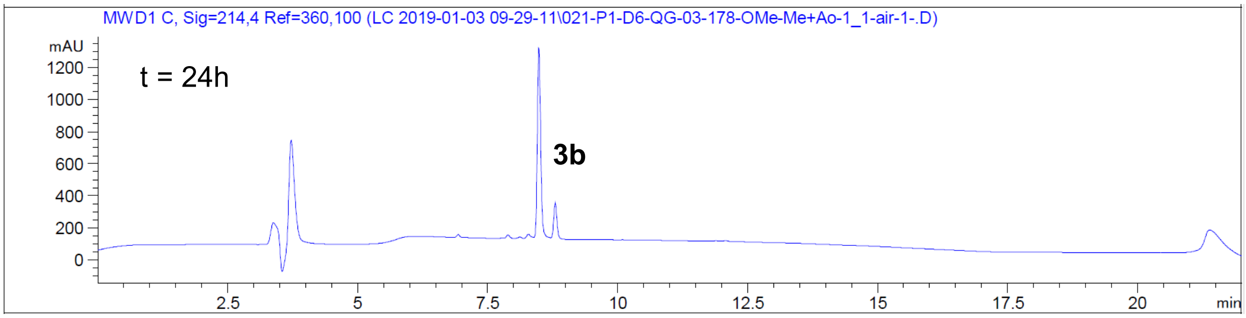

Ligation study of **3b** and *O*-benzylhydroxylamine hydrochloride (1:5 ratio) at **pH 7** under O_2 atmosphere, showing no reactivity:

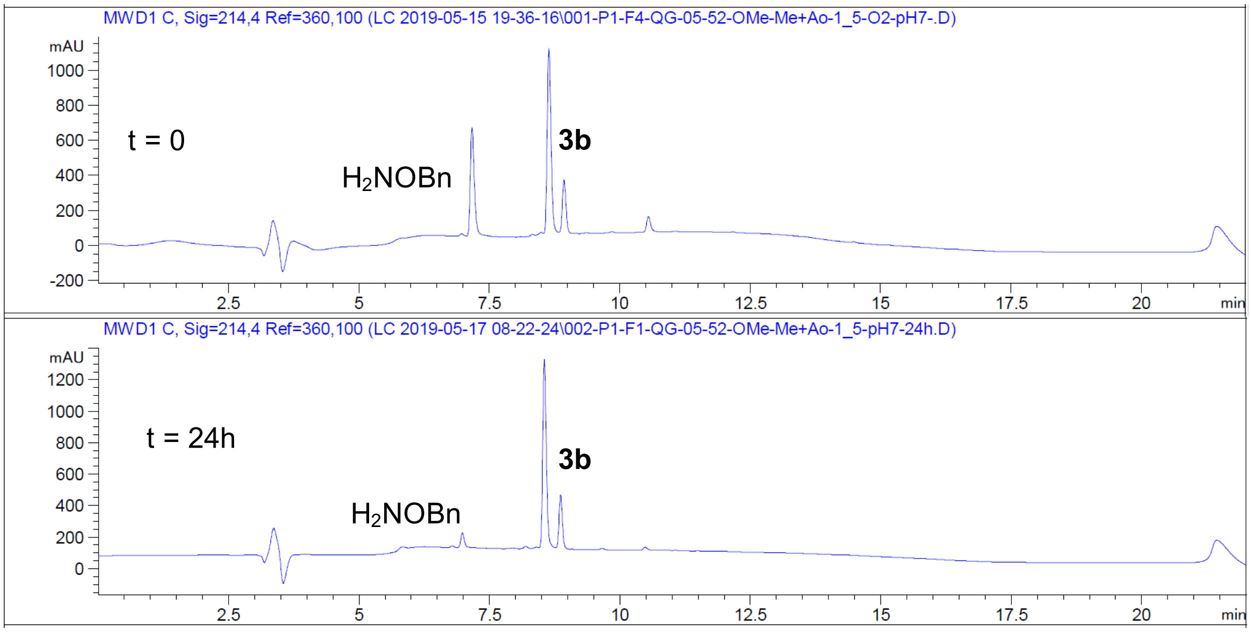

*<u>Note:</u> Ligation studies with peptides **3c-d** were conducted and also showed no ketoxime formation. The traces, showing starting material only, have been excluded.*

Ligation study of **3b** and *O*-benzylhydroxylamine hydrochloride (1: 5 ratio) at **pH 7** with 1 mM potassium ferricyanide, showing no reactivity:

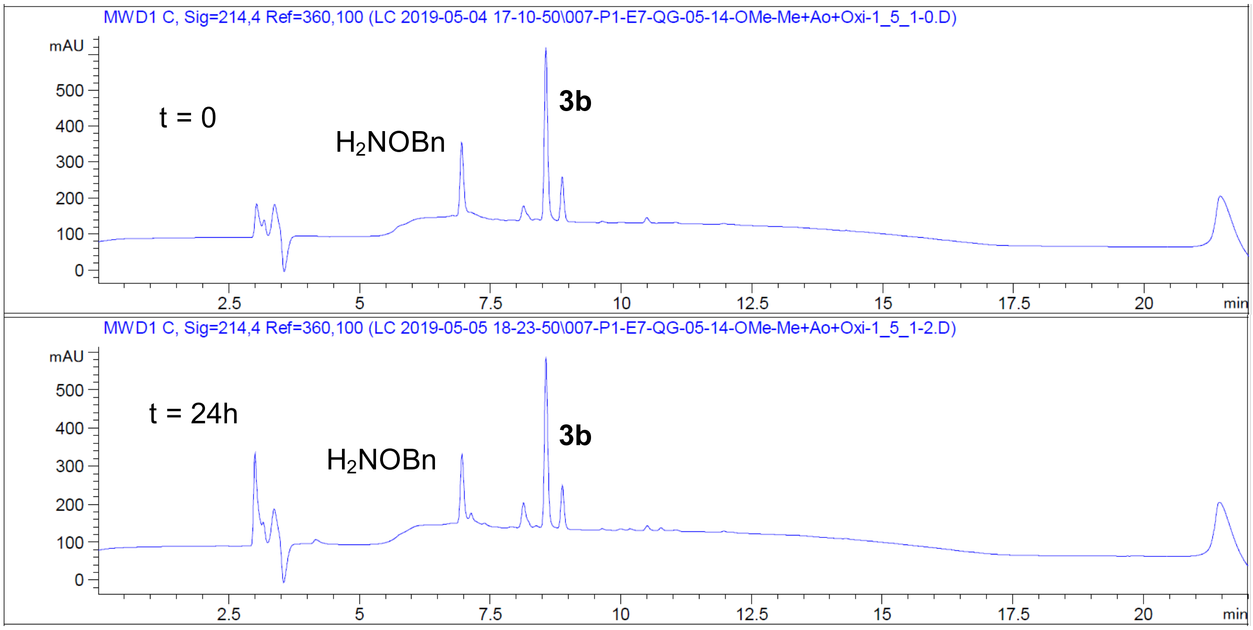

Control Ligation study of **2b**, EDTA and *O*-benzylhydroxylamine hydrochloride (1:10: 5 ratio) at **pH 7** under O_2 balloon:

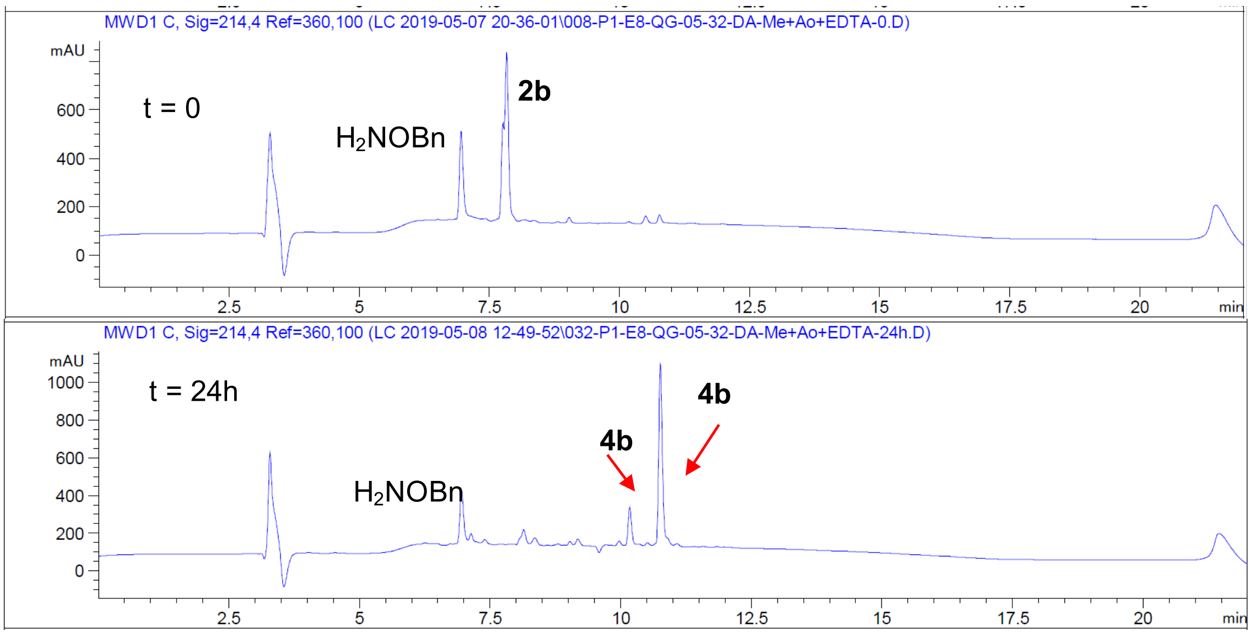

Oxidation followed by ligation study of **2b** and *O*-benzylhydroxylamine hydrochloride (1:5 ratio) at **pH 7** under O_2 atmosphere:

Oxidation:

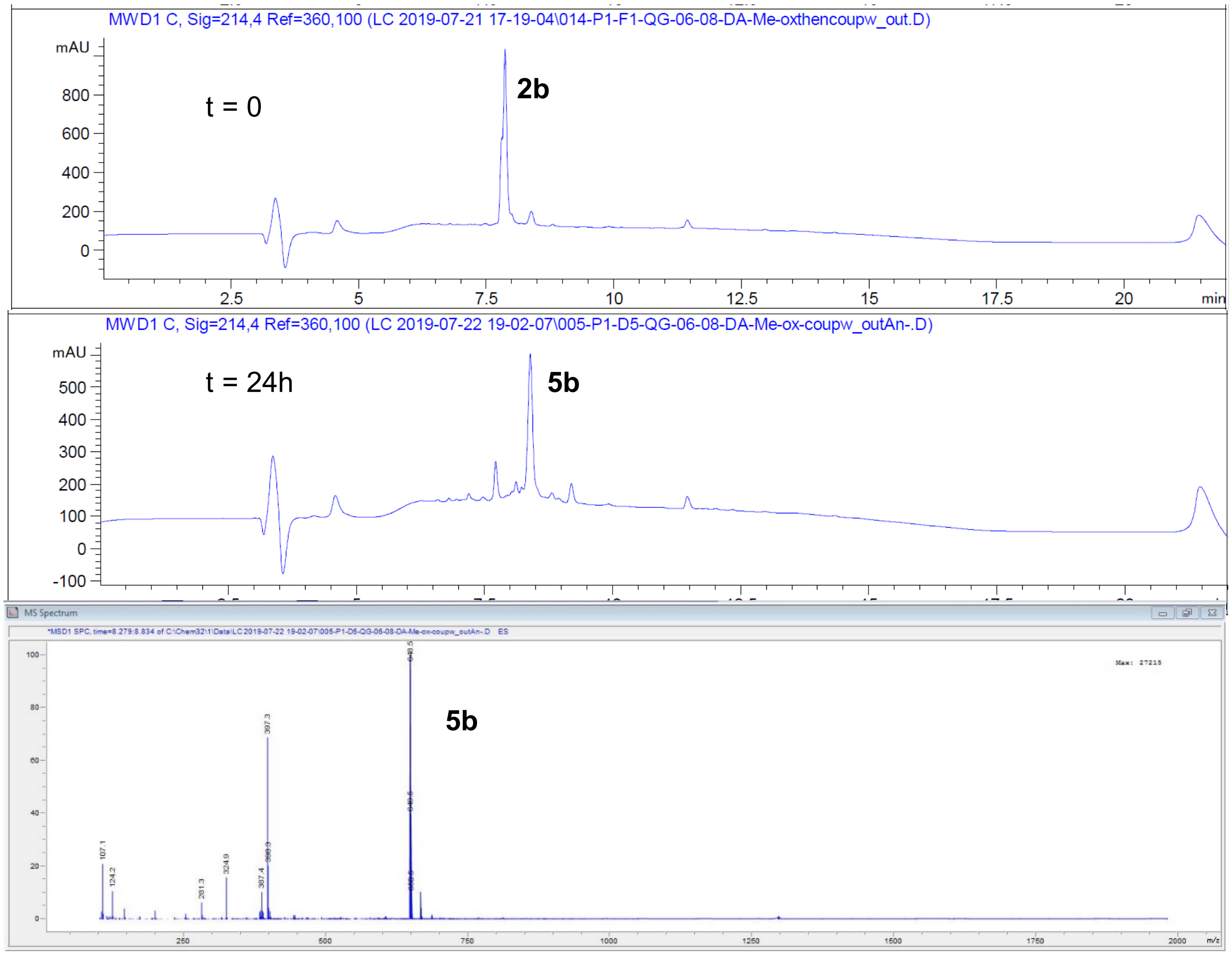

Ligation without aniline:

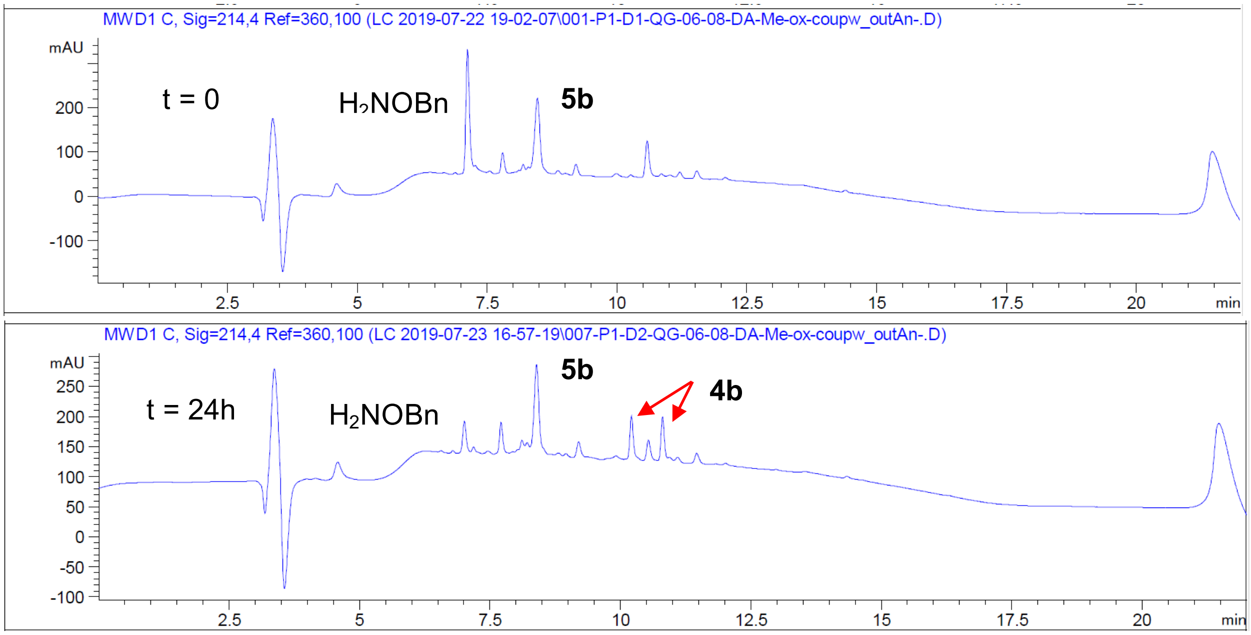

Ligation with aniline:

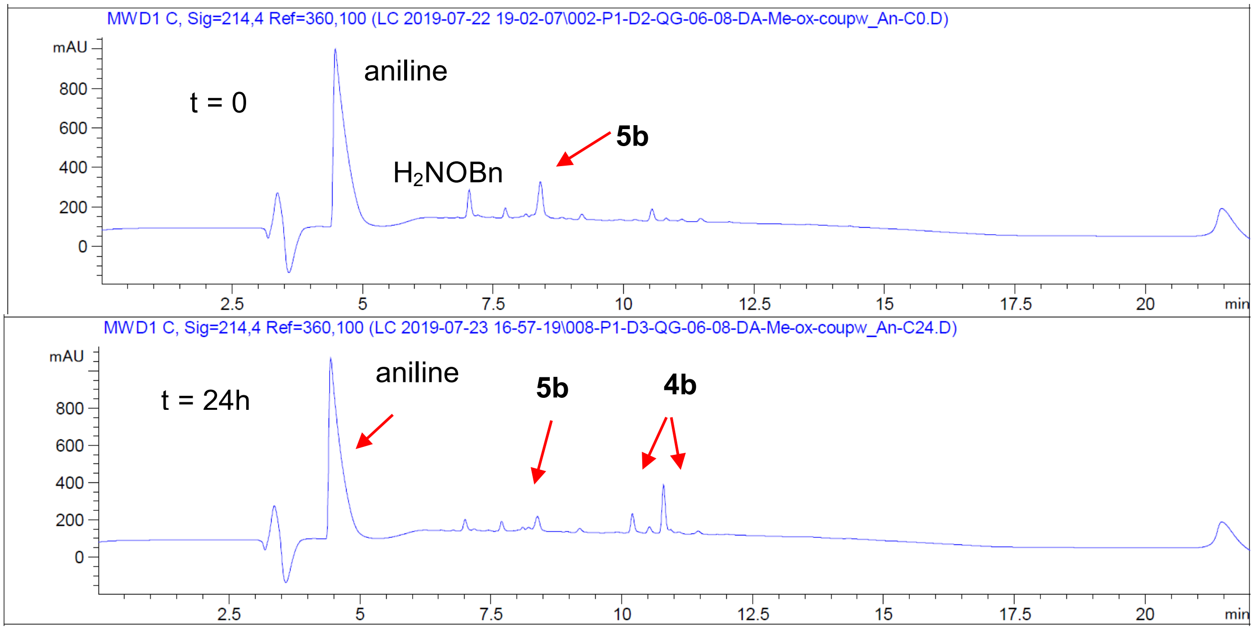

Oxidation followed by ligation study of **2c** and *O*-benzylhydroxylamine hydrochloride (1:5 ratio) at **pH 7** under O_2 atmosphere:

Oxidation:

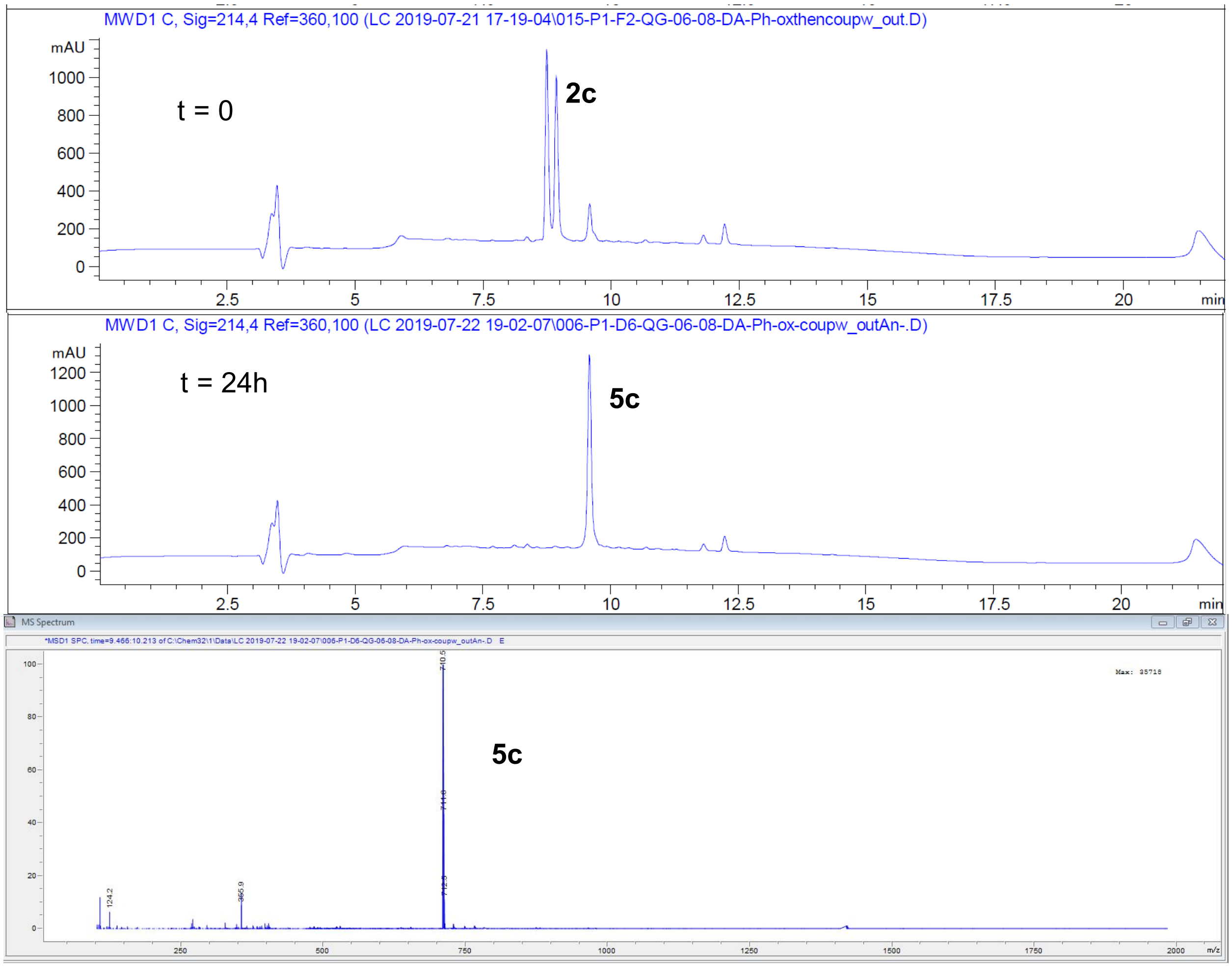

Ligation without aniline:

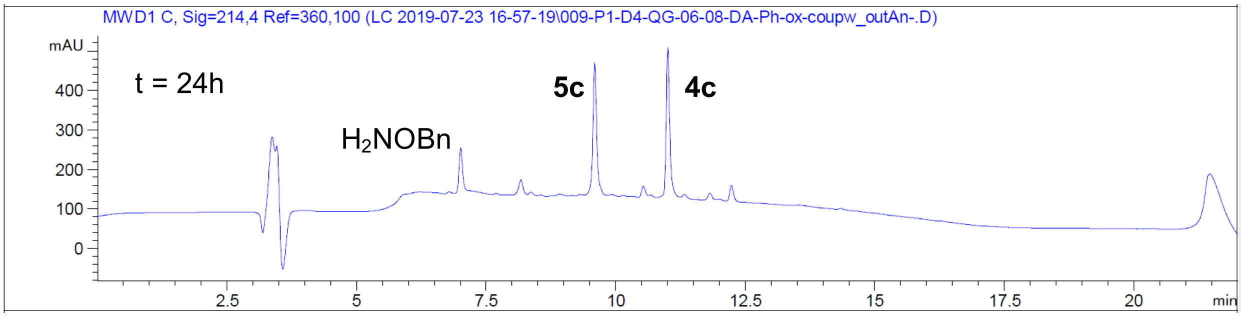

Ligation with aniline:

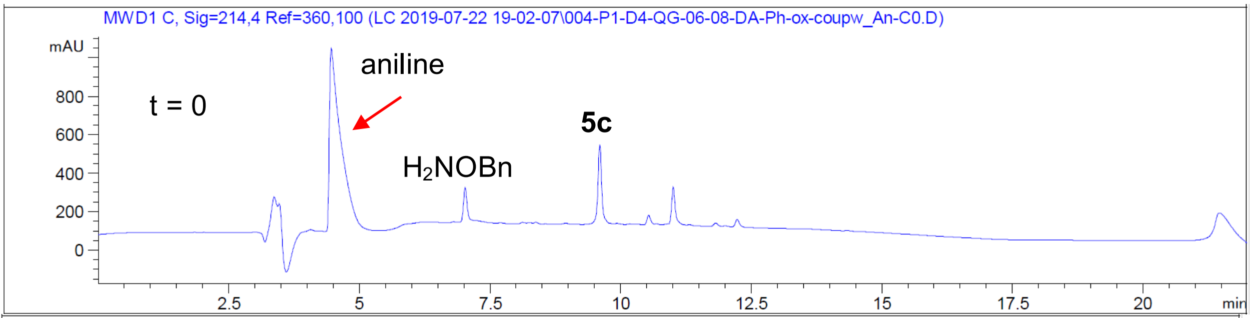

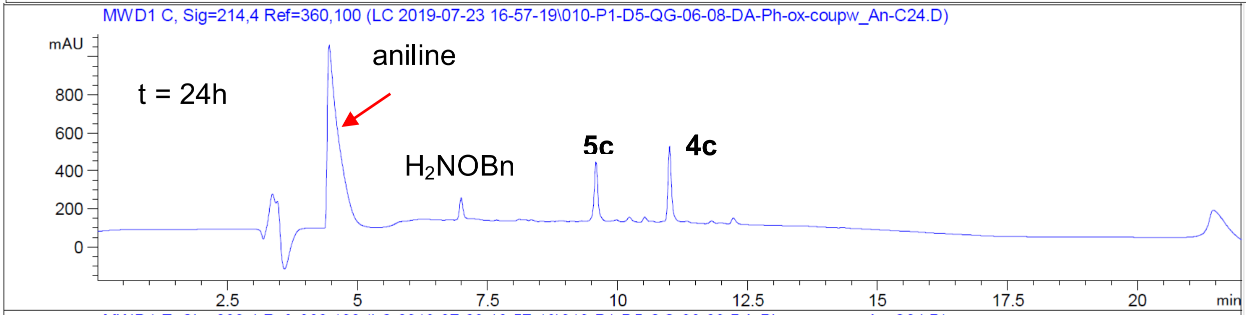

Oxidation followed by ligation study of **2d** and *O*-benzylhydroxylamine hydrochloride (1:5 ratio) at **pH 7** under O_2 atmosphere:

Oxidation:

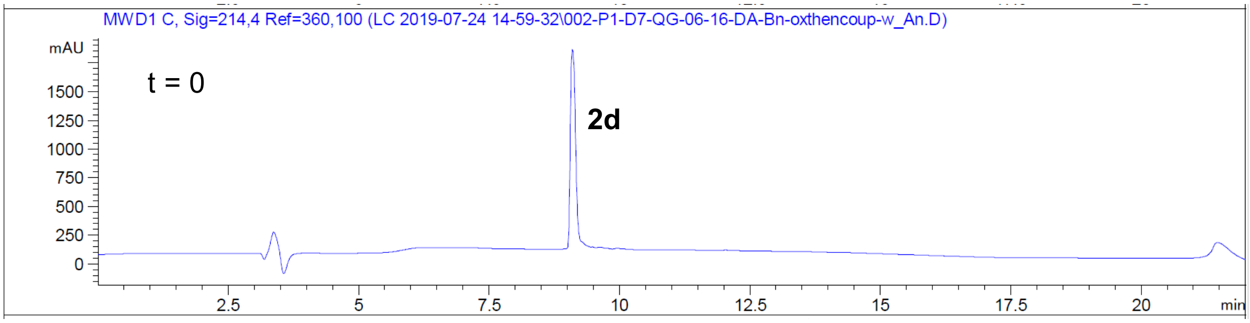

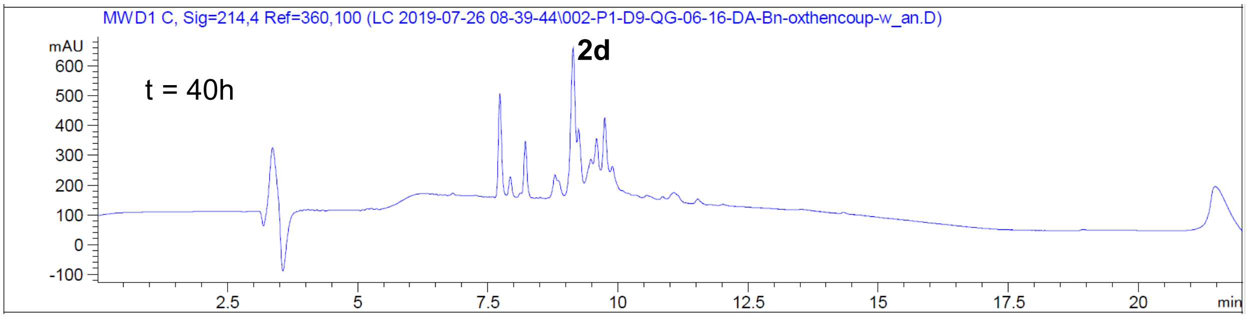

Note: The ligation is excluded due to our inability to identify the ketone after oxidation.

Ligation study of ***N*-(*p*-NMe$_2$-Ph)A-WYRAG** and *O*-benzylhydroxylamine hydrochloride (1:5 ratio) at **pH 7** under O_2 atmosphere:

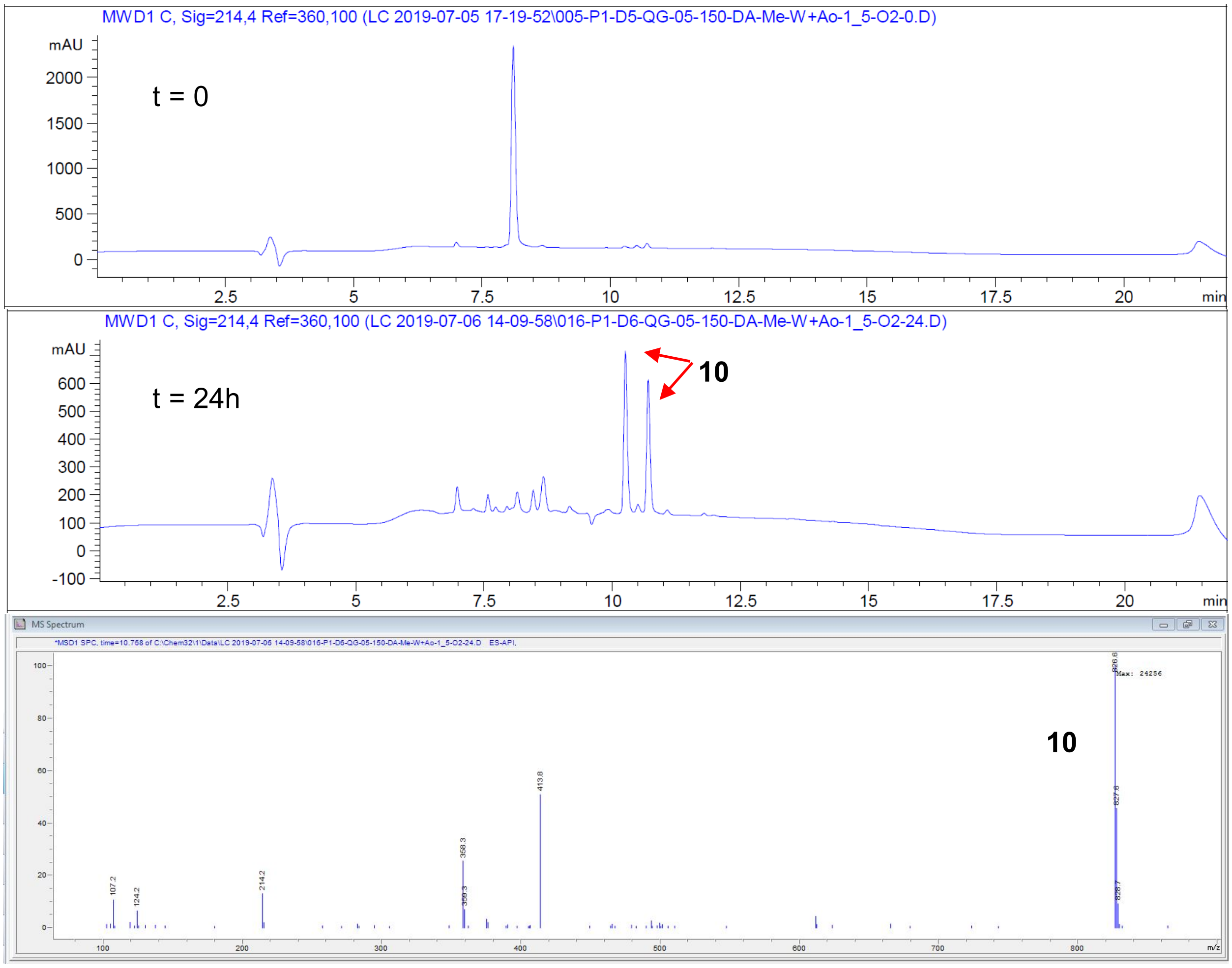

Ligation study of ***N*-(*p*-NMe$_2$-Ph)A-CYRAG** and *O*-benzylhydroxylamine hydrochloride (1:5 ratio) at **pH 7** under O_2 atmosphere:

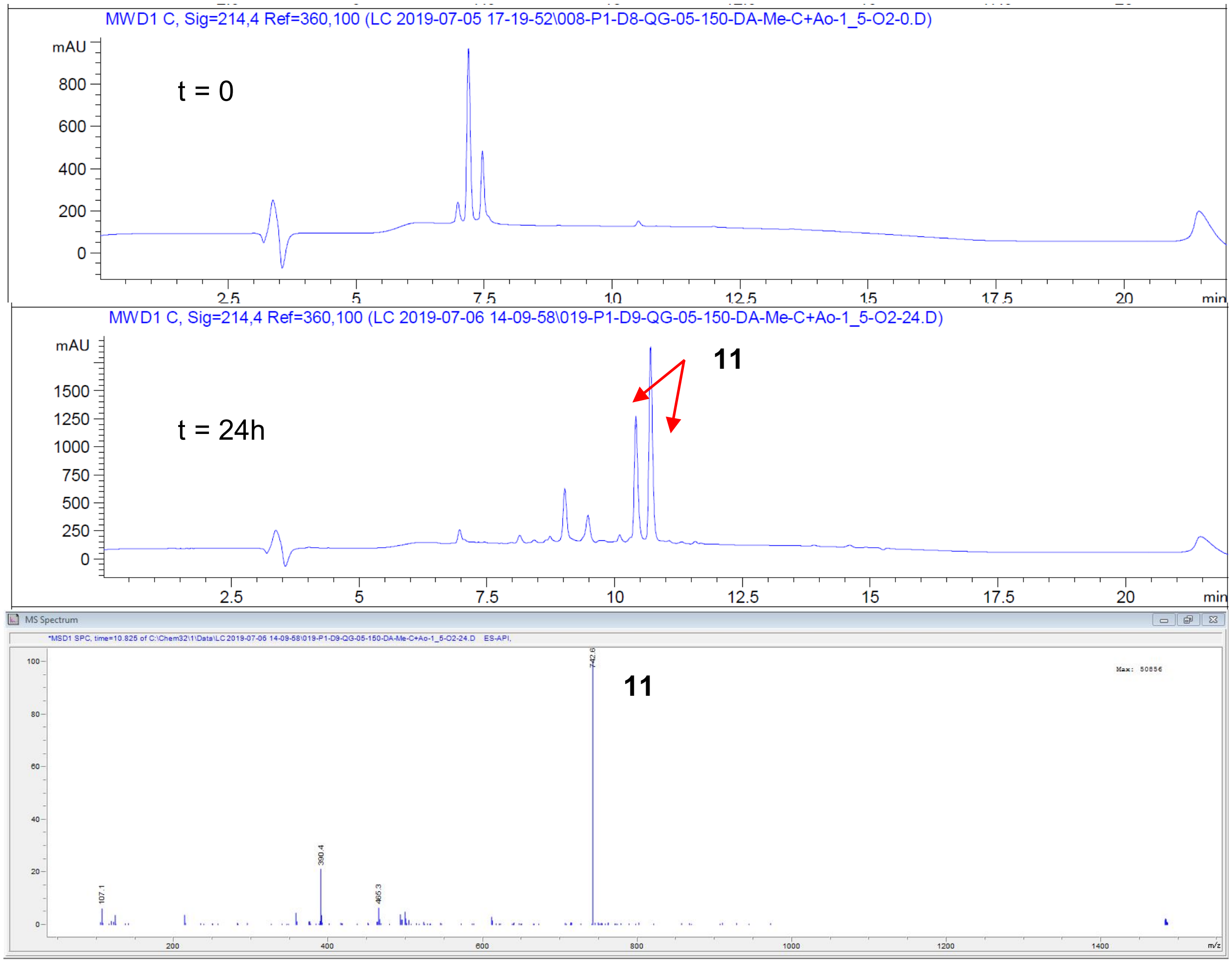

Ligation study of ***N*-(*p*-NMe$_2$-Ph)A-SYRAG** and *O*-benzylhydroxylamine hydrochloride (1:5 ratio) at **pH 7** under O_2 atmosphere:

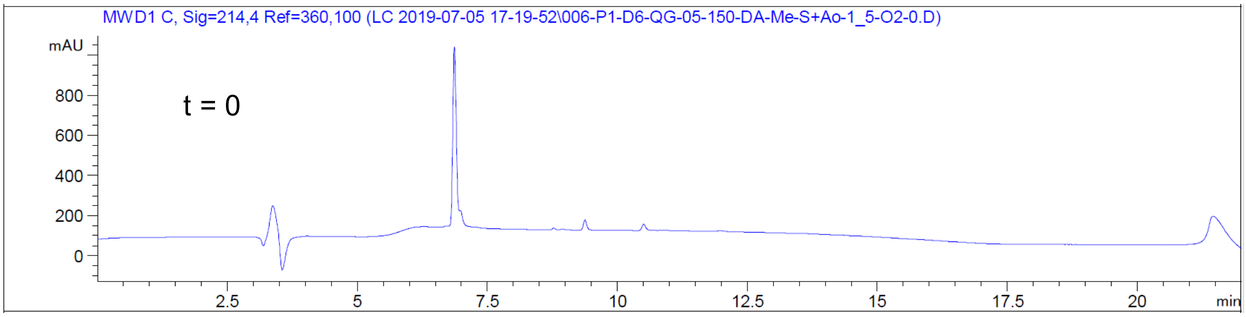

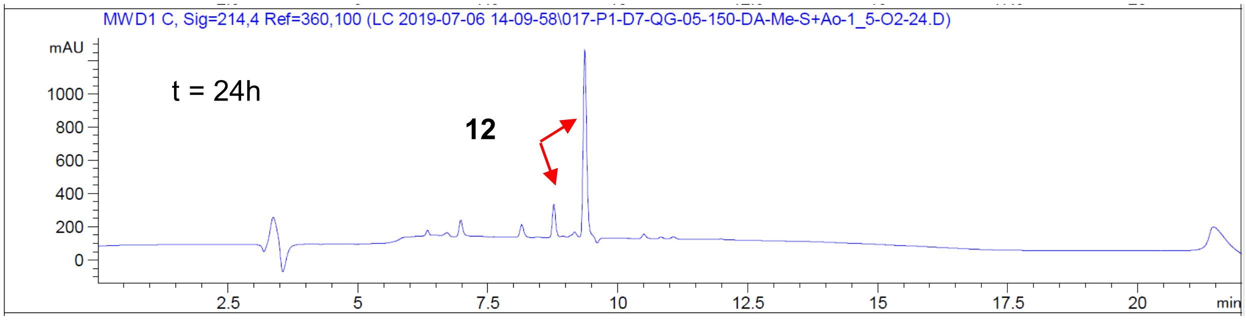

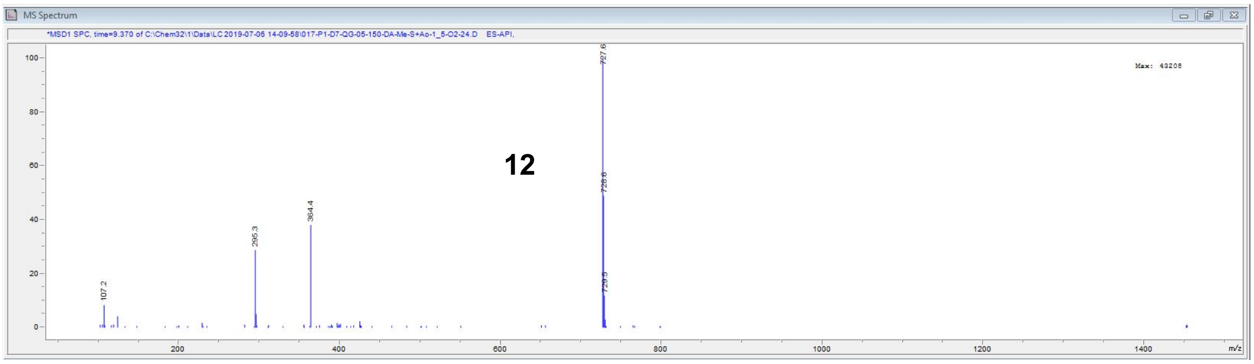

Ligation study of ***N*-(*p*-NMe$_2$-Ph)A-KYRAG** and *O*-benzylhydroxylamine hydrochloride (1:5 ratio) at **pH 7** under O_2 atmosphere:

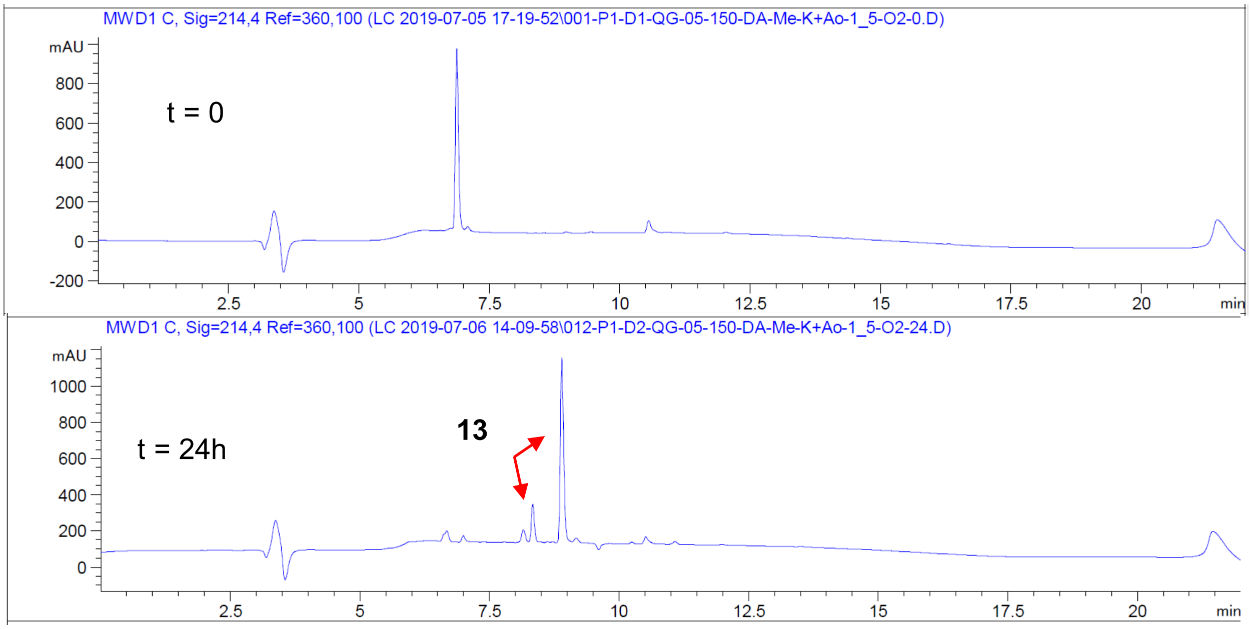

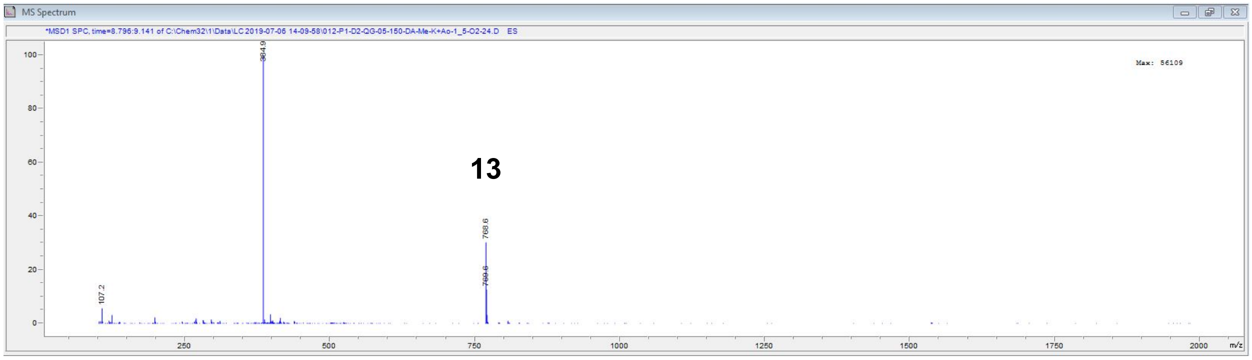

Ligation study of ***N*-(*p*-NMe$_2$-Ph)A-EYRAG** and *O*-benzylhydroxylamine hydrochloride (1:5 ratio) at **pH 7** under O_2 atmosphere:

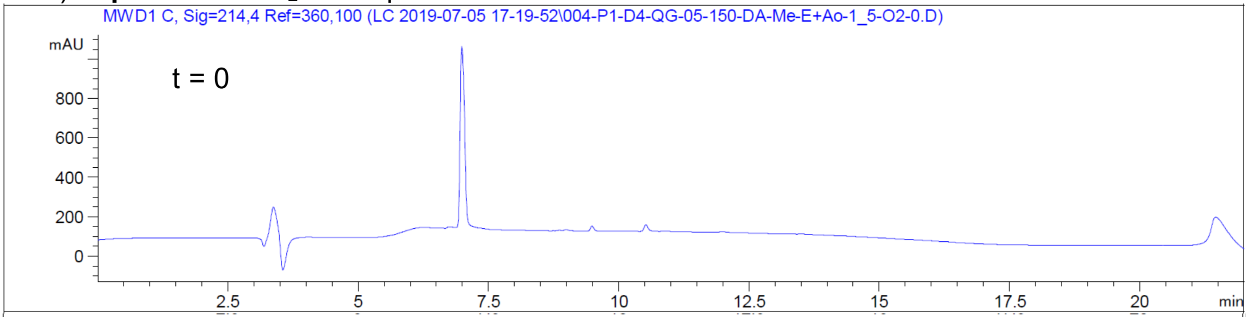

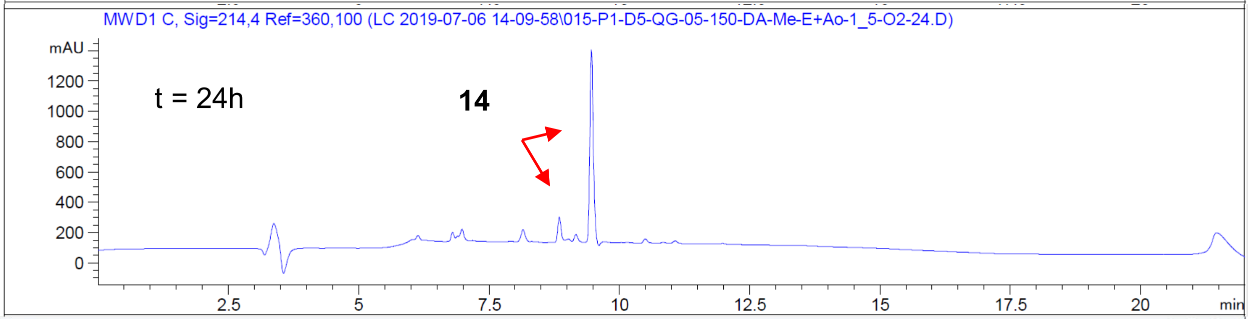

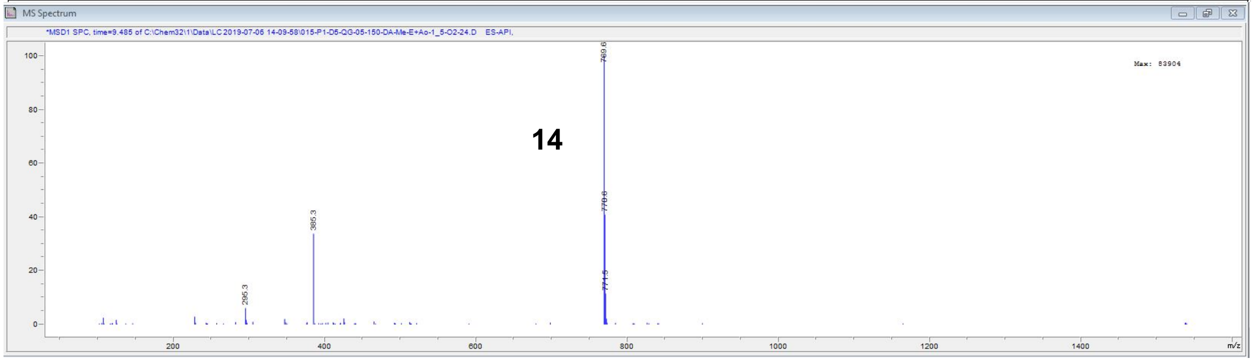

Ligation study of ***N*-(*p*-NMe$_2$-Ph)A-QYRAG** and *O*-benzylhydroxylamine hydrochloride (1:5 ratio) at **pH 7** under O_2 atmosphere:

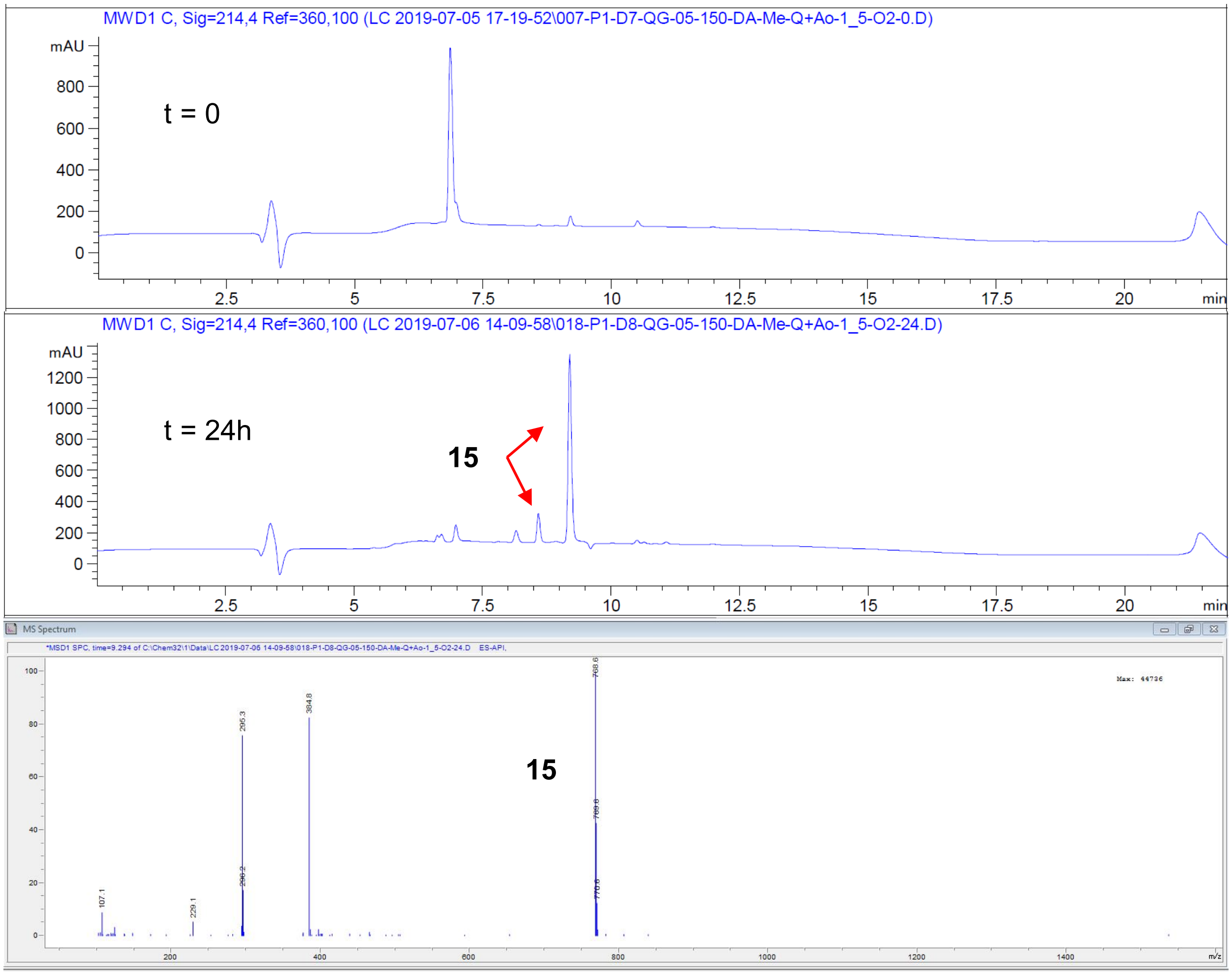

Ligation study of **2b** and aminooxyacetyl-GRGDSGG **16** (1:10 ratio) at **pH 7** under O_2 balloon:

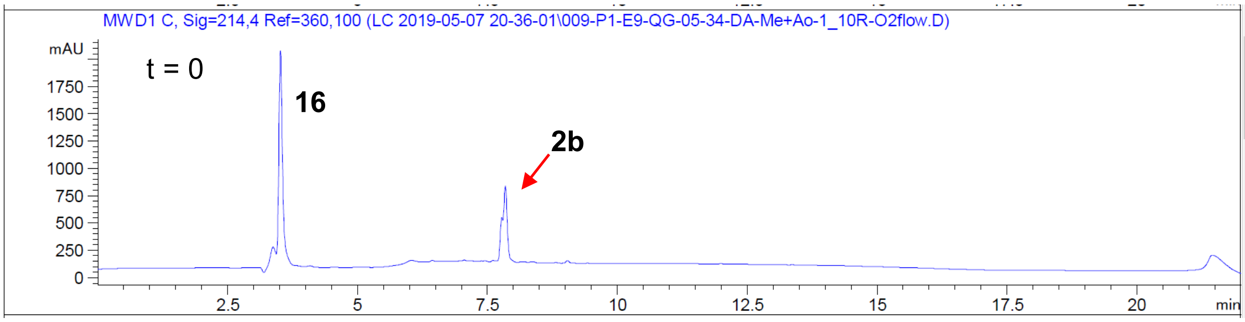

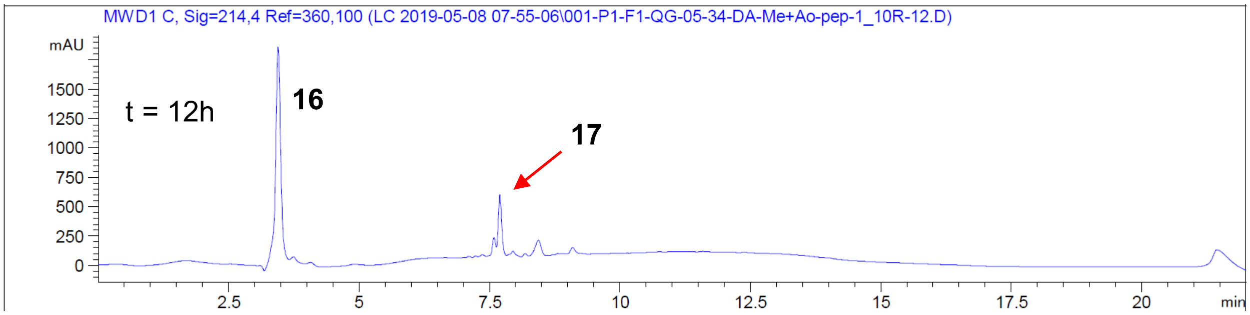

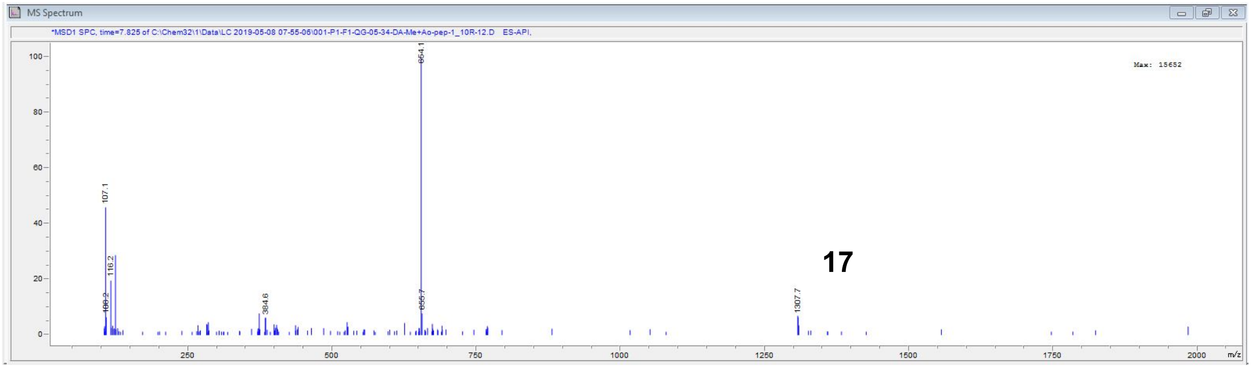

Ligation study of **2c** and aminooxyacetyl-GRGDSGG **16** (1:10 ratio) at **pH 7** under O_2 balloon:

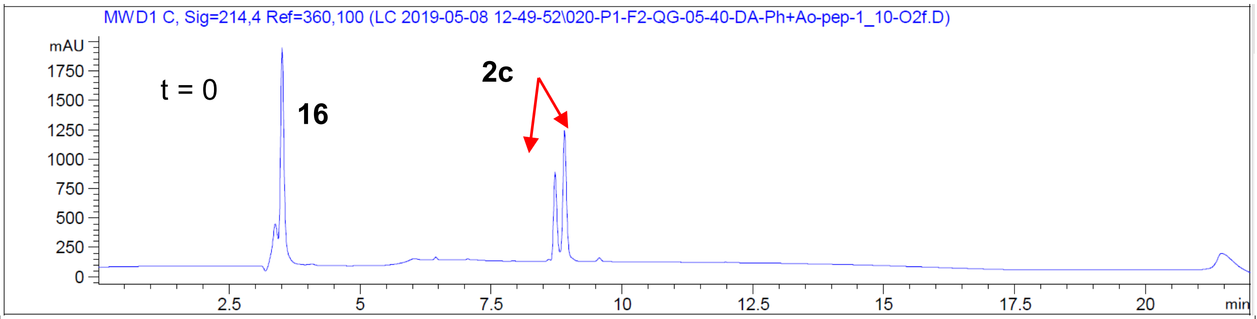

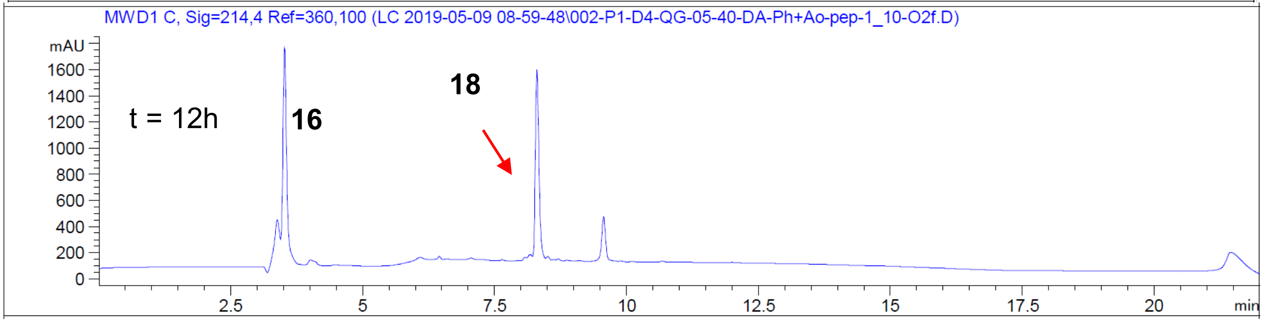

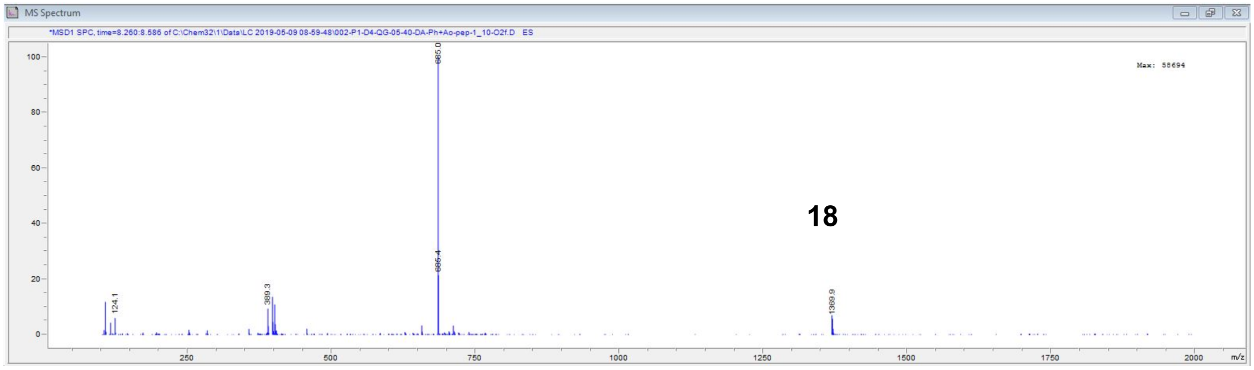

Ligation study of **2d** and aminooxyacetyl-GRGDSGG **16** (1:10 ratio) at **pH 7** under O_2 balloon:

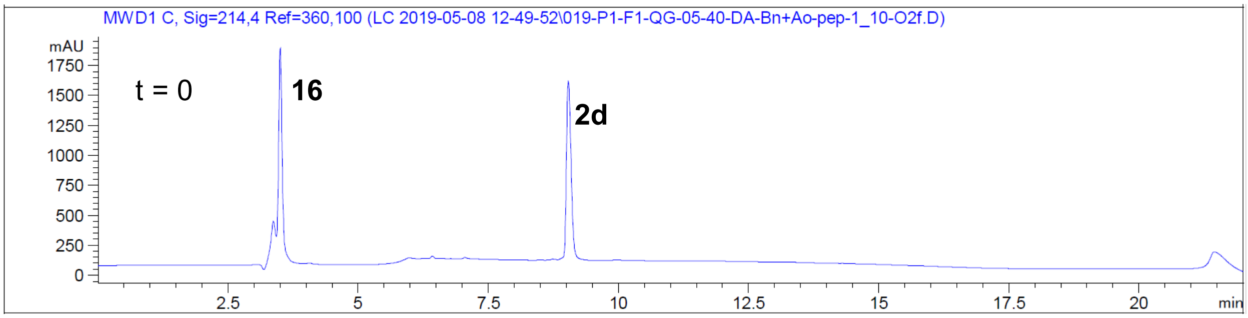

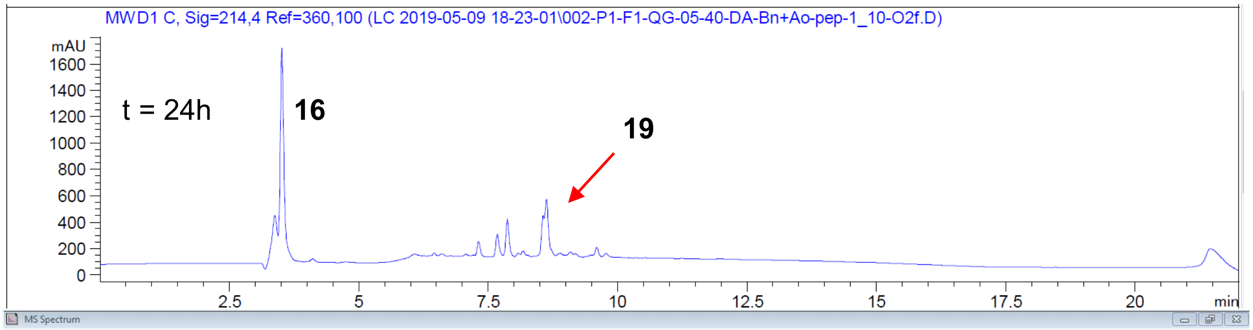

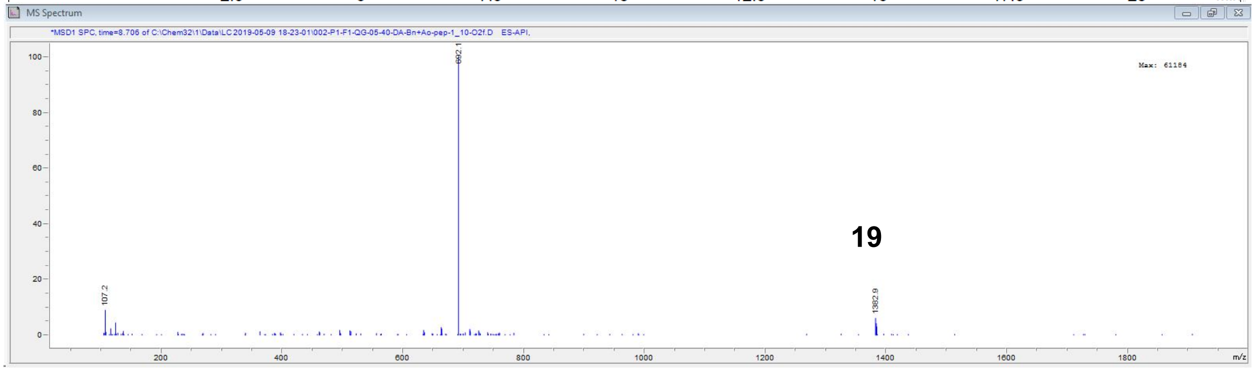

4. NMR Spectra

^{1}H NMR spectra of representative ketoximes **4b-d** and **17-19** can be found below:

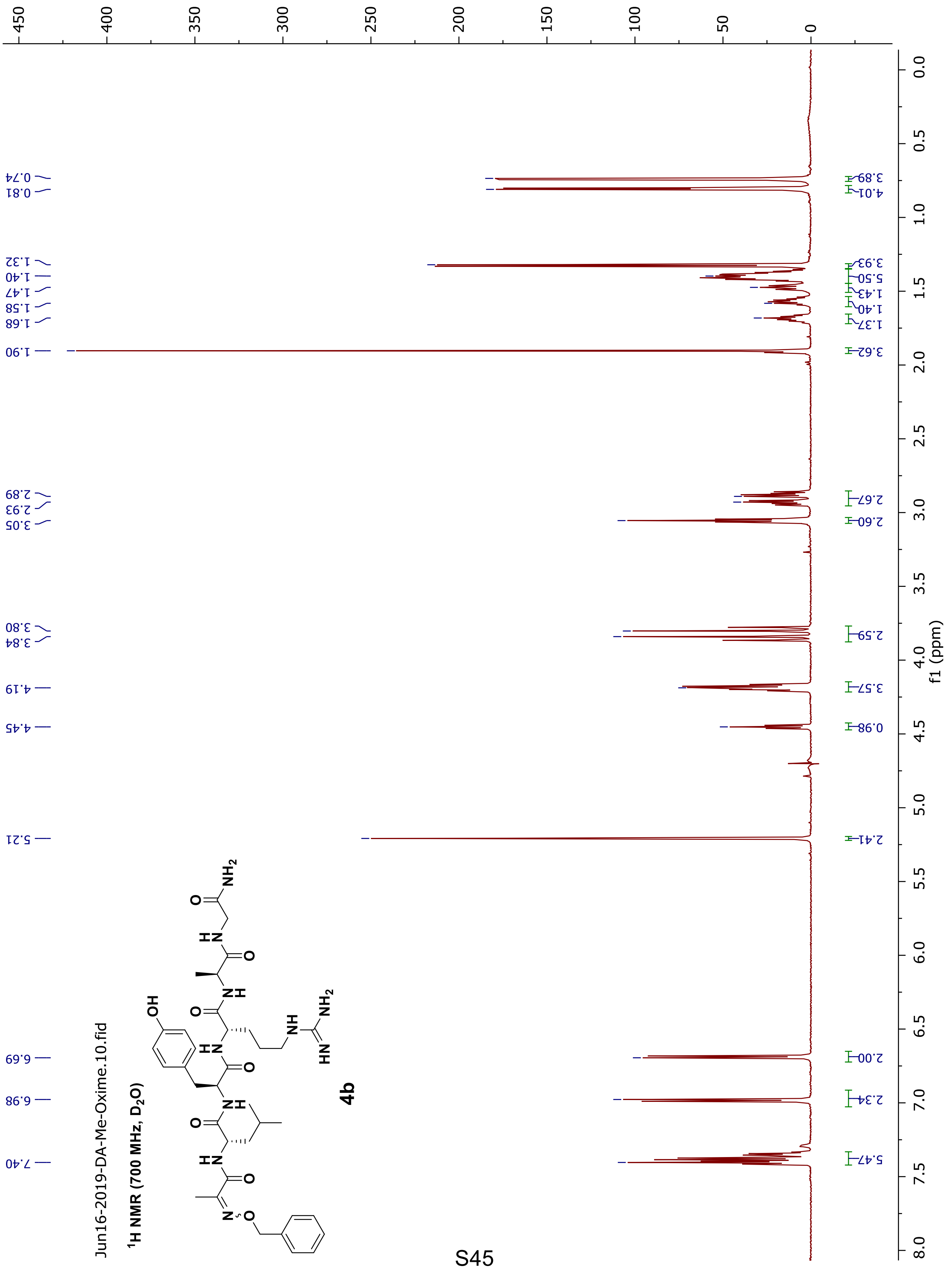

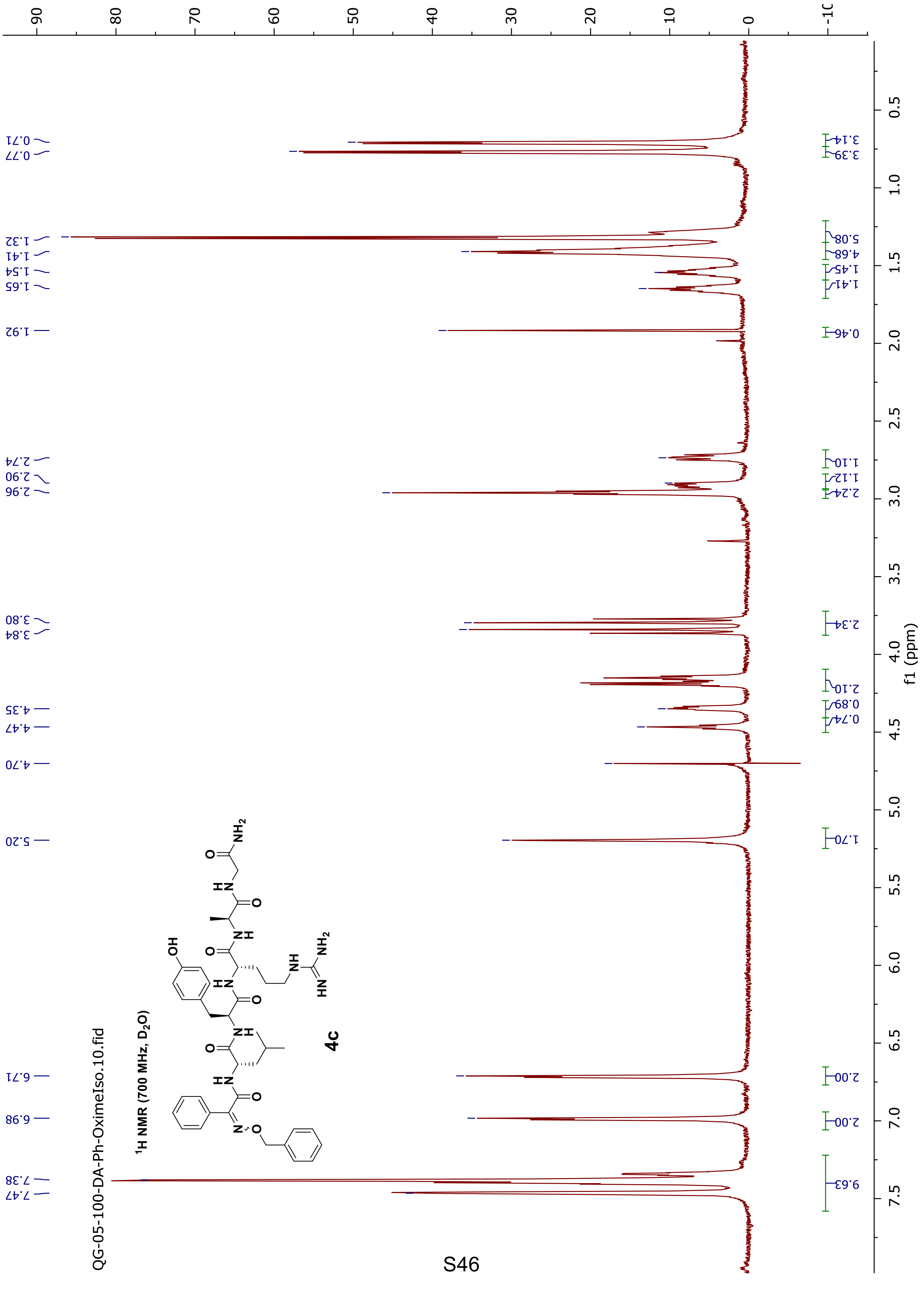
QG-05-100-DA-Ph-OximeIso.10.fid
^{1}H NMR (700 MHz, D$_2$O)
4c
0.71
0.77
1.32
1.41
1.54
1.65
1.92
2.74
2.90
2.96
3.80
3.84
4.35
4.47
4.70
5.20
6.71
6.98
7.38
7.47
3.14
3.39
5.08
4.68
1.45
1.41
0.46
1.10
1.12
2.24
2.34
2.10
0.89
0.74
1.70
2.00
2.00
9.63
f1 (ppm)

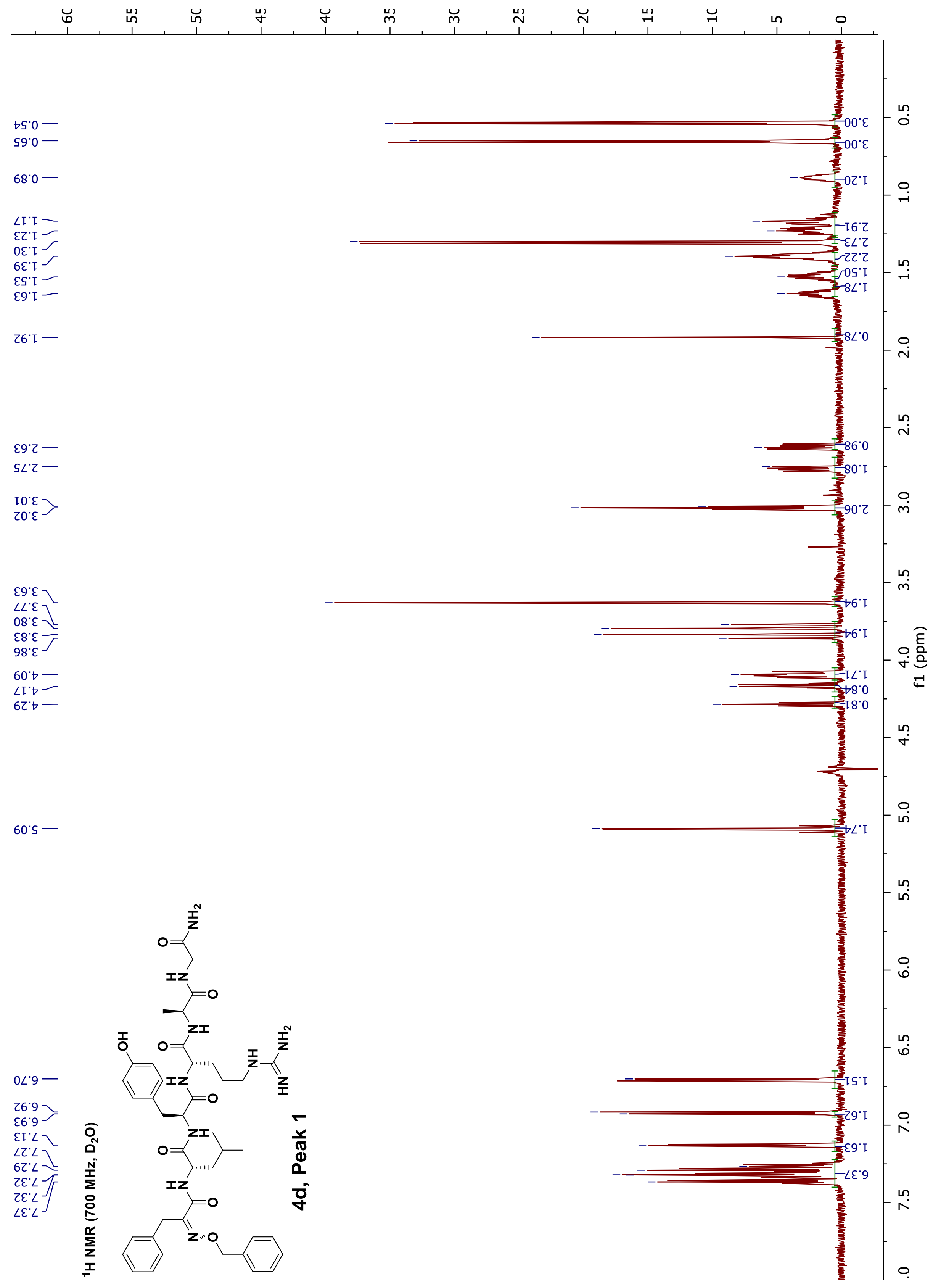

1H NMR (700 MHz, D2O)
4d, Peak 1
f1 (ppm)

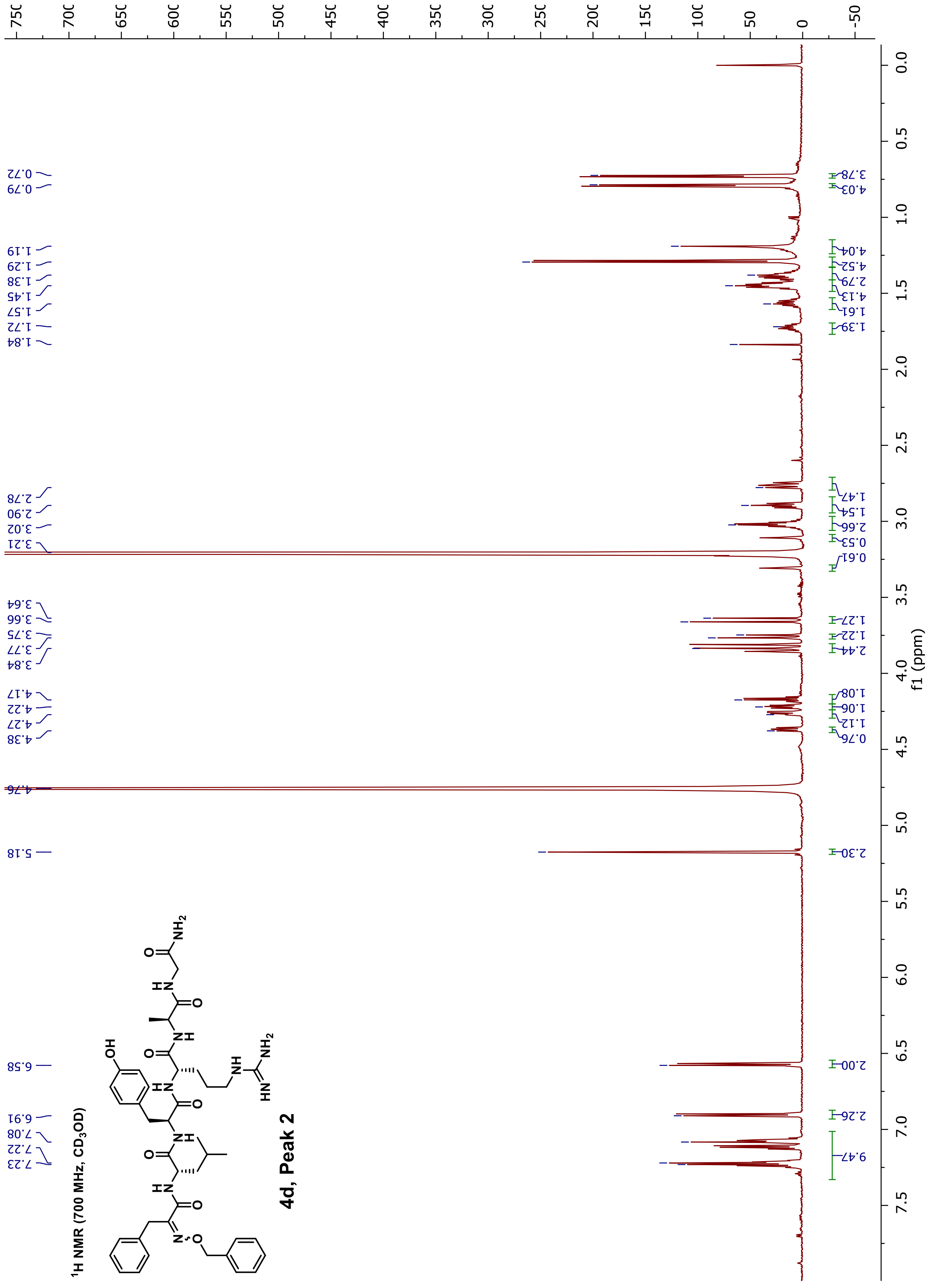

1H NMR (700 MHz, CD3OD)
4d, Peak 2
f1 (ppm)

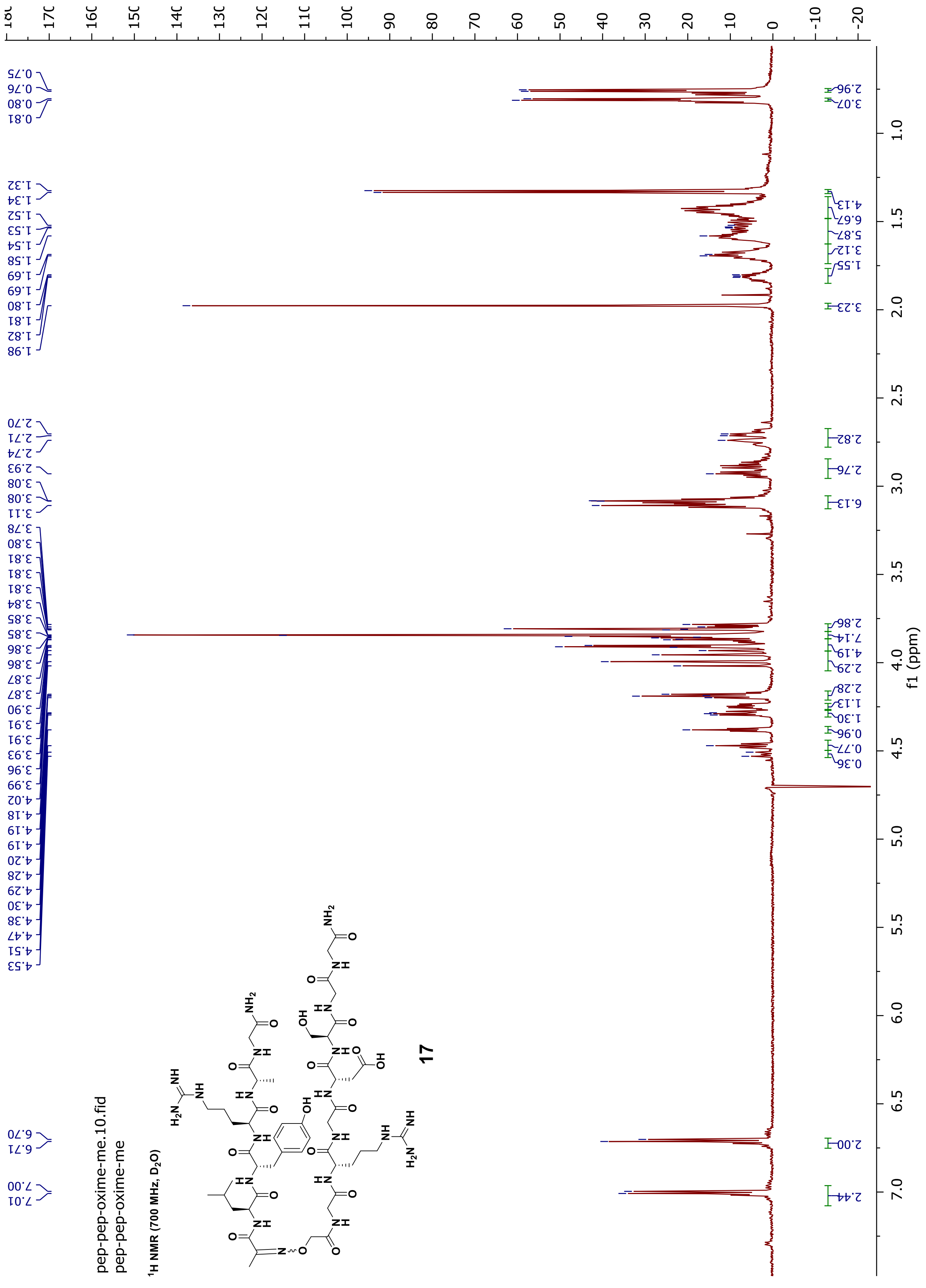

pep-pep-oxime-me.10.fid
pep-pep-oxime-me
¹H NMR (700 MHz, D₂O)
17
f1 (ppm)

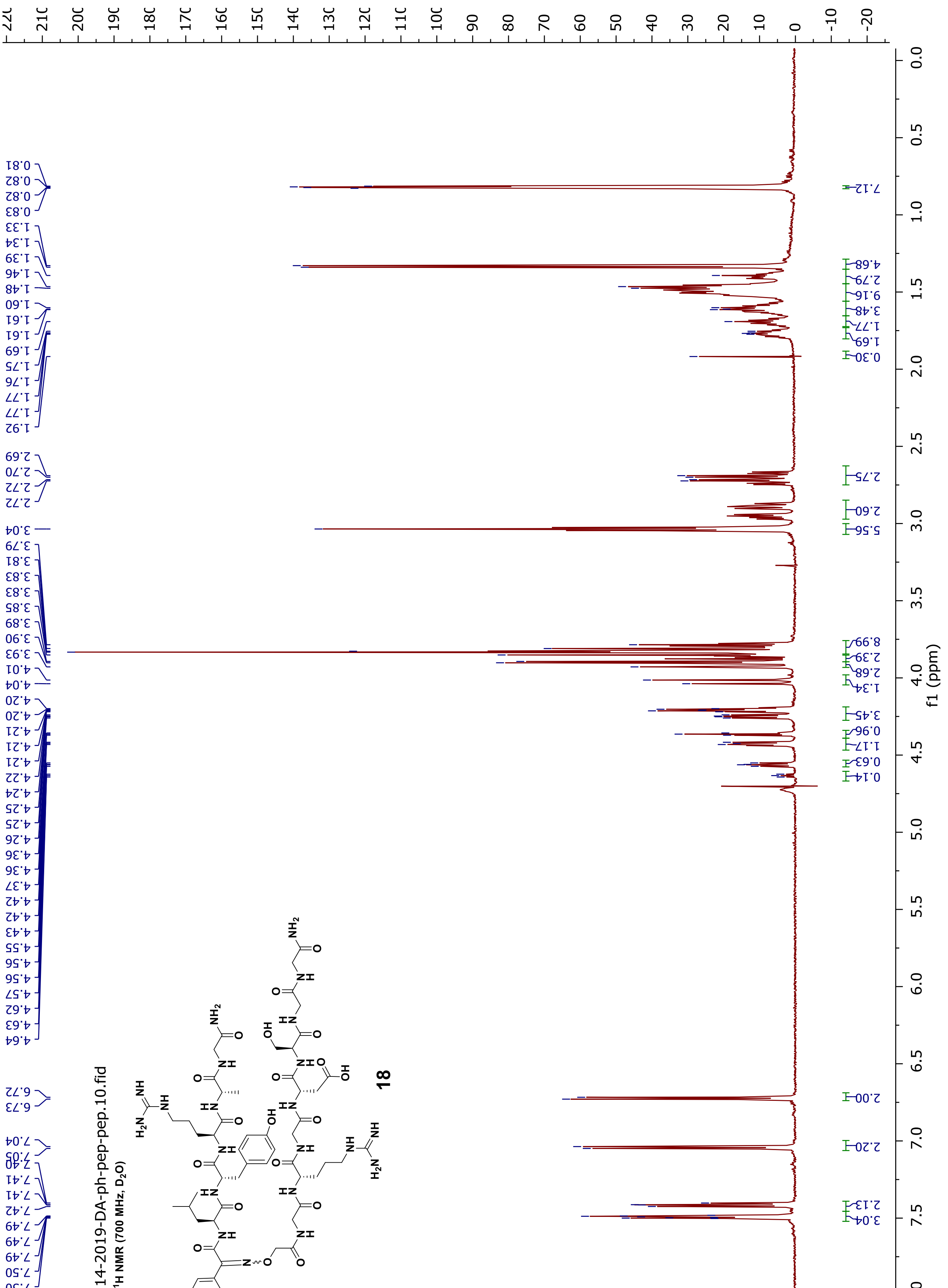

Jul14-2019-DA-ph-pep-pep.10.fid
^{1}H NMR (700 MHz, D_2O)
18
f1 (ppm)

Guthrie, Q. A. E.; Young, H. A.; Proulx, C.

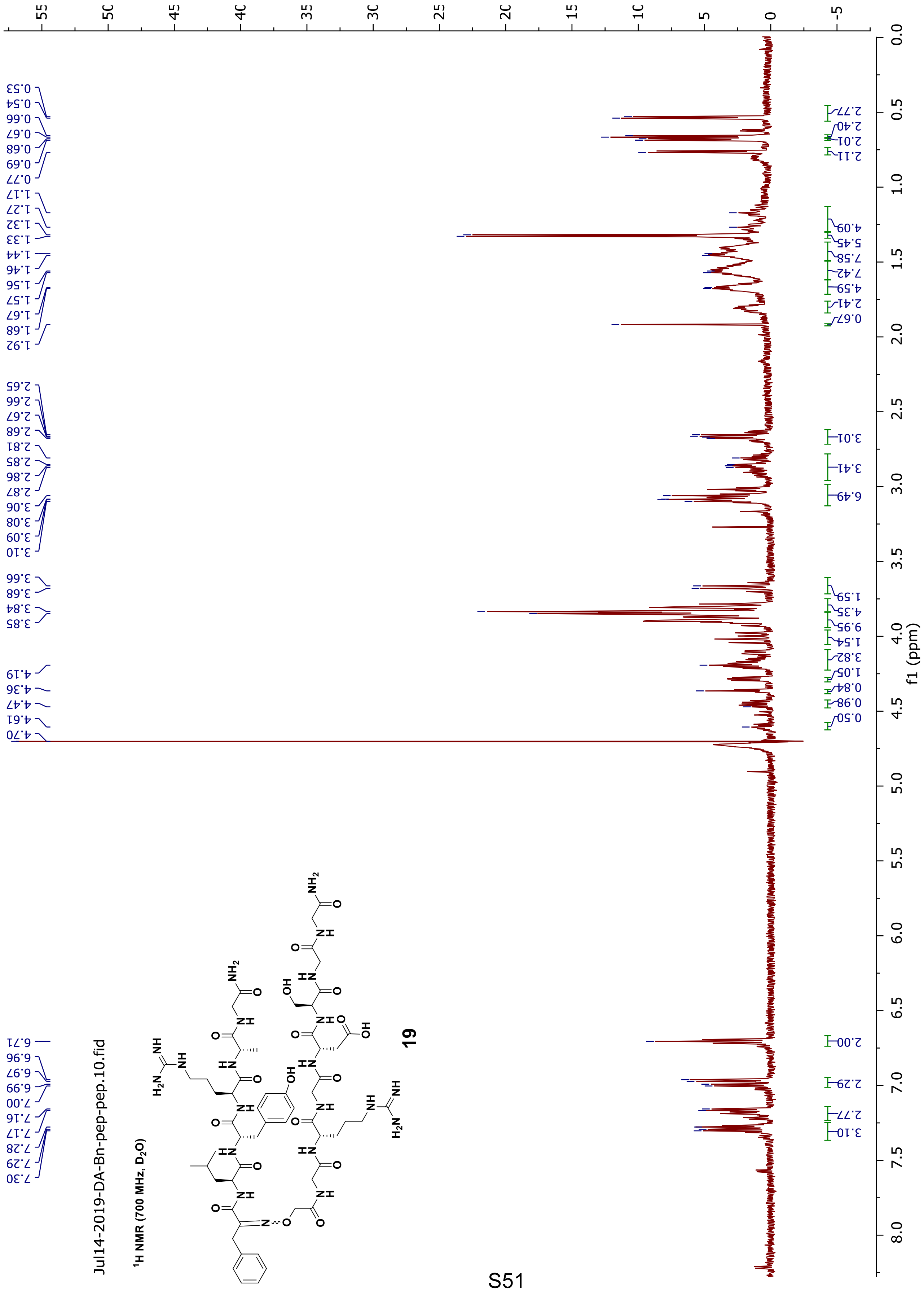

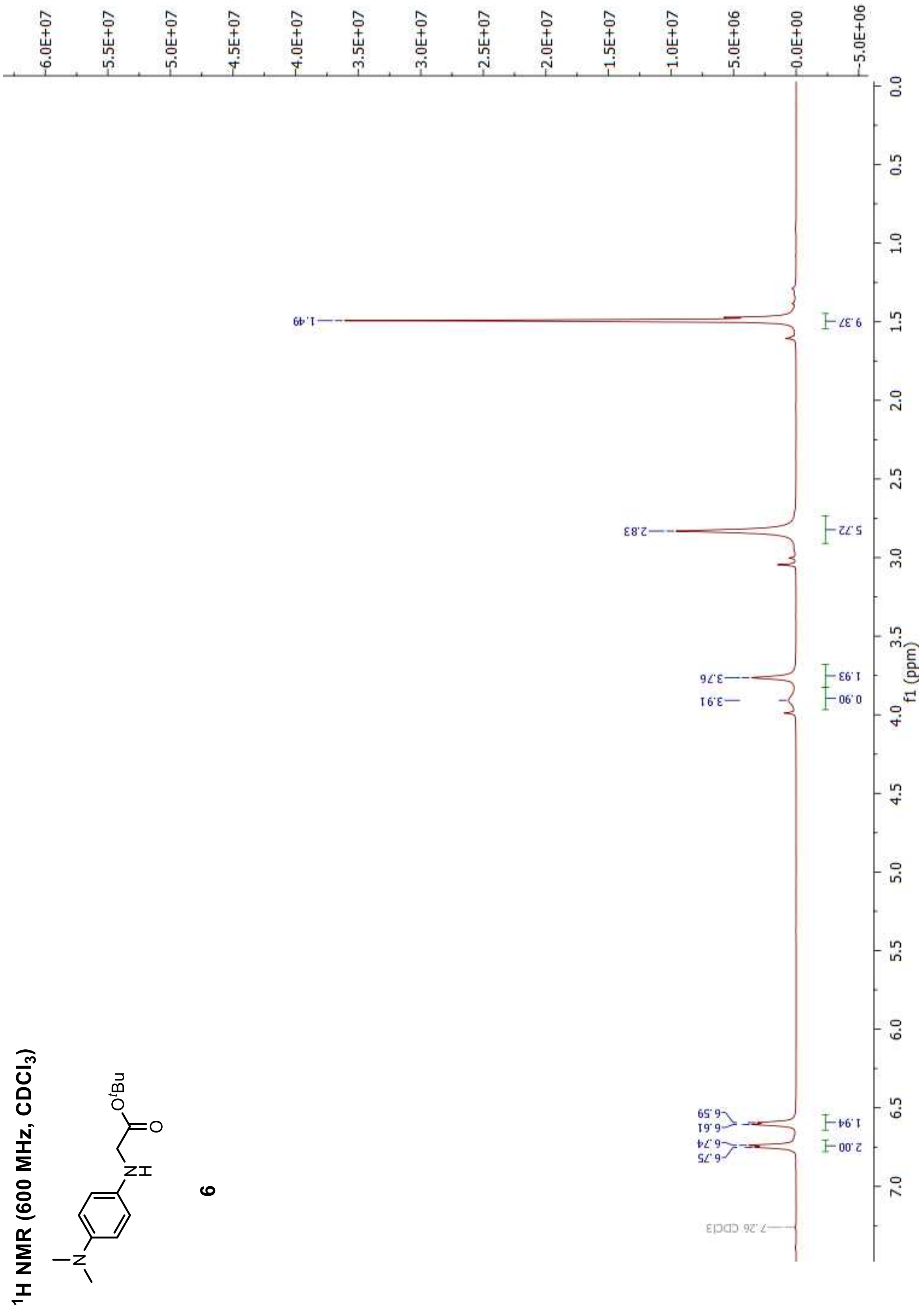

^{1}H NMR (600 MHz, CDCl$_3$)
O^{t}Bu
O
N
H
N
6
1.49
2.83
3.76
3.91
6.59
6.61
6.74
6.75
7.26 CDCl3
9.37
5.72
1.93
0.90
1.94
2.00
f1 (ppm)

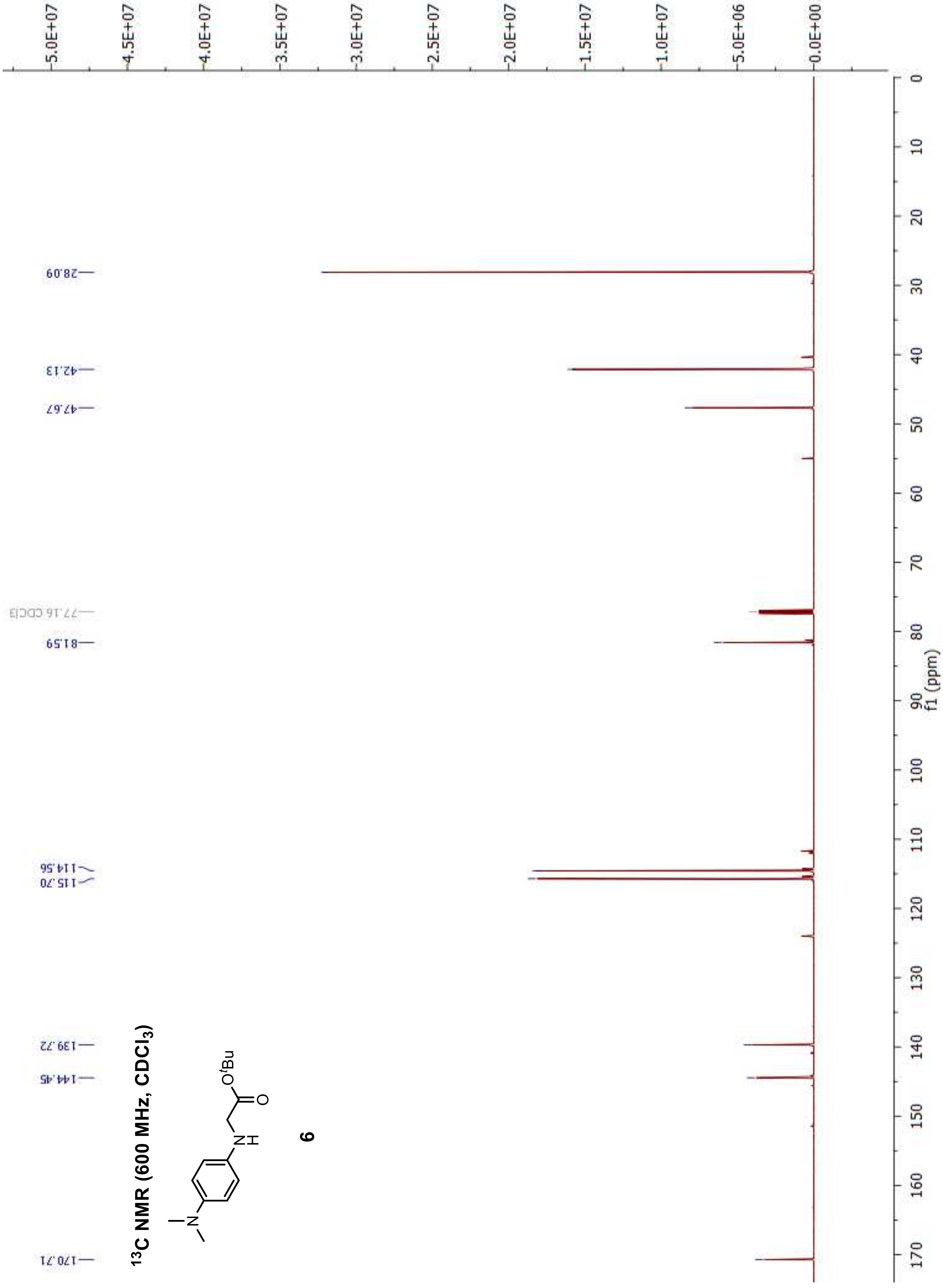
13C NMR (600 MHz, CDCl3)
6
OtBu
O
N
H
28.09
42.13
47.67
77.16 CDCl3
81.59
114.56
115.70
139.72
144.45
170.71
f1 (ppm)

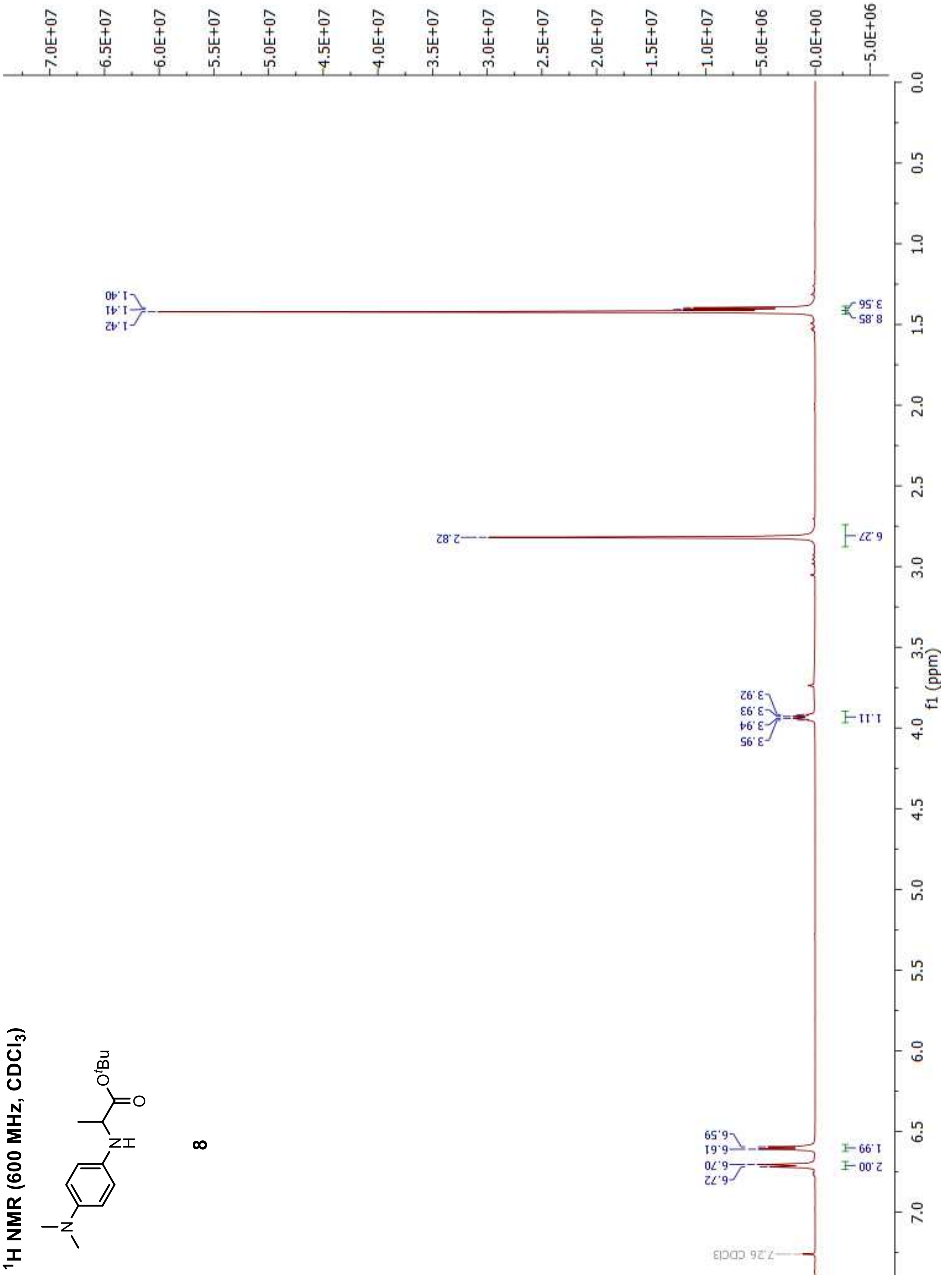

¹H NMR (600 MHz, CDCl₃)
OᵗBu
8
1.40
1.41
1.42
2.82
3.92
3.93
3.94
3.95
6.59
6.61
6.70
6.72
7.26 CDCl3
3.56
8.85
6.27
1.11
1.99
2.00
f1 (ppm)

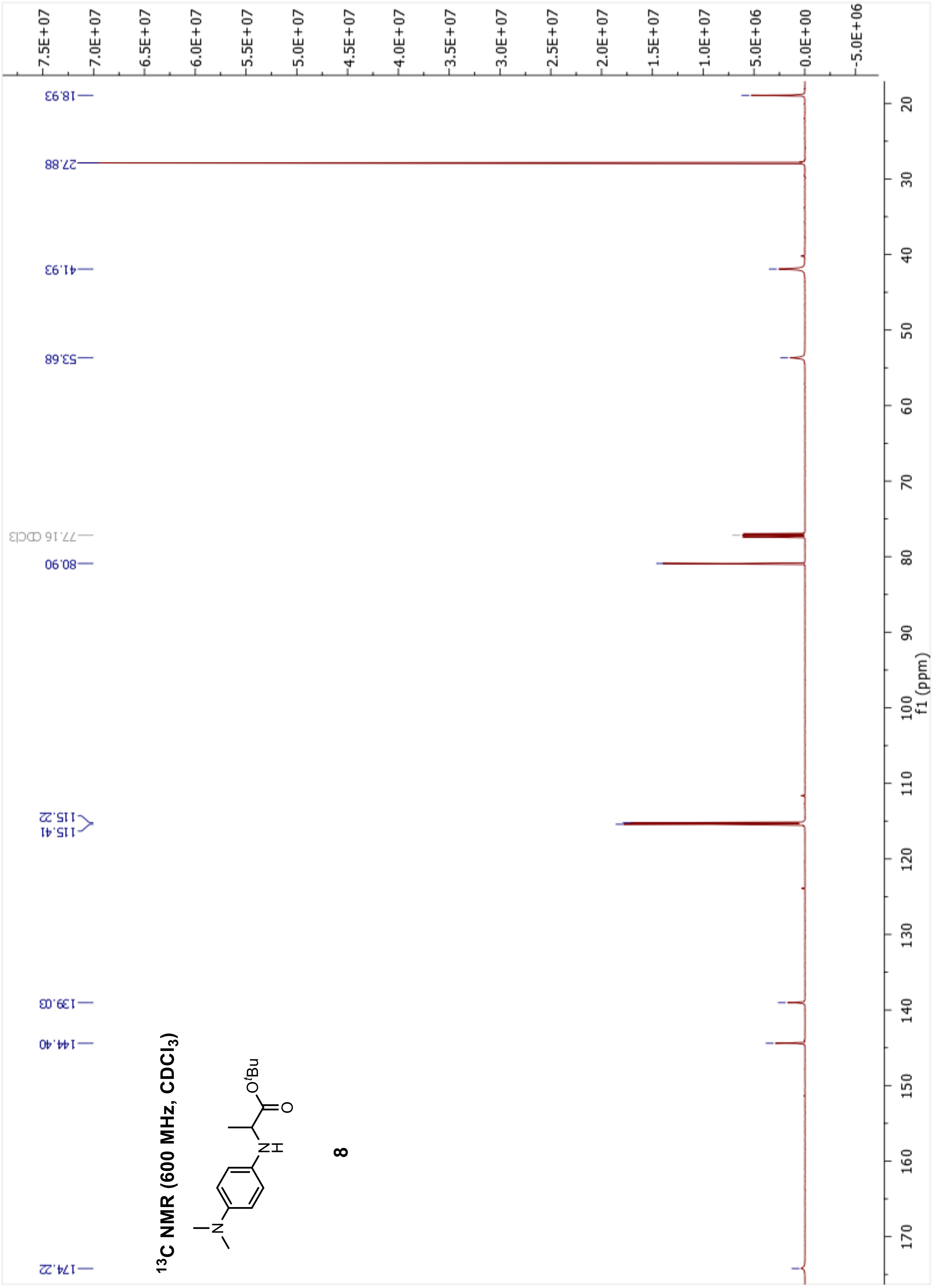

18.93
27.88
41.93
53.68
77.16 CDCl3
80.90
115.22
115.41
139.03
144.40
174.22
f1 (ppm)
13C NMR (600 MHz, CDCl3)
OtBu
8

^{1}H NMR (600 MHz, $CDCl_3$)

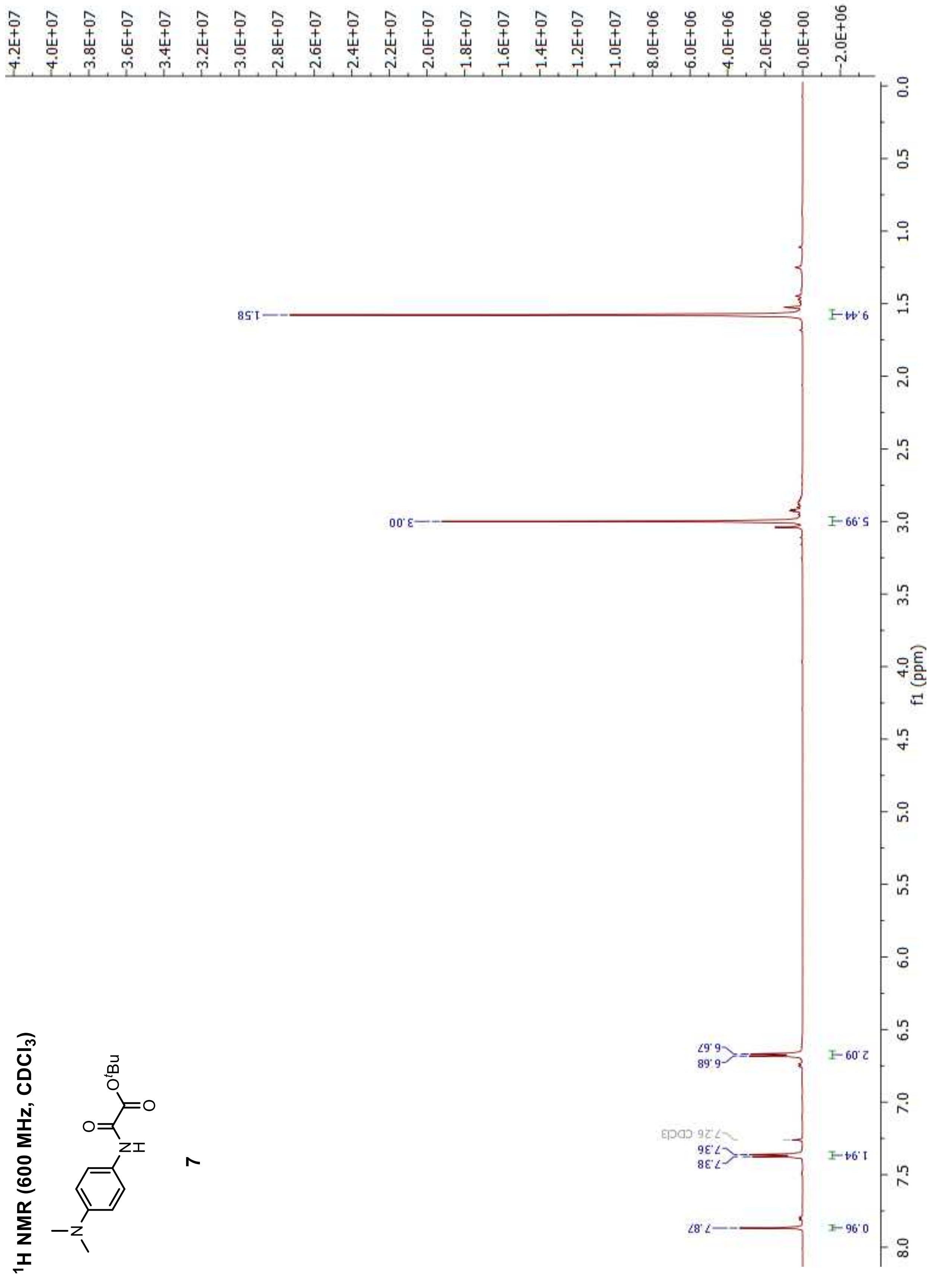

7

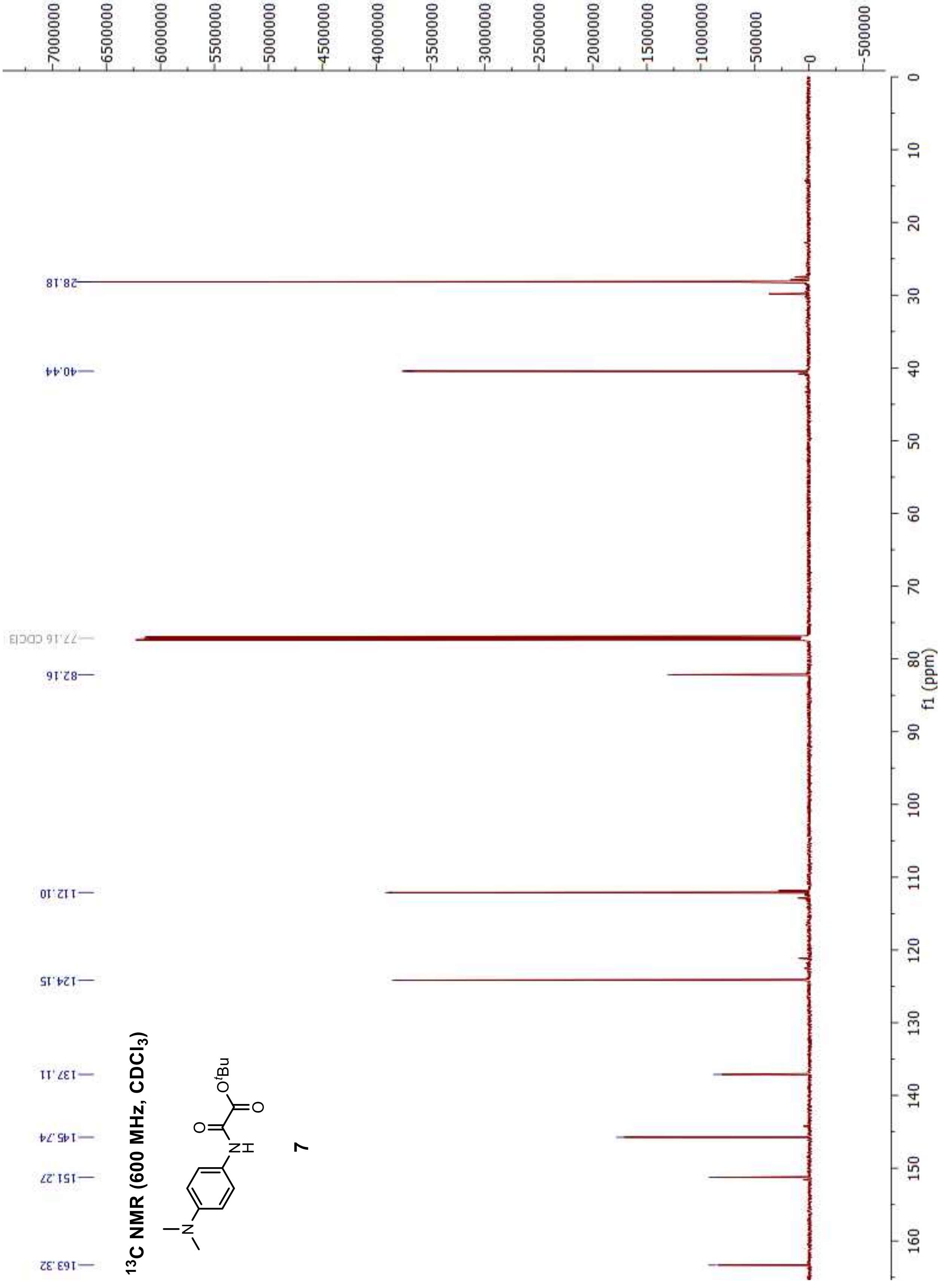
13C NMR (600 MHz, CDCl3)
7
28.18
40.44
77.16 CDCl3
82.16
112.10
124.15
137.11
145.74
151.27
163.32
f1 (ppm)

5. References

1. Q. A. E. Guthrie, C. Proulx, *Org. Lett.,* 2018, **20**, 2564.
2. A. Dirksen, T. M. Hackeng, P. E. Dawson, *Angew. Chem. Int. Ed.,* 2006, **45**, 7581.
3. I. P. Decostaire, D. Levievre, H. H. Zhang, A. F. Delmas, *Tetrahedron Lett.,* 2006, **47**, 7057.
4. G. Mezo, I. Szabo, I. Kertesz, R. Hegedus, E. Orban, U. Leurs, S. Bosze, G. Halmos, M. Manea, *J. Pept.Sci.,* 2011, **17**, 39.
5. M. Vila-Perello, R. G. Gallego, D. Andreu, *Chembiochem* ,2005, **6**, 1831.
6. C. M. Haney, M. T. Loch, W. S. Horne, *Chem. Commun.,* 2011, **47**, 10915.
7. J. E. Reynolds III, M. Josowicz, P. Tyler, R. B. Vegh and K. M. Solntsev, *Chem. Commun.*, 2013, **49**, 7788.
